# Phenotypic and genome-wide studies on dicarbonyls: major associations to glomerular filtration rate and gamma-glutamyltransferase activity

**DOI:** 10.1016/j.ebiom.2024.105007

**Published:** 2024-02-13

**Authors:** Philip Harrer, Julica Inderhees, Chen Zhao, Barbara Schormair, Erik Tilch, Christian Gieger, Annette Peters, Olaf Jöhren, Thomas Fleming, Peter P. Nawroth, Klaus Berger, Marco Hermesdorf, Juliane Winkelmann, Markus Schwaninger, Konrad Oexle

**Affiliations:** a Institute of Neurogenomics, Helmholtz Munich, Neuherberg, Germany; b Institute of Human Genetics, School of Medicine, Technical University of Munich, Munich, Germany; c Institute for Experimental and Clinical Pharmacology and Toxicology, Center of Brain, Behavior and Metabolism, University of Lubeck, Lubeck, Germany; d Bioanalytic Core Facility, Center for Brain, Behavior and Metabolism, University of Lübeck, Germany; e German Centre for Cardiovascular Research (DZHK), Hamburg-Lübeck-Kiel, Germany; f Neurogenetic Systems Analysis Group, Institute of Neurogenomics, Helmholtz Munich, Neuherberg, Germany; g Research Unit of Molecular Epidemiology, Helmholtz Munich, Neuherberg, Germany; h Institute of Epidemiology, Helmholtz Munich, Neuherberg, Germany; i German Center for Diabetes Research (DZD), Neuherberg, Germany; j Chair of Epidemiology, Institute for Medical Information Processing, Biometry and Epidemiology, Medical Faculty, Ludwig-Maximilians-Universität München, Munich, Germany; k Department of Internal Medicine, University of Heidelberg, Heidelberg, Germany; l Institute of Epidemiology and Social Medicine, University of Münster, Münster, Germany; m Munich Cluster for Systems Neurology (SyNergy), Munich, Germany; n German Centre for Mental Health (DZPG), Munich-Augsburg, Germany

**Keywords:** Dicarbonyl compounds, Phenotypic association study, Genome-wide association study, Kidney function, Liver enzymes, Glucose

## Abstract

**Background:**

The dicarbonyl compounds methylglyoxal (MG), glyoxal (GO) and 3-deoxyglucosone (3-DG) have been linked to various diseases. However, disease-independent phenotypic and genotypic association studies with phenome-wide and genome-wide reach, respectively, have not been provided.

**Methods:**

MG, GO and 3-DG were measured by LC-MS in 1304 serum samples of two populations (KORA, n = 482; BiDirect, n = 822) and assessed for associations with genome-wide SNPs (GWAS) and with phenome-wide traits. Redundancy analysis (RDA) was used to identify major independent trait associations.

**Findings:**

Mutual correlations of dicarbonyls were highly significant, being stronger between MG and GO (ρ = 0.6) than between 3-DG and MG or GO (ρ = 0.4). Significant phenotypic results included associations of all dicarbonyls with sex, waist-to-hip ratio, glomerular filtration rate (GFR), gamma-glutamyltransferase (GGT), and hypertension, of MG and GO with age and C-reactive protein, of GO and 3-DG with glucose and antidiabetics, of MG with contraceptives, of GO with ferritin, and of 3-DG with smoking. RDA revealed GFR, GGT and, in case of 3-DG, glucose as major contributors to dicarbonyl variance. GWAS did not identify genome-wide significant loci. SNPs previously associated with glyoxalase activity did not reach nominal significance. When multiple testing was restricted to the lead SNPs of GWASs on the traits selected by RDA, 3-DG was found to be associated (p = 2.3 × 10^−5^) with rs1741177, an eQTL of NF-κB inhibitor NFKBIA.

**Interpretation:**

This large-scale, population-based study has identified numerous associations, with GFR and GGT being of pivotal importance, providing unbiased perspectives on dicarbonyls beyond the current state.

**Funding:**

10.13039/501100001659Deutsche Forschungsgemeinschaft, Helmholtz Munich, 10.13039/100010447German Centre for Cardiovascular Research (DZHK), German Federal Ministry of Research and Education (BMBF).

**Research in context**

**Evidence before this study**

The dicarbonyls methylglyoxal, glyoxal, and 3-deoxyglucosone have been related to various disorders and complications. Their metabolic relations and their roles in disease pathogenesis have remained controversial, however. Remarkably, phenome-wide and genome-wide association studies on dicarbonyls in large-scale human population cohorts are lacking. A PubMed search in August 2023 on “(phenome-wide OR genome-wide OR GWAS) AND (dicarbonyl OR methylglyoxal OR glyoxal OR 3-deoxyglucosone) AND humans” including automatic translations and extensions by the search algorithm retrieved 8 publications which did not fit the actual search target, however.

**Added value of this study**

Studying dicarbonyl levels in 1304 samples from two population cohorts together with a wide array of anthropometric, clinical, and laboratory data allowed for unbiased evaluation of dicarbonyl associations. Numerous associations have been identified. Redundancy analysis revealed glomerular filtration rate, hepatic gamma-glutamyltransferase and, in case of 3-deoxyglucosone, glucose as major contributors to dicarbonyl variance.

**Implications of all the available evidence**

These results provide a solid, wide and unprecedented perspective on the roles of dicarbonyls in health and disease. They put the impact of various factors into perspective, questioning the leading role of glucose but highlighting the importance of hepatic and renal parameters for serum dicarbonyl concentrations.

## Introduction

The dicarbonyls methylglyoxal (MG), glyoxal (GO) and 3-deoxyglucosone (3-DG) are involved in the pathogenesis of degenerative diseases and ageing. Having overlapping metabolic origins and related effects, all three have been assessed together and integrated under the notion of “dicarbonyl stress”.^1^^,^^2^ They glycate macromolecules such as DNA, proteins, and lipids, forming advanced glycation endproducts (AGEs) and may also modify non-covalently the function of macromolecular structures.^1^, ^2^, ^3^ While intake may gain importance if food contains high levels of dicarbonyls^4^ they typically originate from endogenous processes. Degradation of triosephosphate intermediates of glucose metabolism is considered to be a major source of MG, but alternative sources of MG have been emphasized such as glycated protein degradation, acetone oxidation in ketone body metabolism, threonine catabolism, and lipid peroxidation.^1^^,^^5^ Enzymatic repair of glycated proteins is a major source of 3-DG, while GO is generated by lipid peroxidation or degradation of glycated proteins, serine, monosaccharides, and nucleotides.^1^ The glyoxalase system with its two major enzymes glyoxalase 1 (GLO1) and glyoxalase 2 (GLO2) serves to shift MG and GO into D-lactate, while aldo-keto reductases, aldehyde dehydrogenases and AGE formation also contribute to the removal of dicarbonyls.^6^

Abnormal dicarbonyl levels, either as cause or as consequence, have been related to ageing and various diseases including obesity, diabetes, cardiovascular diseases, kidney dysfunction, neurological disorders, and cancer.^1^^,^^7^ However, questions concerning the role of dicarbonyls in pathogenesis remain.^4^^,^^8^ Indeed, moderate increase in plasma MG may even have beneficial effects.^9^

Large-scale population-based analysis of metabolites together with a broad phenotypic assessment may clarify their roles in health and disease. Therefore, we performed a comprehensive investigation of dicarbonyls by highly sensitive and selective LC-MS/MS (liquid chromatography coupled to tandem mass spectrometry) in two German population samples that are well characterized with respect to phenotypic traits including disease history and anthropometric, clinical, and laboratory parameters. Since the genetic bases of dicarbonyl levels in blood also have not been studied sufficiently yet, we combined our investigation with a genome-wide association study (GWAS).

## Methods

### Study populations

#### KORA

Serum samples, genome-wide SNP genotypes and phenome data were available after genotype quality control from 482 unrelated adult individuals of both sexes who participated in the F4 survey of the Cooperative Health Research in the Augsburg Region (KORA) study on the Bavarian population, a regional research platform for population-based surveys.^10^

#### BiDirect

The BiDirect study includes patients with clinically diagnosed acute depression, cardiovascular disease, and a population control group randomly sampled from inhabitants of the city of Münster, Germany. Baseline data collection was done between 2010 and 2013.^11^ For the present study we used the population control group which, after genotype quality control, included serum samples, genome-wide SNP genotypes, and phenome data of 822 individuals.

### Collection of serum samples

All blood samples were collected using the same protocol, except that fasting venous blood was collected between 8 am and 10 am in KORA while non-fasting venous blood was collected in BiDirect. Glucose levels (Table 1) in the BiDirect samples did not differ significantly from those in the KORA samples (p = 0.8 [t-test]), however. After clot formation (30 min at RT), samples were centrifuged at 2,750 g (10 min at 15 °C). After visual check for hemolysis or lipemia to prevent artifacts, the aliquoted samples were stored at −80 °C.

**Table 1:** Distributions of dicarbonyls and lead traits in KORA and BiDirect.

Variable	Type	KORA	BiDirect
Methylglyoxal	continuous	32.47 (27.66–36.91) [nM]	37.53 (32.33–43.85) [nM]
Glyoxal	continuous	199.88 (170.75–233.33) [nM]	129.08 (109.69–148.54) [nM]
3-Deoxyglucosone	continuous	370.62 (312.17–453.67) [nM]	285.87 (239.01–339.78) [nM]
Age Group	categorical	(20, 35]: 8.1% (39) (35, 45]: 19.3% (93) (45, 55]: 20.5% (99) (55, 65]: 19.9% (96) (65, 75]: 20.7% (100) (75, 100]: 11.6% (56)	(25, 35]: 0% (0) (35, 45]: 20.1% (160) (45, 55]: 32.8% (261) (55, 65]: 41.6% (331) (65, 75]: 5.5% (44) (75, 100]: 0% (0)
Sex	binary	male: 49.69% (240) female: 50.31% (243)	male: 50.86% (412) female: 49.14% (398)
Waist-hip ratio	continuous	0.88 ± 0.09	a
BMI	continuous	27.1 (24.6–30.6)	26.2 (23.7–29.4)
Current hypertension (140/90 mmHg) or known hypertension controlled by medication (ISH-WHO 1999)	binary	Yes: 37.3% (180) No: 62.7% (303)	a
Systolic blood pressure	continuous	120.5 (110.25–132.5) [mmHg]	135.5 (126–146) [mmHg]
HDL-cholesterol	continuous	53 (45–64.5) [mg/dl]	58 (47.6–72.2) [mg/dl]
Validated diabetes mellitus	categorical	Type I: 0.2% (1) Type II: 8.7% (42) No: 91.1% (440)	Type I + II: 6.3% (51) No: 93.7% (758)
Serum glucose	continuous	93 (89–102.5) [mg/dl]	95.1 (86.2–104.1) [mg/dl]
hs C-reactive protein (CRP)	continuous	1.26 (0.58–2.94) [mg/l]	0.93 (0.48–2.07) [mg/l]
Glomerular filtration rate (CKD-EPI 2009)	continuous	87.73 (77.43–99.91) [ml/min]	91.25 (79.72–99.28) [ml/min]
Gamma-glutamyltransferase (GGT)	continuous	28 (21–44) [U/l]	31.5 (23.6–42.6) [U/l]
Antidiabetics	binary	Yes: 7.0% (34) No: 93.0% (449)	Yes: 2.2% (18) No: 97.8% (792)
Smoking (pack years > 20)	binary	Yes: 19.9% (94) No: 80.1% (383)	Yes: 8.97% (71) No: 91.03% (720)

Comparison of the characteristics of the lead traits of each associated category that were available in both population samples shown as percentage (and absolute number), mean ± standard deviation, or mean (with interquartile range).

a These traits were not available in BiDirect but had a proxy there which is indicated in the respective row below.

### Liquid chromatography coupled to tandem mass spectrometry

Serum concentrations [nM] of the studied α-dicarbonyls MG, GO and 3-DG were measured using LC-MS/MS as reported before by Rabbani and Thornalley with minor modifications.^12^, ^13^, ^14^ Serum (60 μl) was spiked with 12 μl of the internal standard d4-methylglyoxal (d4-MG, 800 nM (KORA) or 400 nM (BiDirect) in H2O). For protein precipitation, 24 μL of ice-cold trichloroacetic acid (20%, Sigma–Aldrich) was added. After mixing, the sample was diluted with 48 μl water and mixed again. The sample was incubated for 10 min on a ThermoMixer (4 °C/1,000 rpm; Eppendorf), followed by centrifugation (14,000 rpm, 4 °C, 10 min). The supernatant (110 μL) was transferred to a glass vial and α-dicarbonyls were derivatized to the respective quinoxaline compounds with isotopically labelled d8-o-phenylenediamine (CDN Isotopes, 12 μL, 1 mM in 11.6 mM HCl/29.1 μM diethylenetriaminepentaaceticacid (DETAPAC, Sigma–Aldrich)) for 4 h at room temperature in the dark. d4-MG was synthesized using d6-acetone. The concentration and purity of the stock solutions were determined by 13C and 1H NMR at 298 K using a Bruker Avance II NMR spectrometer. The total purity of the d4-MG was 60–65%, the major contaminants being acetate and acetone.

LC-MS/MS analysis was performed on a TSQ Endura triple quadrupole mass spectrometer, equipped with a heated electrospray ionization source, and coupled to a Dionex Ultimate 3000 UHPLC system (ThermoFisher Scientific). Water and acetonitrile, both containing 0.1% formic acid were used as phase A and B, respectively. All solvents were of LC-MS grade quality and were purchased from Merck (Darmstadt, Germany). Quinoxaline derivatives were separated on a Cortecs T3 column (100 mm x 2.1 mm, 2.7 μM; Waters) applying the following gradient with a flow rate of 0.2 ml/min: 5% B for 2 min, with increasing B to 50% between 2 and 7 min and to 100% until 11 min, followed by a washing step with 100% B between 11 and 15 min. A step back to 5% B within 3 min was followed by re-equilibration for 2 min at 5% B. The following parameters were used in the positive ionization mode: ion spray voltage of 4,600 V, vaporizer temperature of 100 °C, and ion transfer tube temperature of 300 °C.

Multiple reaction monitoring (MRM) was used to identify quinoxaline derivatives with collision-induced fragmentation (collision energy 35eV) at 2.5 mTorr using argon. Retention times, MRM transitions and inter-batch variances are listed in Supplementary Table S1. Quantification was performed with an external calibration curve based on the ratio of the areas under the peaks to the internal standard. External standards MG (40%), GO (40%) and 3-DG (75%) were purchased from Sigma–Aldrich. Samples were measured in a randomized order. In every batch, a quality control sample was included in the measurements to calculate inter-batch variability (CV%) and to normalize for inter-batch differences.

### Statistical analyses

#### Correlation between phenotypic data and dicarbonyls

Continuous variables were transformed such as to maximize the explained variance (R2), i.e., log 2-transformed (“log”), subjected to rank-based inverse normal transformation (“irn”) or left untransformed (“raw”). Linear and multivariate linear models were used for regression of traits on single and multiple dicarbonyls, respectively, to calculate significance levels. The assumptions of the regression models were assessed by the Shapiro–Wilk normality test of the residuals and the Cook–Weisburg test for heteroskedasticity. One round of outlier filtering was performed excluding residual outliers (p_Bonferroni_ < 0.05) and data points with a Cook’s distance > 4/N. If the regression assumptions were not met, the transformation type was adapted or, if the latter was unsuccessful, the significance of the Fisher-transformed Spearman rank correlation coefficient was determined. Similarly, the correlation with traits represented by binary, ordinal or nominal variables were analyzed without covariates by simple, ordinal, or multinomial logistic regression models, respectively. If the regression did not converge or outlier diagnostics were still not passed after the first round of outlier filtering, Kruskal–Wallis test was used instead. R^2^ and Nagelkerke’s pseudo-R^2^ were reported for linear and logistic models, respectively.

Traits with the major dicarbonyl associations and the dependencies of other traits’ dicarbonyl associations on them were assessed by stepwise redundancy analysis (RDA) and stepwise conditional analysis (see below).

#### Selection and clustering of traits and definition of lead traits

84 out of 136 phenotypes/traits passed the liberal FDR-threshold of 5% in association with at least one dicarbonyl and were selected for further analyses to simplify the handling of the phenome-wide set of traits. Each of the remaining 84 traits was assigned to one of 21 categories according to general physiological and medical classifications. A trait was defined as the lead trait of a category if in the KORA study the association of that trait reached Bonferroni-corrected significance with at least one dicarbonyl and if that dicarbonyl association was more significant than the respective associations of the other traits in that category. Thus, some categories did not have any significant lead trait while the category “medication” had three.

To ensure that family-wise error rates were not inflated by pre-filtering of traits in the two-stage design, we computed from the original 136 traits the effective number of 63 independent tests using the inverse eigenvalue method.^15^ This number was less than N = 84 which was chosen for Bonferroni correction of the significance threshold to 0.05/84 = 5.95 × 10^−4^ in the second stage.

#### Metric multidimensional scaling, RDA, and stepwise conditional analysis

Metric multidimensional scaling (MDS) and bidirectional stepwise redundancy analysis (RDA) were performed with the *vegan* package (v2.5–7) in R (v4.1.2), where the permutation p-values were set at 0.001 and 0.05 for adding a term to the model and dropping a term from the model, respectively. MDS used type 1 scaling, RDA type 2. Significance of the explanatory variable in the final RDA model was reported as permutation test p-value when larger than 1 × 10^−6^ (corresponding to 999,999 permutations) or approximated by Pillai–Bartlett’s test when smaller. In addition to RDA we performed stepwise conditional analysis in order to discover potential mediation of effects. To do so, we included the most significantly associated trait as covariate in the next analysis step until no trait reached the significance threshold (likelihood ratio test with p < 0.05/84) anymore.

#### Genotyping and imputation

KORA samples were genotyped on the Affymetrix Axiom array, BiDirect samples on the Illumina Psych array. Genotyping and study-center-specific quality control procedures have been described previously.^16^^,^^17^ We excluded individuals if they had a call rate <98% or ambiguous sex calls, pruned related individuals (PIHAT ≥ 0.09375), and removed population outliers (if ≥4SD from population mean in MDS analysis). SNPs were excluded if call rate <95%, MAF < 0.01 or pHWE ≤ 1 × 10^−5^. Imputation was performed with the Sanger imputation server pipeline, using UK10K and 1000 Genomes Phase 3 haplotypes as reference panel, phasing by EAGLE2 (v2.0.5), and imputation by PBWT (v3.1). Post-imputation quality control excluded variants with an info score <0.5.

#### Genome-wide association study (GWAS)

For GWAS we performed linear regression of the three metabolites separately under an additive model in plink 2 (v2.00a3.6LM) on imputation dosage. The baseline GWAS model included age, sex, PCAs, and LC-MS batch as covariates. Since dicarbonyl concentrations appeared to depend on nutrition and excretion, we also used an extended GWAS model, including proxies of them (BMI, glucose, GFR) as covariates besides the baseline covariates. For each metabolite, we combined the KORA and BiDirect GWASs in a meta-analysis (METAL, fixed-effect inverse variance) on variants with MAF > 0.01 and an info score > 0.5 in both studies.

We also tested the extended model with only the 2371 lead SNPs of traits selected in the RDAs of both population samples (Fig. 2) for which GWAS results were available (GFR, GGT, glucose, BMI, and hypertension), downloading the respective summary statistics from MRCIEU API (https://data.bris.ac.uk/data/dataset/pnoat8cxo0u52p6ynfaekeigi) and harmonizing them with TwoSampleMR v0.4.26. As some of these lead SNPs were in linkage disequilibrium, we calculated an effective number of 1890 for Bonferroni-correction.^15^

#### Role of funders

The funding sources did not play any role in study design, data collection, data analyses, interpretation, writing of report, and decision to submit for publication.

#### Ethics

All participants gave informed consent. Study protocols have been approved by the responsible local ethics committees. The KORA study was approved by the Ethics Committee of the Bavarian Medical Association (# 99186) and the Bavarian commissioner for data protection and privacy. The BiDirect study was approved by the Ethics Committee of the University of Münster (# 2009-391-f-S) and the Westphalian Chamber of Physicians.

## Results

### Phenotypic association study

**Fig. 1:**
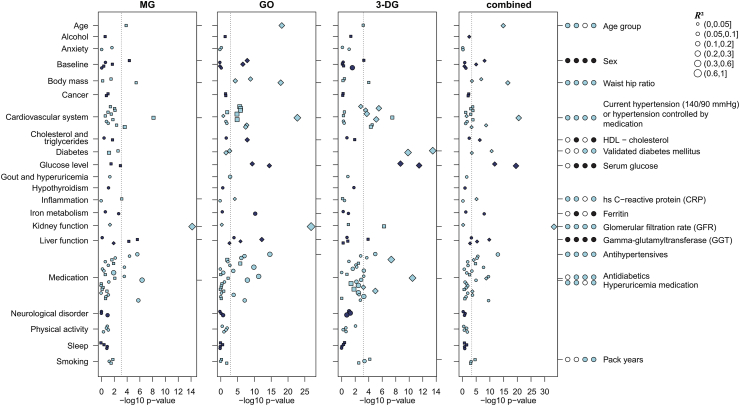
Phenotypic associations of dicarbonyl compounds. Manhattan plot of associations with 84 phenotypic traits (Supplementary Table S3) in 21 categories listed on the left. The significance of association in the KORA population sample is shown on the x-axis. Symbol size indicates the strength of the association measured by R^2^ as outlined in the top right corner. Dotted lines indicate the Bonferroni-corrected significance threshold of 0.05/84 = 5.95 × 10^−4^. In each panel, circles indicate availability of the traits in KORA only, squares indicate that the traits (or their proxies) were available in BiDirect but without replicating significance, and diamonds indicate traits were significantly replicated in BiDirect. The lead trait of each category is reported on the right, with the four circles corresponding to the four panels MG, GO, 3-DG and their combination, filled circles indicating significance (p_Bonferroni_ < 0.05) in testing for association with the respective dicarbonyl in univariate or multivariate regression (“combined”).

After quality control, MG, GO and 3-DG serum concentrations of 482 KORA and 822 BiDirect samples were available for statistical analysis. The phenotypic association study started with 136 potentially relevant traits including sociodemographic data, body measures, medical history, laboratory parameters, and medication (Supplementary Table S2) available in KORA. 84 of these traits passed the false discovery rate (FDR) threshold of 5% for at least one dicarbonyl compound and were included in further analysis. 15 of the remaining 84 traits were significantly (p_Bonferroni_ < 0.05) associated with MG, 33 with GO, and 26 with 3-DG after multiple testing correction (Fig. 1 and Supplementary Table S3). We grouped the 84 significantly associated traits into 21 categories according to general physiological and medical classifications (see list at the left side of Fig. 1 and Supplementary Fig. S1). For categories containing significantly associated traits, we defined lead traits, mostly one per category, according to the highest significance of association with any of the three metabolites. Thereby, 15 lead traits were assigned to the 21 categories.

**Fig. 2:**
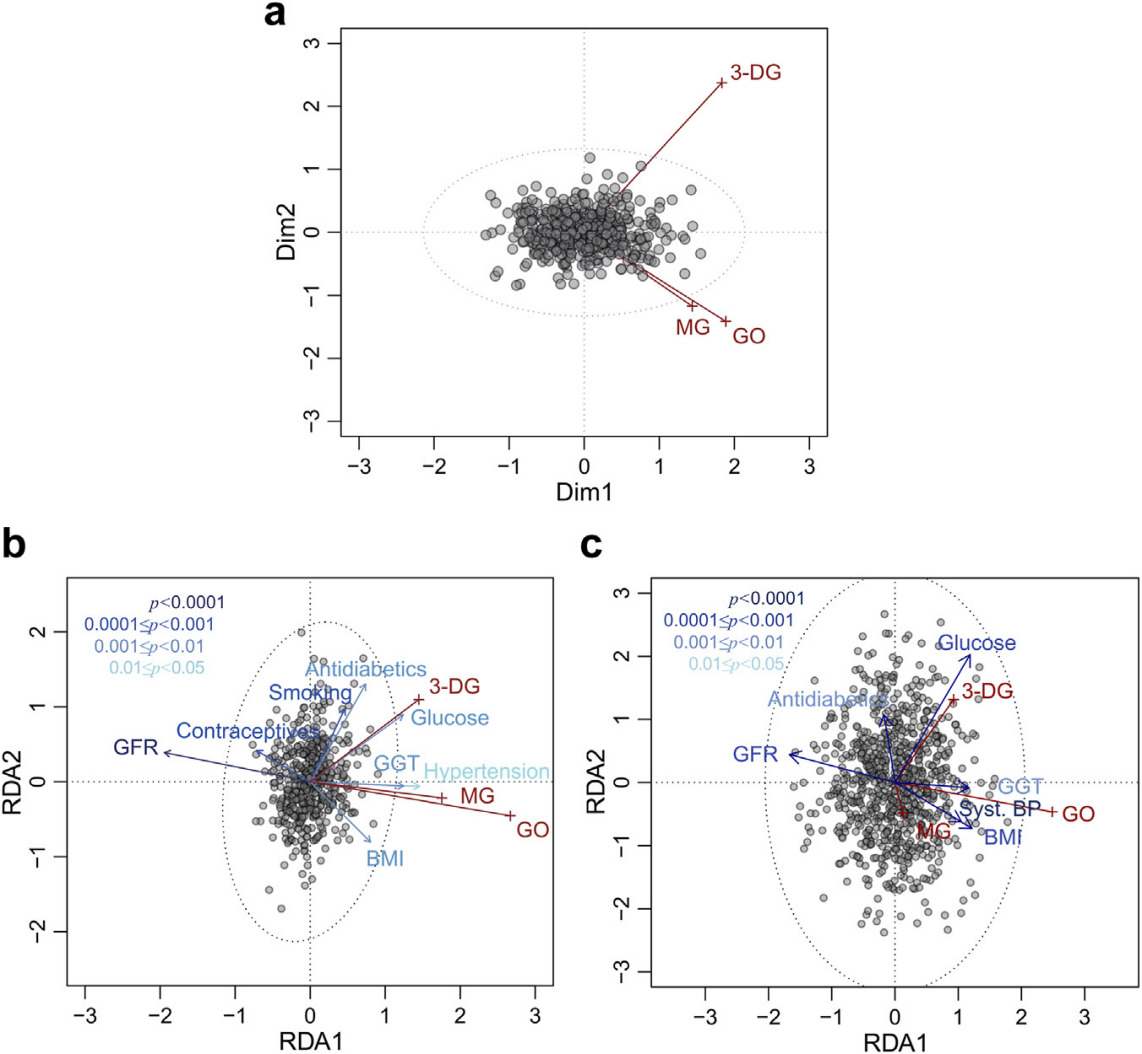
Multidimensional scaling and redundancy analysis. **a:** Two-dimensional MDS representation of MG, GO, and 3-DG levels (log2-scale) in KORA. The distance between two points indicates their dissimilarity in terms of the three metabolites. Lengths and orientations of the metabolite vectors indicate their relative effects on the MDS representation and the correlation of these effects, respectively, with no correlation in case of orthogonal orientation and maximal (anti)correlation in case of parallel orientation. **b and c:** Triplot (type 2 scaled) representations of the multivariate redundancy analyses in KORA (**b**) and BiDirect (**c**), respectively. Vectors indicate relative effects and correlations as in (a) with response variables (i.e., the three metabolites) in red and explanatory variables in different shades of blue according to the significance level of their effect. Prominently, the diagrams show the strong effect of GFR on MG and GO and of glucose on 3-DG.

Significantly associated traits were checked for replication in BiDirect. Matching data was available for 44 of the 84 KORA phenotypes (23 identical traits, 21 proxies; see Fig. 1 and Supplementary Table S3). For GO and 3-DG, most significant associations in KORA were confirmed in BiDirect (16/22 of matching traits for GO, 12/20 of matching traits for 3-DG), while for MG only the top hit, GFR, was replicated (p_Bonferroni_ < 0.05).

12 of the 15 lead traits were available in both population samples (exactly or as a proxy). Their characteristics were comparable (Table 1). 11 out of the 12 available lead traits could be replicated in BiDirect (Fig. 1). Meta-analysis of the lead traits (Supplementary Table S4) confirmed all significant associations.

We observed significant positive correlation between the three dicarbonyls, where MG and GO showed the strongest correlation (ρ = 0.62, p = 2.23 × 10^−52^ [Pearson correlation of the logarithmic values and significance testing after Fisher transformation]), while 3-DG showed a moderate correlation to MG (ρ = 0.39, p = 2.20 × 10^−19^) and GO (ρ = 0.41, p = 2.97 × 10^−21^) (Supplementary Fig. S2). Therefore, we also performed a combined analysis of all three dicarbonyls by multivariate regression on all phenotypes individually. This approach revealed significant associations (p_Bonferroni_ < 0.05) with traits in the categories age, sex, body mass, cardiovascular system, cholesterol and triglycerides, diabetes, glucose metabolism, inflammation, iron metabolism, kidney and liver function, medication, and smoking (Supplementary Table S3).

### Metric multidimensional scaling, RDA, and stepwise conditional analysis

Multidimensional scaling analysis indicated a unimodal, central distribution of the three metabolites, with no clustering or stratification, and no outliers (Fig. 2a). The common effect of the three metabolites as represented by the first dimension explained 64.7% of the total variance. The difference of 3-DG to MG and GO corresponded to the second dimension and explained 24.9% of the total variance (Fig. 2a).

To identify the traits underlying the observed direction of correlation a redundancy analysis (RDA) was performed. In KORA (Fig. 2b), MG and GO showed strong negative correlation with GFR (cos (178.3° ± 1.86°) = −0.998 ± 0.001) while the correlation with GGT, a marker of liver function, was strongly positive (cos (7.0° ± 1.87°) = −0.994 ± 0.003). 3-DG had nearly maximal positive correlation with glucose (cos (1.31°) = 1.00). GFR and GGT together explained 20.2% of the total variance of the three metabolites and 76.1% of the total explainable variance (R^2^ = 0.261). Considering only the two metabolites MG and GO, 32.2% of their total variance was explained by the RDA model, with GFR + GGT accounting for 86.7% and glucose for 21.0% of the total explainable variance (Note that glucose and GFR/GGT are not completely independent. Therefore, the sum of 86.7% and 21.0% is larger than 100%). For 3-DG alone, 17% of the total variance was explained by the RDA model, with GFR + GGT explaining 41.7% and glucose explaining 43.0% of that proportion.

In BiDirect (Fig. 2c) the relation of GO to GFR and GGT was clearly replicated with a strong negative correlation in case of GFR (cos (176.3° ± 7.60°) = −0.998 ± 0.017) and a strong positive correlation in case of GGT (cos (14.5° ± 11.4°) = 0.968 ± 0.068). For MG, however, the replication was less obvious with an angle of 62.9° ± 25.8° between the vectors of MG and GO. For 3-DG, the very strong positive correlation with glucose was clearly replicated again (cos (9.0° ± 5.3°) = 0.988 ± 0.019).

Further influences on dicarbonyl levels as identified by RDA in KORA were hypertension, BMI, antidiabetics, contraceptives, and smoking (Fig. 2b). They replicated in BiDirect except for contraceptives (not available) and smoking (Fig. 2c). While these influences were comparatively small and more or less strongly correlated with the major influences GFR, GGT and glucose, they still appeared to have independent associations with (some of) the dicarbonyls.

Stepwise conditional analyses were performed to assess whether/how the most strongly correlated traits affected others’ dicarbonyl correlations (Supplementary Table S5). These results were in keeping with the RDA results.

### Genome-wide association study (GWAS)

We used existing genetic data to conduct genome-wide association studies (GWASs) for the three metabolites. A total of 1272 samples (450 KORA, 822 BiDirect) and 5,231,190 variants were analyzed. We performed GWAS for each metabolite in each population sample using a baseline model and an extended model controlled for intake (glucose levels and BMI) and excretion (GFR), followed by meta-analysis of the two population samples.

**Fig. 3:**
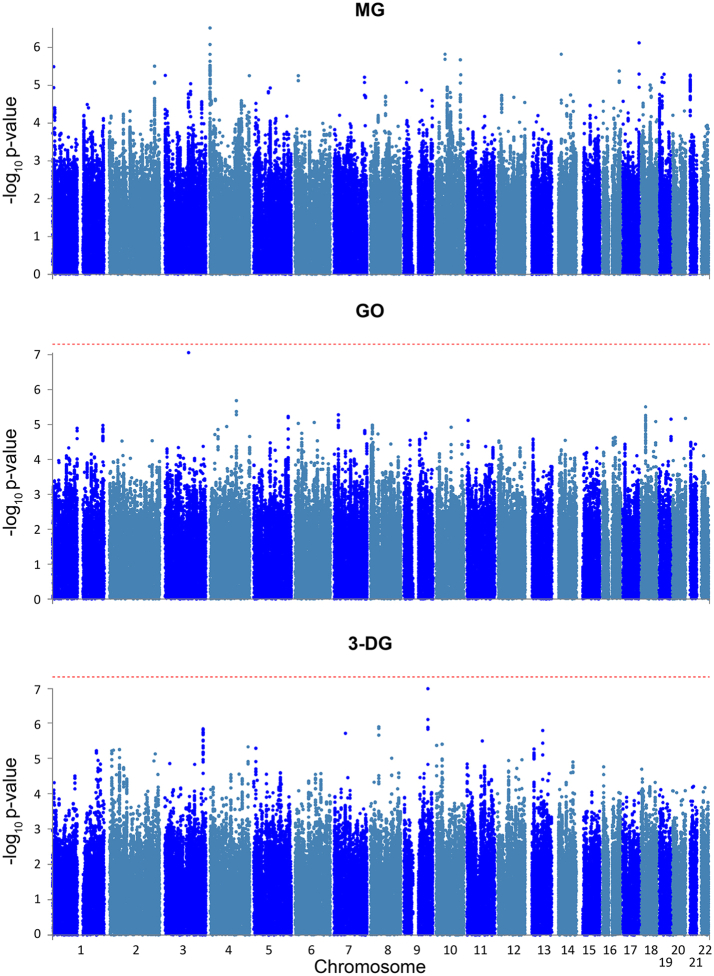
Manhattan plot of GWAS on dicarbonyl metabolites. Meta-analysis of the GWASs in KORA and BiDirect on the three dicarbonyl metabolites controlled by BMI, age, sex, GFR, glucose, batch and the first 10 PCs as covariates.

There were no genome-wide significant signals, neither in the individual GWAS nor in the meta-analyses (Fig. 3; Supplementary Fig. S3). This was specifically true for 360 SNPs at the *GLO1* locus (p > 0.01) and 193 SNPs at the *GLO2* locus (p > 0.09). None of the 4 SNPs at the *GLO1* locus,^18^, ^19^, ^20^ rs4746 (Ala111Glu), rs1781735, rs1130534, and rs1049346, which were previously assumed to affect GLO1 activity, reached nominal significance when tested for associations with MG or with GO.

Therefore, we restricted the association analysis (extended model) to the set of lead SNPs of traits selected in the RDAs of both population samples (Fig. 2b and c) for which GWAS results were available: GFR,^21^ BMI,^22^ glucose,^23^ systolic blood pressure,^24^ and GGT.^25^ The significance threshold was thereby relaxed to a threshold of p_Bonferroni_ = 2.65 × 10^−5^, revealing an association of 3-DG with the GFR-GWAS lead SNP rs1741177 (p = 2.33 × 10^−5^; Supplementary Table S6). Effect directions were opposite to each other, in keeping with the anti-correlation of GFR and 3-DG. As the model included GFR as covariate, we avoided merely picking up the GFR association of rs1741177. Steiger’s directionality test indicated that this SNP was more likely to affect GFR via 3-DG than 3-DG via GFR (p = 0.0011). In the one-sample-based MR analysis, rs1741177 was a rather weak instrument variable (p = 0.00092), however.

## Discussion

We report a comprehensive analysis of dicarbonyls in relation to body measures, medical history, and laboratory parameters in two independent German population samples, providing a large-scale, population-based, disease-independent study of dicarbonyls together with a broad range of potentially interacting traits. We assessed the dicarbonyls MG, GO and 3-DG as well as 136 traits in one population-based study (KORA) and replicated the results in another independent study (BiDirect). Serum dicarbonyl concentrations were measured using an established, state-of-the-art LC-MS/MS method.^12^^,^^14^ To optimize the analytic procedure, we employed several measures, including standardized sample collection, storage of samples at −80 °C, cooling and acidification of samples reducing the formation of dicarbonyls during pre-analytic processing, a stable isotope-labelled internal standard and derivatization reagent. With this, measured dicarbonyls concentrations were lower than in other studies indicating that we avoided overestimation which is considered the main analytic problem.^26^ Pairwise correlations of the dicarbonyls were positive and highly significant with the correlation between MG and GO being the strongest.

Reactive dicarbonyls have been linked to several age-related risk factors and diseases.^1^^,^^2^^,^^7^ However, because of the limited number of parameters included in previous studies the relative importance of various associations remained unclear. The key strength of our study is the simultaneous assessment of a large range of parameters in the same individuals. RDA allowed us to determine the major influences on the total variation of the three dicarbonyls in blood. In both population samples, the main influence on MG and GO was the glomerular filtration rate (GFR), whereas blood glucose level was the main influence on 3-DG.

Increased plasma MG levels have previously been associated with a reduction of the GFR in patients with chronic kidney disease.^27^, ^28^, ^29^ Dicarbonyl stress may impair kidney function in various ways including NF-kB activation and inflammation of glomerular mesangial cells via the AGE receptor RAGE^30^^,^^31^ but could also be the consequence of reduced renal clearance, potentially related to an assumed downregulation of the glyoxalase system in chronic kidney disease.^32^^,^^33^ Both may be true, thus constituting a vicious circle of dicarbonyl accumulation and impaired renal clearance. If so, early kidney-friendly intervention such as blood pressure control could potentially lower dicarbonyl stress.

Glucose concentrations or diabetes were expected to influence the dicarbonyls since they are directly or indirectly (degradation of glycated molecules) derived from glucose. Indeed, diabetes was the first disease for which increased formation of MG and MG-derived AGEs was reported.^34^^,^^35^ Interestingly, 3-DG was more strongly associated with glucose than GO and MG in our study. The association of MG and glucose was only borderline, in fact. This recalls earlier doubts about a primary, direct relation between glucose and MG in plasma.^5^

Age showed a highly significant association with GO and significant age associations were also found for MG and 3-DG, at least in the KORA sample. However, age was not selected in the RDA, and in association analyses conditioned on GFR and other traits of major influence (Supplementary Table S5) none of the three dicarbonyls showed any residual association with age. This corroborates recent results suggesting that age alone does not necessarily imply an impairment of dicarbonyl metabolism.^36^ Together, the multiple associations presented here suggest that future studies on the dicarbonyl-related pathophysiology should measure all three dicarbonyls simultaneously since despite their correlation they do have different association spectra. Moreover, such studies need to account for covariates, with kidney function (GFR), liver status (GGT),^37^ and glucose metabolism being most important.

Beyond the replication and refinement of known associations we also identified associations that have not been described before:

In the KORA sample, data on intake of contraceptives was available and showed negative correlations with serum MG and GO levels that did not appear to be redundant with other available traits (Fig. 2b). Stepwise conditional analysis indicated that the association with GO vanished, however, when it was conditioned on the more significantly associated traits GFR, BMI, GGT, and hypertension.

Serum 3-DG concentrations correlated with smoking which was highly significant in the KORA sample, with the same direction nominally significant in BiDirect, and significant in the meta-analysis. RDA suggested that this association is not redundant with major influences such as GFR. When the association analysis was conditioned stepwise on GFR and glucose, smoking intensity (pack years) remained significant. The next trait included in the stepwise conditional analysis was additional passive smoking. Only thereafter smoking intensity lost nominal significance (p = 0.054; Supplementary Table S5). It remains to be determined, therefore, whether there is a direct effect of smoking on the 3-DG level.

Furthermore, we observed a significant association of ferritin to GO and, with borderline significance, to MG. Serum ferritin is an acute phase reactant being increased in inflammation.^38^ Indeed, GO and MG were also associated with C-reactive protein (CRP), a widely used inflammation marker. The associations of dicarbonyls with the two inflammation markers may have been mediated at least in part by the strongly associated GFR since the latter is known to correlate with both.^39^, ^40^, ^41^ Indeed, ferritin and CRP were not selected by RDA, and when we conditioned their association analyses on GFR, waist-to-hip-ratio, GGT, and hypertension, both lost their significance (Supplementary Table S5).

Dicarbonyl metabolism might be influenced by genetic predisposition. Therefore, we conducted GWAS for the three dicarbonyls despite the limited sample size of 1272 individuals. No genome-wide significant signals were found, suggesting that there are no common SNPs (minor allele frequency ≥1%) with sufficiently strong effects on MG, GO, or 3-DG. Specifically, we did not find any evidence of the association of MG (or GO) with the *GLO1* or *GLO2* loci. The SNPs at the *GLO1* locus which previously have been assumed to affect GLO1 activity,^18^, ^19^, ^20^ did not show nominally significant associations with MG or GO. However, we cannot exclude small effects of variants in these genes below the power limit of our study.

When we relaxed the multiple testing burden, i.e., the significance threshold, by restricting the association analysis to the set of candidate SNPs that had been lead SNPs of previous GWASs on the major correlated traits, we identified a significant association of the GFR lead SNP rs1741177 with 3-DG (but not with MG or GO). The effects of this SNP’s minor allele on GFR and 3-DG had opposite directions, in keeping with the anti-correlation of the two traits. According to the MR-Base platform,^42^ rs1741177 is an eQTL of several genes that relate to immune regulation such as *FAM177A1*,^43^ *PSMA6*,^44^ *PPP2R3C*,^45^^,^^46^ and *NFKBIA*.^47^ The minor allele of rs1741177 reduces *NFKBIA* expression and therefore likely increases NF-κB activity. It remains to be seen how this would relate to the previously described AGE-induced NF-κB activation.^48^

Our study has some limitations. The study design is cross-sectional which generally precludes a definite derivation of causal phenotypic relations. We observed lower correlations and slightly different distributions of the three dicarbonyls in BiDirect as compared to KORA. This was most pronounced for MG, explaining the less successful replication in BiDirect of the MG associations. The differences between the two studies may be due to several factors acting together: Dicarbonyl measurements were performed in the same laboratory on the same machine but at different times, the other metabolic traits were measured by different laboratories, and the study participants differed in fasting state and slightly in age, sex, and disease frequencies. Despite these differences, the key explaining factors for the dicarbonyl levels were the same, strengthening the robustness of our results. Though this study is the so far largest population-based study on dicarbonyls in human blood, the sample size was underpowered to dissect the genetic contributions to dicarbonyl metabolism. Thus, we were not able to assess the heritabilities and genetic correlations of the dicarbonyls as reliable estimates would require much larger sample sizes.^49^ This calls for meta-analysis together with other pertinent GWASs as soon as they are available.

Overall, in a general population setting, GFR was the most important trait linked to MG and GO levels, while glucose was closely associated with 3-DG concentrations. The three dicarbonyls are not only interesting players in the pathophysiology of diseases, but also useful biomarkers for therapeutic intervention studies. Our results prioritize biochemical pathways to be targeted in order to reduce dicarbonyl stress in ageing and disease. Larger GWAS meta-analyses will help to better understand the genetic bases of dicarbonyl metabolism.

## Contributors

All authors contributed to data acquisition and data analysis. PH, JI, KO, BS, MS, JW and CZ contributed to study concept and design. JI and MS designed and performed the dicarbonyl measurements. PH, KO, BS and CZ performed the statistical analyses and drafted the manuscript. PH, JI, CZ, BS, OJ, KB, MH, MS and KO have accessed and verified the underlying data. All authors read and approved the final version of the manuscript.

## Data sharing statement

All significant results are provided in the Supplementary Appendix of the published manuscript. Project agreements to use and access individual-level KORA data can be requested by approved researchers via the KORA-PASST tool under https://helmholtz-munich.de/epi. Individual-level data of the BiDirect Study can be requested by approved researchers from KB (https://medizin.uni-muenster.de/en/epi). Data transfer is based on a written transfer agreement.

## Declaration of interests

Authors have no competing interest to declare.
