# Supplementary material for: Phenotypic and genome-wide studies on dicarbonyls: major associations to glomerular filtration rate and gamma-glutamyltransferase activity
Source: eBioMedicine. 2024 Feb 13;101:105007. doi: 10.1016/j.ebiom.2024.105007 (PMC10875252; doi:10.1016/j.ebiom.2024.105007)
Supplement: Supplementary Figs. S1–S3 and Tables S1–S6 [file mmc1.pdf]

# Supplementary Material

## Supplement to

Phenotypic and genome-wide studies on dicarbonyls: major associations to glomerular filtration rate and gamma-glutamyltransferase activity

# Supplementary tables and figures – overview

## Supplementary figures:

### Supplementary figure 1
Hierarchical clustering of dicarbonyl-associated traits.

### Supplementary figure 2
Correlation plots of MG, GO, and 3-DG.

### Supplementary figure 3
QQ-plots for GWAS of dicarbonyl metabolites controlled for intake and excretion.

## Supplementary tables:

### Supplementary table 1
Retention times, MRM transitions, and inter batch variances.

### Supplementary table 2
Available phenotypic traits in KORA.

### Supplementary table 3
Results of phenotypic association study on dicarbonyls.

### Supplementary table 4
Meta-analysis of lead traits**.**

### Supplementary table 5
Stepwise conditional analysis.

### Supplementary table 6
Association analyses of dicarbonyls with lead SNPs of GWA-studies on BMI, glucose, GFR, GGT, and systolic blood pressure.

# Supplementary figures:

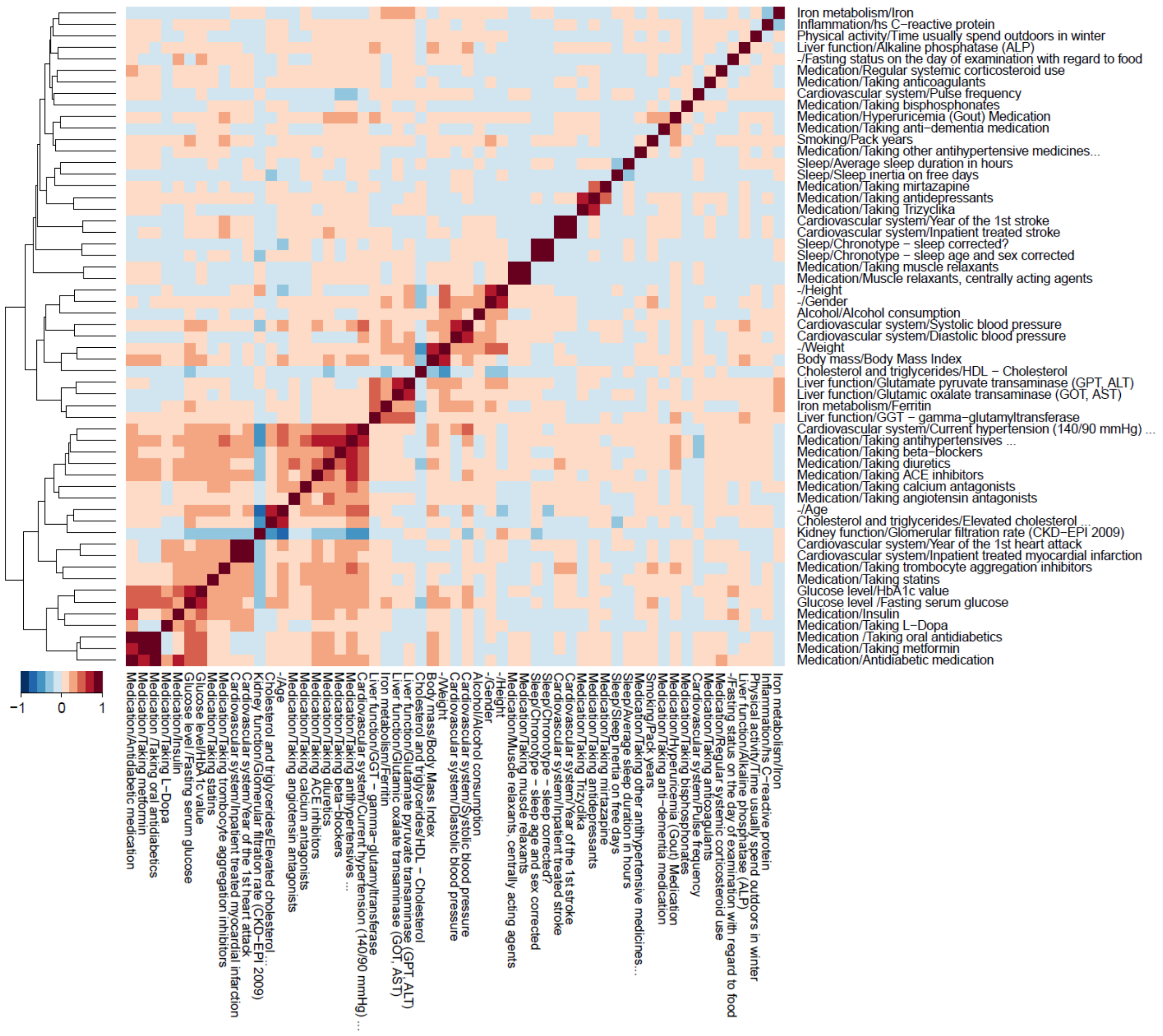


**Supplementary figure 1: Hierarchical clustering of dicarbonyl-associated traits.**
To illustrate the correlation structure of the dicarbonyl-associated traits, hierarchical clustering of the binary and continuous traits was performed using Murtagh and Legendre's criterion, and Pearson's correlation-based distance. The heatmap displays Pearson's correlation coefficients. Expectedly, this clustering matched some of our categories (e.g., "glucose level"), while some traits clustered across categories according to their physiological (e.g., "iron metabolism/iron" with "inflammation/C-reactive protein") or pharmacological relations (e.g., "cardiovascular system/hypertension" with "medication/taking antihypertensives").

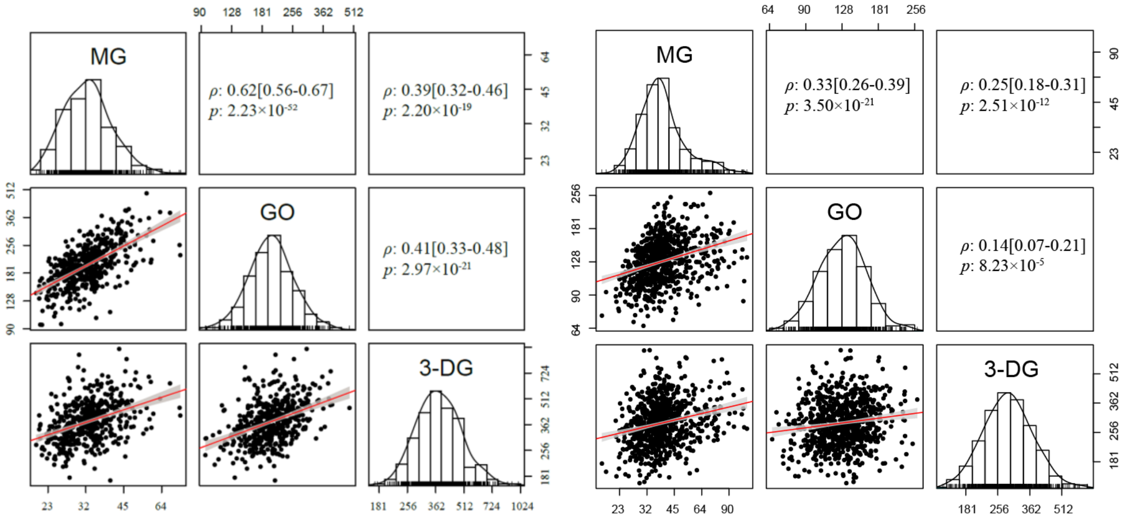


**Supplementary figure 2: Correlation plots of MG, GO, and 3-DG.**

Correlation plots of the three metabolites in KORA (right panel) and BiDirect (left panel). The frequency distributions of the concentrations [nM] of methylglyoxal (MG), glyoxal (MG), and 3-deoxyglucosone (3DG), as displayed on the diagonal, were approximately lognormal (appearing Gaussian due to logarithmic scaling). The lower left triangle displays the scatter plots with regression lines (red) and 95% confidence intervals (gray shades). The upper triangle shows the Pearson correlation coefficients with [95% confidence intervals] and correlation significance.

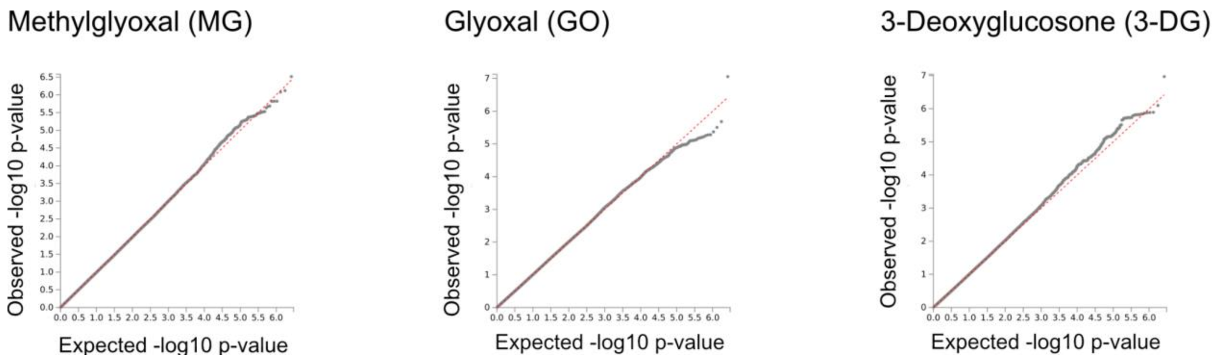


**Supplementary figure 3: QQ-plots for GWAS of dicarbonyl metabolites controlled for intake and excretion.**

QQ-plots of the GWAS of the different dicarbonyl metabolites controlled by BMI, age, sex, batch, PCA, GFR and glucose show no evidence of population stratification. They reflect the limited power of the study due to the small sample size.

# Supplementary tables:

## Supplementary table 1: Retention times, MRM transitions, and inter batch variances.

LC-MS/MS parameters of the measurements in KORA (*) and BiDirect (**).

| Substance | Inter batch [CV%] | Retention time [min] | Precursor ion [m/z] | Fragment ion (quantifier/qualifier) [m/z] |
|---|---|---|---|---|
| **3-Deoxyglucosone** | 25 (*) | 5·9 | 239·13 | 161·1 |
| | 21 (**) | | | 149·1 |
| **Glyoxal** | 15 (*) | 6·9 | 135·09 | 81·06 |
| | 15 (**) | | | 108·07 |
| **Methylglyoxal** | 12 (*) | 7·4 | 149·1 | 81·06 |
| | 12 (**) | | | 122·09 |

**Supplementary table 2: Available phenotypic traits in KORA.**
136 potentially relevant phenotypes were selected initially for the phenotypic association study in KORA.

| **Available phenotypic traits (KORA)** | |
|---|---|
| Age at diagnosis of validated diabetes mellitus with type indication | Iron |
| Age Group | Kidney disease within last 12 month |
| Alcohol consumption | LDL - cholesterol |
| Alkaline phosphatase (ALP) | L-Dopa |
| Amount of cancers | Lithium |
| Angiotensin antagonists | Liver disease within last 12 month |
| Anticoagulants | Low potency neuroleptics |
| Anti-dementia medication | Medication taken in the last 7 days |
| Antidepressants | Metformin |
| Antidiabetic medication | Mirtazapine |
| Antiepileptic drugs | Muscle relaxants |
| Antihypertensives (Assignment according to recommendations of the German League) | Muscle relaxants, centrally acting agents |
| Anxiety or panic disorders within last 12 month | Neurological diseases (e.g. multiple sclerosis or Parkinson's disease) within the last 12 month |
| Atypical neuroleptics | Non-selective monoamine reuptake inhibitors |
| Average sleep duration in hours | Non-steroidal anti-inflammatory drugs |
| Benzodiazepines | Opioids (Analgesics ) |
| Beta-blockers | Oral antidiabetics |
| Bisphosphonates | Oral contraceptives |
| Body Mass Index | Other antihypertensives (except diuretics, beta-blokers, ACE inhibitors, calcium and angiotensin antagonists) |
| Body Mass Index Categories according to WHO | Pack years |
| Calcium antagonists | Parkinson screening: Feeling that the feet are stuck to the floor (self-reported) |
| Chronotyp - sleep corrected | Parkinson screening: Has anyone told you that you have Parkinson's disease (self-reported) |
| Chronotyp - sleep, age and sex corrected | Parkinson screening: Less facial expression (self-reported) |
| Classification of work or main occupation | Parkinson screening: Problems getting up from a chair (self-reported) |
| Coronary heart disease | Parkinson screening: Quieter voice (self-reported) |
| Current hypertension (140/90 mmHg) or known hypertension controlled by medication (ISH-WHO 1999) | Parkinson screening: Smaller handwriting (self-reported) |
| Current physical condition (self-reported) | Parkinson screening: Trembling of arms and legs (self-reported) |
| Dementia screening: Difficult to plan or organise everyday life, e.g. shopping, hobbies, invitations or trips (self-reported) | Parkinson screening: Unsteady walking (self-reported) |
| Dementia screening: Difficulties following programmes on television/radio or content of newspaper/articles/books (self-reported) | Passive smoker |
| Dementia screening: Difficulties with your memory, e.g. remembering events of the day or content of conversations (self-reported) | Peripheral atherosclerosis |
| Dementia screening: Gradual start of symptoms (self-reported) | Phlebitis in superficial veins within last 12 months |
| Dementia screening: Problems carrying out familiar activities (self-reported) | Physical Activity in Categories |
| Depression(Depression module of the Brief Patient Health Questionnaire) | Previous heart operations |
| Diagnosed cancer | Proton pump inhibitors |
| Diagnosed hyperthyroidism | Pulse frequency |
| Diagnosed hypothyroidism | Regular systemic corticosteroid use |
| Diagnosed hypothyroidism | Selective monoamine reuptake inhibitors |
| Diagnosed nerve damage (neuropathy, polyneuropathy or diabetic foot) within last 12 months | Selective serotonin reuptake inhibitors |
| Diastolic blood pressure | Sex |
| Diuretics | Sleep inertia on free days |
| Dopamine agonists | Sleep latency on free days |

| | |
|---|---|
| Duration of cycling on weekdays (self-reported) | Smoking status |
| Elevated cholesterol or triglyceride levels within last 12 month | Statins |
| Examination date | Strong painkillers |
| Fasting serum glucose | Success rates of diets (self-reported) |
| Fasting status on the day of examination with regard to food | Systemic hormone therapy |
| Ferritin | Systolic blood pressure |
| Fibrates | Taking ACE inhibitors |
| Gamma-glutamyltransferase (GGT) | Thrombosis of the deep veins within last 12 month |
| Gastric or duodenal ulcer within last 12 month | Thyroid hormones |
| Gastritis within last 12 month | Thyrostatic drugs |
| Generalized Anxiety Disorder Scale | Time usually spend cycling on weekdays (self-reported) |
| Glomerular filtration rate (CKD-EPI 2009) | Time usually spend outdoors in summer (self-reported) |
| Glutamate pyruvate transaminase (GPT, ALT) | Time usually spend outdoors in winter (self-reported) |
| Glutamate receptor antagonists | Time usually spend walking on weekdays (self-reported) |
| Glutamic oxalate transaminase (GOT, AST) | Total - cholesterol |
| Gout, elevated uric acid levels within last 12 month | Total heart attacks |
| HbA1c value | Triglyceride-lowering medication |
| HDL - cholesterol | Triglycerides |
| Height | Trizyclika |
| High-potency neuroleptics | Trombocyte aggregation inhibitors |
| hs C-reactive protein | Validated diabetes mellitus with type indication |
| Hypertension classification (ISH-WHO 1999) | Waist hip ratio |
| Hyperuricemia medication | Weight |
| Inflammatory bowel disease within last 12 month | Weight loss diet within last 12 months |
| Inpatient treated myocardial infarction (self-reported) | Year of the 1st heart attack |
| Inpatient treated stroke (self-reported) | Year of the 1st stroke |
| Insulin | Zopiclone/zolpidem |

**Supplementary table 3: Results of phenotypic association study on dicarbonyls (Supplement to Figure 1).**

136 potentially relevant phenotypes were selected initially for the PheWAS in KORA. 84 of these phenotypes passed the false discovery rate (FDR) threshold of 5% for at least one dicarbonyl compound and were therefore included in further analyses. Each phenotype was assigned to one of 21 categories ("Groups") according to general physiological and medical classifications. Aligned phenotypes (23 identical traits, 21 proxies) were available in the replication set (BiDirect) for 44 of the 84 phenotypes. In both cohorts the statistics were calculated for each dicarbonyl (MG, GO, 3-DG) seperately as well as for all 3 dicarbonyls combined.

To optimize the discovery of (marginal) correlations between dicarbonyls and traits, continuous variables were transformed such as to maximize the explained variance (R2), i.e., log2-transformed ("log"), subjected to rank-based inverse normal transformation ("irn") or left untransformed ("raw"). Linear and multivariate linear models were used for regression of traits on single and multiple dicarbonyls, respectively, to calculate significance levels. The assumptions of the regression models were assessed by Shapiro–Wilk normality test of the residuals and the Cook–Weisburg test for heteroskedasticity. One round of outlier filtering was performed excluding residual outliers (pBonferroni<0·05) and data points with a Cook's distance >4/N. If the regression assumptions were not met, the transformation type was adapted or, if the latter was unsuccessful, the significance of the Fisher-transformed Spearman rank correlation coefficient was determined. Similarly, the correlation with traits represented by binary, ordinal or nominal variables were analyzed without covariates by simple, ordinal, or multinomial logistic regression models, respectively. If the regression did not converge or outlier diagnostics were still not passed after the first round of outlier filtering, Kruskal-Wallis test was used instead. R2 and Nagelkerke's pseudo-R2 were reported for linear and logistic models, respectively.

| Group | Type | Detailed phenotype (KORA) | Aligned phenotype (BiDirect) | MG (KORA) | | | | | MG (BiDirect) | | | | |
|---|---|---|---|---|---|---|---|---|---|---|---|---|---|
| | | | | Transformation | R2 | Transformation p-value | p-value | N | Transformation | R2 | Transformation p-value | p-value | N |
| Age | categorical | Age Group | Age Group | irn_cat | 4·91E-02 | irn_cat | 1·03E-04 | 483 | log_cat | 1·02E-02 | log_cat | 5·93E-02 | 796 |
| Baseline | binary | Sex | Sex | irn_cat | 4·45E-02 | irn_cat | 3·25E-05 | 483 | irn_cat | 3·19E-03 | irn_cat | 1·64E-01 | 810 |
| Physical activity | ordinal | Success rates of diets (self-reported) | NA | irn_cat | 2·64E-02 | irn_cat | 9·15E-02 | 483 | NA | NA | NA | NA | NA |
| Cardiovascular system | categorical | Coronary heart disease | Diagnosed myocardial infarction | irn_cat | 2·52E-02 | irn_cat | 2·99E-02 | 483 | irn_cat | 1·02E-02 | irn_cat | 1·70E-01 | 809 |
| Physical activity | ordinal | Current physical condition (self-reported) | NA | log_cat | 1·40E-02 | log_cat | 1·24E-01 | 483 | NA | NA | NA | NA | NA |
| Cardiovascular system | ordinal | Total heart attacks | Diagnosed 1st -3rd myocardial infarction | irn_cat | 4·16E-02 | irn_cat | 6·48E-03 | 483 | irn_cat | 1·02E-02 | irn_cat | 1·70E-01 | 809 |
| Cardiovascular system | continuous | Year of the 1st heart attack | Diagnosed myocardial infarction | irn_cat | 3·77E-02 | irn_cat | 5·69E-03 | 479 | irn_cat | 1·02E-02 | irn_cat | 1·70E-01 | 809 |
| Cardiovascular system | binary | Year of the 1st stroke | Diagnosed stroke | irn_cat | 2·05E-02 | irn_cat | 7·09E-02 | 480 | irn_cat | 9·13E-04 | irn_cat | 6·47E-01 | 809 |
| Cancer | categorical | Diagnosed cancer | Diagnosed cancer | irn_cat | 1·87E-02 | irn_cat | 7·08E-02 | 483 | irn_cat | 4·50E-04 | irn_cat | 6·60E-01 | 809 |
| Cancer | categorical | Amount of cancers | Diagnosed 1st -3rd cancer | irn_cat | 1·35E-02 | irn_cat | 1·27E-01 | 482 | irn_cat | 4·50E-04 | irn_cat | 6·60E-01 | 809 |
| Cholesterol and triglycerides | continuous | Elevated cholesterol or triglyceride levels within last 12 month | NA | raw_irn | 8·03E-03 | raw_irn | 2·93E-01 | 137 | NA | NA | NA | NA | NA |
| Cardiovascular system | categorical | Previous heart operations | Diagnosed myocardial infarction | irn_cat | 3·76E-02 | irn_cat | 2·17E-02 | 483 | irn_cat | 1·02E-02 | irn_cat | 1·70E-01 | 809 |
| Physical activity | categorical | Duration of cycling on weekdays (self-reported) | NA | irn_cat | 1·81E-02 | irn_cat | 6·33E-02 | 483 | NA | NA | NA | NA | NA |
| Baseline | categorical | Examination date | NA | log_cat | 8·54E-03 | log_cat | 1·78E-01 | 483 | NA | NA | NA | NA | NA |
| Inflammation | continuous | hs C-reactive protein | hs C-reactive protein | log_log | 2·85E-02 | irn_irn | 5·08E-04 | 482 | irn_log | 4·54E-03 | Spearman | 2·42E-02 | 781 |
| Iron metabolism | continuous | Iron | NA | log_log | 3·83E-03 | irn_irn | 1·85E-01 | 483 | NA | NA | NA | NA | NA |
| Iron metabolism | continuous | Ferritin | NA | log_log | 1·82E-02 | irn_irn | 1·53E-03 | 483 | NA | NA | NA | NA | NA |
| Liver function | continuous | Alkaline phosphatase (ALP) | NA | raw_irn | 1·91E-03 | irn_irn | 5·72E-01 | 483 | NA | NA | NA | NA | NA |
| Liver function | continuous | Gamma-glutamyltransferase (GGT) | Gamma-glutamyltransferase (GGT) | log_log | 3·43E-02 | irn_irn | 1·79E-06 | 483 | irn_log | 3·02E-04 | irn_irn | 8·12E-01 | 790 |

| | | | | | | | | | | | | | |
|---|---|---|---|---|---|---|---|---|---|---|---|---|---|
| Liver function | continuous | Glutamic oxalate transaminase (GOT, AST) | Glutamic oxalate transaminase (GOT, AST) | raw_irn | 3·08E-02 | irn_irn | 3·97E-05 | 483 | irn_log | 3·87E-03 | irn_irn | 5·78E-02 | 789 |
| Liver function | continuous | Glutamate pyruvate transaminase (GPT, ALT) | Glutamate pyruvate transaminase (GPT, ALT) | raw_irn | 1·34E-02 | irn_irn | 9·27E-03 | 483 | log_log | 2·71E-02 | irn_irn | 4·76E-05 | 788 |
| Glucose level | continuous | HbA1c value | HbA1c value | log_log | 1·29E-02 | irn_irn | 2·37E-02 | 483 | log_log | 1·39E-03 | irn_irn | 6·33E-01 | 798 |
| Cholesterol and triglycerides | continuous | HDL - cholesterol | HDL - cholesterol | log_log | 1·24E-02 | log_log | 1·40E-02 | 483 | irn_log | 3·43E-03 | irn_log | 1·00E-01 | 787 |
| Neurodegenerative disorders and neuropathies | categorical | Diagnosed nerve damage (neuropathy, polyneuropathy or diabetic foot) within last 12 months | NA | log_cat | 2·15E-03 | log_cat | 7·48E-01 | 212 | NA | NA | NA | NA | NA |
| Inflammation | ordinal | Phlebitis in superficial veins within last 12 months | NA | log_cat | 3·33E-03 | log_cat | 8·33E-01 | 382 | NA | NA | NA | NA | NA |
| Kidney function | ordinal | Kidney disease within last 12 month | Glomerular filtration rate (CKD-EPI 2009) | log_cat | 4·58E-02 | log_cat | 3·50E-02 | 381 | irn_log | 2·53E-02 | Spearman | 7·97E-05 | 788 |
| Gout and hyperuricemia | ordinal | Gout, elevated uric acid levels within last 12 month | NA | irn_cat | 3·02E-02 | irn_cat | 3·67E-02 | 382 | NA | NA | NA | NA | NA |
| Anxiety | ordinal | Anxiety or panic disorders within last 12 month | NA | log_cat | 4·23E-02 | log_cat | 1·71E-02 | 381 | NA | NA | NA | NA | NA |
| Neurodegenerative disorders and neuropathies | categorical | Parkinson screening: Unsteady walking (self-reported) | NA | log_cat | 6·54E-03 | log_cat | 7·75E-01 | 99 | NA | NA | NA | NA | NA |
| Neurodegenerative disorders and neuropathies | ordinal | Parkinson screening: Feeling that the feet are stuck to the floor (self-reported) | NA | irn_cat | 5·53E-02 | irn_cat | 8·25E-02 | 98 | NA | NA | NA | NA | NA |
| Alcohol | continuous | Alcohol consumption | Average alcohol consumption (last week) | raw_irn | 3·39E-03 | Spearman | 1·98E-01 | 483 | irn_log | 4·29E-03 | Spearman | 1·07E-01 | 741 |
| Medication | binary | Antihypertensives (Assignment according to recommendations of the German League) | NA | log_cat | 6·44E-02 | log_cat | 1·82E-06 | 483 | NA | NA | NA | NA | NA |
| Body mass | continuous | Body Mass Index | Body Mass Index | raw_irn | 8·02E-03 | irn_irn | 2·39E-02 | 480 | irn_raw | 5·05E-04 | irn_irn | 6·18E-01 | 792 |
| Body mass | ordinal | Body Mass Index Categories according to WHO | Body Mass Index | irn_cat | 8·51E-03 | irn_cat | 4·86E-01 | 480 | irn_raw | 5·05E-04 | irn_irn | 6·18E-01 | 792 |
| Cardiovascular system | continuous | Diastolic blood pressure | Diastolic blood pressure | raw_irn | 3·56E-03 | raw_irn | 1·89E-01 | 483 | log_log | 2·37E-03 | log_log | 1·66E-01 | 809 |
| Diabetes | categorical | Validated diabetes mellitus with type indication | Diagnosed diabetes mellitus | irn_cat | 3·82E-02 | irn_cat | 1·92E-03 | 483 | irn_cat | 2·23E-03 | irn_cat | 4·11E-01 | 809 |
| Diabetes | ordinal | Age at diagnosis of validated diabetes mellitus with type indication | Diagnosed diabetes mellitus | irn_cat | 7·71E-02 | irn_cat | 4·94E-02 | 483 | irn_cat | 2·23E-03 | irn_cat | 4·11E-01 | 809 |
| Anxiety | ordinal | Generalized Anxiety Disorder Scale | NA | log_cat | 4·49E-03 | log_cat | 7·00E-01 | 380 | NA | NA | NA | NA | NA |
| Baseline | continuous | Weight | Weight | raw_irn | 1·11E-02 | irn_irn | 1·46E-02 | 480 | irn_raw | 3·53E-03 | irn_log | 9·32E-02 | 808 |
| Kidney function | continuous | Glomerular filtration rate (CKD-EPI 2009) | Glomerular filtration rate (CKD-EPI 2009) | log_log | 2·40E-01 | Spearman | 4·84E-15 | 482 | irn_log | 2·53E-02 | Spearman | 7·97E-05 | 788 |
| Glucose level | continuous | Fasting serum glucose | Fasting serum glucose | raw_irn | 1·94E-02 | irn_irn | 8·09E-04 | 475 | log_log | 1·85E-03 | irn_irn | 6·00E-01 | 787 |
| Baseline | continuous | Height | Height | raw_irn | 2·30E-03 | irn_irn | 3·28E-01 | 482 | irn_log | 6·58E-03 | irn_log | 2·07E-02 | 810 |
| Medication | categorical | Systemic hormone therapy | NA | irn_cat | 4·88E-02 | irn_cat | 3·05E-05 | 483 | NA | NA | NA | NA | NA |
| Cardiovascular system | binary | Current hypertension (140/90 mmHg) or known hypertension controlled by medication (ISH-WHO 1999) | Systolic blood pressure | irn_cat | 9·17E-02 | irn_cat | 5·34E-09 | 483 | irn_raw | 1·11E-03 | irn_irn | 4·30E-01 | 809 |

| | | | | | | | | | | | | | |
|---|---|---|---|---|---|---|---|---|---|---|---|---|---|
| Medication | binary | Taking ACE inhibitors | NA | irn_cat | 2·03E-02 | irn_cat | 5·18E-03 | 483 | NA | NA | NA | NA | NA |
| Medication | binary | Insulin | Drugs used in diabetes | irn_cat | 3·78E-02 | irn_cat | 1·74E-02 | 483 | log_cat | 2·42E-02 | log_cat | 5·24E-02 | 810 |
| Medication | binary | Anticoagulants | NA | irn_cat | 1·03E-02 | irn_cat | 1·86E-01 | 483 | NA | NA | NA | NA | NA |
| Medication | binary | Trombocyte aggregation inhibitors | | irn_cat | 2·01E-02 | irn_cat | 9·49E-03 | 483 | irn_cat | 3·14E-03 | irn_cat | 4·47E-01 | 810 |
| Medication | binary | Angiotensin antagonists | NA | irn_cat | 1·27E-02 | irn_cat | 6·27E-02 | 483 | NA | NA | NA | NA | NA |
| Medication | binary | Beta-blockers | NA | log_cat | 4·42E-02 | log_cat | 1·93E-04 | 483 | NA | NA | NA | NA | NA |
| Medication | binary | Bisphosphonates | NA | log_cat | 4·39E-03 | log_cat | 5·20E-01 | 483 | NA | NA | NA | NA | NA |
| Medication | binary | Calcium antagonists | NA | irn_cat | 3·01E-03 | irn_cat | 3·09E-01 | 483 | NA | NA | NA | NA | NA |
| Medication | binary | Anti-dementia medication | NA | irn_cat | 1·73E-01 | irn_cat | 9·29E-03 | 483 | NA | NA | NA | NA | NA |
| Medication | binary | Antidepressants | Antidepressants | irn_cat | 5·27E-03 | irn_cat | 1·91E-01 | 483 | log_cat | 7·79E-03 | log_cat | 2·62E-01 | 810 |
| Medication | binary | Diuretics | NA | irn_cat | 3·41E-02 | irn_cat | 2·00E-04 | 483 | NA | NA | NA | NA | NA |
| Medication | binary | Antidiabetic medication | Drugs used in diabetes | irn_cat | 2·88E-02 | irn_cat | 6·13E-03 | 483 | log_cat | 2·42E-02 | log_cat | 5·24E-02 | 810 |
| Medication | binary | Hyperuricemia medication | NA | irn_cat | 1·25E-01 | irn_cat | 3·27E-07 | 483 | NA | NA | NA | NA | NA |
| Medication | binary | Other antihypertensives (except diuretics, beta-blokers, ACE inhibitors, calcium and angiotensin antagonists) | NA | irn_cat | 3·55E-02 | irn_cat | 5·32E-02 | 483 | NA | NA | NA | NA | NA |
| Cardiovascular system | binary | Inpatient treated myocardial infarction (self-reported) | Diagnosed myocardial infarction | irn_cat | 3·36E-02 | irn_cat | 1·21E-02 | 483 | irn_cat | 1·02E-02 | irn_cat | 1·70E-01 | 809 |
| Medication | binary | L-Dopa | Dopa and dopa derivates | irn_cat | 8·53E-04 | irn_cat | 8·02E-01 | 483 | irn_cat | 2·87E-04 | irn_cat | 9·06E-01 | 810 |
| Medication | binary | Muscle relaxants, centrally acting agents | NA | irn_cat | 4·22E-04 | irn_cat | 8·50E-01 | 483 | NA | NA | NA | NA | NA |
| Medication | binary | Metformin | Drugs used in diabetes | irn_cat | 2·15E-03 | irn_cat | 4·18E-01 | 483 | log_cat | 2·42E-02 | log_cat | 5·24E-02 | 810 |
| Medication | binary | Mirtazapine | Other antidepressants | irn_cat | 2·85E-03 | irn_cat | 5·80E-01 | 483 | log_cat | 7·79E-03 | log_cat | 2·62E-01 | 810 |
| Medication | binary | Oral antidiabetics | Drugs used in diabetes | irn_cat | 1·06E-02 | irn_cat | 1·04E-01 | 483 | log_cat | 2·42E-02 | log_cat | 5·24E-02 | 810 |
| Medication | binary | Muscle relaxants | NA | irn_cat | 4·22E-04 | irn_cat | 8·50E-01 | 483 | NA | NA | NA | NA | NA |
| Medication | binary | Statins | NA | irn_cat | 9·65E-03 | irn_cat | 6·27E-02 | 483 | NA | NA | NA | NA | NA |
| Medication | binary | Regular systemic corticosteroid use | NA | irn_cat | 1·74E-02 | irn_cat | 1·53E-01 | 483 | NA | NA | NA | NA | NA |
| Medication | binary | Trizyclika | Other antidepressants | irn_cat | 1·06E-02 | irn_cat | 1·71E-01 | 483 | log_cat | 7·79E-03 | log_cat | 2·62E-01 | 810 |
| Baseline | binary | Fasting status on the day of examination with regard to food | NA | log_cat | 4·88E-03 | log_cat | 6·79E-01 | 483 | NA | NA | NA | NA | NA |
| Medication | categorical | Oral contraceptives | NA | irn_cat | 6·55E-02 | irn_cat | 1·25E-06 | 483 | NA | NA | NA | NA | NA |
| Cardiovascular system | continuous | Pulse frequency | NA | log_log | 5·75E-03 | irn_irn | 1·25E-01 | 483 | NA | NA | NA | NA | NA |
| Smoking | continuous | Pack years | Pack years | log_log | 1·76E-02 | Spearman | 1·22E-02 | 473 | log_raw | 1·11E-03 | irn_irn | 8·57E-02 | 456 |
| Smoking | ordinal | Passive smoker | NA | irn_cat | 2·55E-02 | irn_cat | 4·56E-02 | 380 | NA | NA | NA | NA | NA |
| Cardiovascular system | binary | Inpatient treated stroke (self-reported) | NA | irn_cat | 2·10E-02 | irn_cat | 6·45E-02 | 483 | NA | NA | NA | NA | NA |
| Sleep | continuous | Average sleep duration in hours | PSQI sleep quality index | raw_irn | 9·96E-05 | raw_irn | 8·93E-01 | 181 | irn_raw | 1·93E-03 | Spearman | 4·20E-01 | 794 |
| Sleep | continuous | Sleep inertia on free days | PSQI sleep quality index | raw_log | 1·10E-02 | irn_irn | 2·94E-01 | 178 | irn_raw | 1·93E-03 | Spearman | 4·20E-01 | 794 |
| Sleep | continuous | Chronotyp – sleep, age and sex corrected | NA | raw_irn | 1·56E-02 | raw_irn | 9·18E-02 | 181 | NA | NA | NA | NA | NA |
| Sleep | continuous | Chronotyp - sleep corrected | NA | raw_irn | 1·42E-02 | raw_irn | 1·07E-01 | 181 | NA | NA | NA | NA | NA |
| Smoking | ordinal | Smoking status | Smoking status | log_cat | 2·50E-02 | log_cat | 2·29E-02 | 483 | irn_cat | 4·61E-03 | irn_cat | 1·96E-01 | 806 |
| Cardiovascular system | continuous | Systolic blood pressure | Systolic blood pressure | raw_log | 3·36E-02 | Spearman | 3·23E-03 | 483 | irn_raw | 1·11E-03 | irn_irn | 4·30E-01 | 809 |

| | | | | | | | | | | | | | |
|---|---|---|---|---|---|---|---|---|---|---|---|---|---|
| Cardiovascular system | ordinal | Hypertension classification (ISH-WHO 1999) | Systolic blood pressure | log_cat | 5·31E-02 | log_cat | 1·72E-04 | 483 | irn_raw | 1·11E-03 | irn_irn | 4·30E-01 | 809 |
| Body mass | continuous | Waist hip ratio | BMI | raw_irn | 4·46E-02 | raw_irn | 2·78E-06 | 481 | irn_raw | 5·05E-04 | irn_irn | 6·18E-01 | 792 |
| Hypothyroidism | ordinal | Diagnosed hypothyroidism | NA | log_cat | 1·91E-02 | log_cat | 5·89E-02 | 483 | NA | NA | NA | NA | NA |
| Physical activity | continuous | Time usually spend outdoors in winter (self-reported) | NA | log_log | 6·62E-05 | Spearman | 3·61E-01 | 483 | NA | NA | NA | NA | NA |

| Group | Type | Detailed phenotype (KORA) | Aligned phenotype (BiDirect) | GO (KORA) | | | | | GO (BiDirect) | | | | |
|---|---|---|---|---|---|---|---|---|---|---|---|---|---|
| | | | | Transformation | R2 | Transformation p-value | p-value | N | Transformation | R2 | Transformation p-value | p-value | N |
| Age | categorical | Age Group | Age Group | irn_cat | 1·82E-01 | irn_cat | 3·23E-19 | 483 | irn_cat | 4·19E-02 | irn_cat | 8·63E-07 | 796 |
| Baseline | binary | Sex | Sex | irn_cat | 8·77E-02 | irn_cat | 6·14E-09 | 483 | irn_cat | 5·67E-02 | irn_cat | 3·00E-09 | 810 |
| Physical activity | ordinal | Success rates of diets (self-reported) | NA | log_cat | 2·16E-02 | log_cat | 1·64E-01 | 483 | NA | NA | NA | NA | NA |
| Cardiovascular system | categorical | Coronary heart disease | Diagnosed myocardial infarction | irn_cat | 1·02E-01 | irn_cat | 1·07E-06 | 483 | irn_cat | 2·54E-02 | irn_cat | 3·04E-02 | 809 |
| Physical activity | ordinal | Current physical condition (self-reported) | NA | irn_cat | 2·79E-02 | irn_cat | 5·86E-03 | 483 | NA | NA | NA | NA | NA |
| Cardiovascular system | ordinal | Total heart attacks | Diagnosed 1st -3rd myocardial infarction | irn_cat | 1·19E-01 | irn_cat | 5·71E-07 | 483 | irn_cat | 2·54E-02 | irn_cat | 3·04E-02 | 809 |
| Cardiovascular system | continuous | Year of the 1st heart attack | Diagnosed myocardial infarction | irn_cat | 1·16E-01 | irn_cat | 6·56E-07 | 479 | irn_cat | 2·54E-02 | irn_cat | 3·04E-02 | 809 |
| Cardiovascular system | binary | Year of the 1st stroke | Diagnosed stroke | irn_cat | 5·01E-02 | irn_cat | 5·59E-03 | 480 | irn_cat | 2·50E-02 | irn_cat | 1·66E-02 | 809 |
| Cancer | categorical | Diagnosed cancer | Diagnosed cancer | irn_cat | 2·62E-02 | irn_cat | 1·72E-02 | 483 | irn_cat | 2·13E-03 | irn_cat | 3·37E-01 | 809 |
| Cancer | categorical | Amount of cancers | Diagnosed 1st -3rd cancer | irn_cat | 2·75E-02 | irn_cat | 1·45E-02 | 482 | irn_cat | 2·13E-03 | irn_cat | 3·37E-01 | 809 |
| Cholesterol and triglycerides | continuous | Elevated cholesterol or triglyceride levels within last 12 month | NA | raw_log | 4·64E-02 | raw_log | 1·07E-02 | 137 | NA | NA | NA | NA | NA |
| Cardiovascular system | categorical | Previous heart operations | Diagnosed myocardial infarction | irn_cat | 1·30E-01 | irn_cat | 6·81E-06 | 483 | irn_cat | 2·54E-02 | irn_cat | 3·04E-02 | 809 |
| Physical activity | categorical | Duration of cycling on weekdays (self-reported) | NA | log_cat | 2·73E-02 | log_cat | 1·28E-02 | 483 | NA | NA | NA | NA | NA |
| Baseline | categorical | Examination date | NA | irn_cat | 2·33E-04 | irn_cat | 8·40E-01 | 483 | NA | NA | NA | NA | NA |
| Inflammation | continuous | hs C-reactive protein | hs C-reactive protein | log_log | 3·78E-02 | irn_irn | 2·99E-05 | 482 | irn_log | 3·15E-02 | Spearman | 7·52E-07 | 781 |
| Iron metabolism | continuous | Iron | NA | log_log | 6·90E-03 | irn_irn | 1·61E-01 | 483 | NA | NA | NA | NA | NA |
| Iron metabolism | continuous | Ferritin | NA | log_log | 9·25E-02 | irn_irn | 2·60E-11 | 483 | NA | NA | NA | NA | NA |
| Liver function | continuous | Alkaline phosphatase (ALP) | NA | raw_log | 3·43E-02 | irn_irn | 5·30E-05 | 483 | NA | NA | NA | NA | NA |
| Liver function | continuous | Gamma-glutamyltransferase (GGT) | Gamma-glutamyltransferase (GGT) | log_log | 9·11E-02 | irn_irn | 2·76E-13 | 483 | irn_log | 5·29E-02 | irn_irn | 2·25E-11 | 790 |
| Liver function | continuous | Glutamic oxalate transaminase (GOT, AST) | Glutamic oxalate transaminase (GOT, AST) | log_log | 4·49E-02 | irn_irn | 6·08E-07 | 483 | irn_log | 6·11E-02 | irn_irn | 3·92E-13 | 789 |
| Liver function | continuous | Glutamate pyruvate transaminase (GPT, ALT) | Glutamate pyruvate transaminase (GPT, ALT) | log_log | 1·85E-02 | irn_irn | 1·18E-03 | 483 | irn_log | 4·62E-02 | irn_irn | 1·41E-09 | 788 |
| Glucose level | continuous | HbA1c value | HbA1c value | log_log | 5·79E-02 | irn_irn | 1·93E-10 | 483 | log_log | 1·72E-02 | irn_irn | 1·82E-04 | 798 |
| Cholesterol and triglycerides | continuous | HDL - cholesterol | HDL - cholesterol | raw_irn | 5·92E-02 | irn_irn | 5·95E-09 | 483 | irn_log | 3·03E-02 | irn_log | 8·60E-07 | 787 |

| | | | | | | | | | | | | | |
|---|---|---|---|---|---|---|---|---|---|---|---|---|---|
| Neurodegenerative disorders and neuropathies | categorical | Diagnosed nerve damage (neuropathy, polyneuropathy or diabetic foot) within last 12 months | NA | log_cat | 1·13E-03 | log_cat | 8·16E-01 | 212 | NA | NA | NA | NA | NA |
| Inflammation | ordinal | Phlebitis in superficial veins within last 12 months | NA | log_cat | 6·41E-03 | log_cat | 7·03E-01 | 382 | NA | NA | NA | NA | NA |
| Kidney function | ordinal | Kidney disease within last 12 month | Glomerular filtration rate (CKD-EPI 2009) | irn_cat | 1·95E-02 | irn_cat | 2·28E-01 | 381 | log_log | 1·06E-01 | irn_irn | 2·59E-18 | 788 |
| Gout and hyperuricemia | ordinal | Gout, elevated uric acid levels within last 12 month | NA | log_cat | 6·98E-02 | log_cat | 7·51E-04 | 382 | NA | NA | NA | NA | NA |
| Anxiety | ordinal | Anxiety or panic disorders within last 12 month | NA | log_cat | 1·25E-02 | log_cat | 3·04E-01 | 381 | NA | NA | NA | NA | NA |
| Neurodegenerative disorders and neuropathies | categorical | Parkinson screening: Unsteady walking (self-reported) | NA | irn_cat | 1·86E-02 | irn_cat | 3·83E-01 | 99 | NA | NA | NA | NA | NA |
| Neurodegenerative disorders and neuropathies | ordinal | Parkinson screening: Feeling that the feet are stuck to the floor (self-reported) | NA | irn_cat | 5·13E-02 | irn_cat | 9·12E-02 | 98 | NA | NA | NA | NA | NA |
| Alcohol | continuous | Alcohol consumption | Average alcohol consumption (last week) | raw_irn | 1·68E-02 | Spearman | 2·24E-02 | 483 | irn_raw | 8·35E-03 | Spearman | 1·50E-02 | 741 |
| Medication | binary | Antihypertensives (Assignment according to recommendations of the German League) | NA | log_cat | 1·74E-01 | log_cat | 1·12E-15 | 483 | NA | NA | NA | NA | NA |
| Body mass | continuous | Body Mass Index | Body Mass Index | raw_irn | 6·70E-02 | irn_irn | 6·44E-10 | 480 | irn_raw | 6·18E-02 | irn_log | 1·69E-12 | 792 |
| Body mass | ordinal | Body Mass Index Categories according to WHO | Body Mass Index | irn_cat | 5·72E-02 | irn_cat | 2·17E-05 | 480 | irn_raw | 6·18E-02 | irn_log | 1·69E-12 | 792 |
| Cardiovascular system | continuous | Diastolic blood pressure | Diastolic blood pressure | raw_irn | 6·62E-03 | Spearman | 7·91E-02 | 483 | irn_log | 2·97E-02 | irn_log | 7·71E-07 | 809 |
| Diabetes | categorical | Validated diabetes mellitus with type indication | Diagnosed diabetes mellitus | irn_cat | 5·04E-02 | irn_cat | 1·03E-03 | 483 | irn_cat | 3·53E-02 | irn_cat | 1·02E-03 | 809 |
| Diabetes | ordinal | Age at diagnosis of validated diabetes mellitus with type indication | Diagnosed diabetes mellitus | irn_cat | 9·40E-02 | irn_cat | 1·21E-02 | 483 | irn_cat | 3·53E-02 | irn_cat | 1·02E-03 | 809 |
| Anxiety | ordinal | Generalized Anxiety Disorder Scale | NA | log_cat | 2·72E-03 | log_cat | 8·35E-01 | 380 | NA | NA | NA | NA | NA |
| Baseline | continuous | Weight | Weight | raw_irn | 5·23E-02 | irn_irn | 1·20E-07 | 480 | irn_log | 7·43E-02 | irn_log | 2·83E-15 | 808 |
| Kidney function | continuous | Glomerular filtration rate (CKD-EPI 2009) | Glomerular filtration rate (CKD-EPI 2009) | log_log | 3·39E-01 | Spearman | 6·05E-28 | 482 | log_log | 1·06E-01 | irn_irn | 2·59E-18 | 788 |
| Glucose level | continuous | Fasting serum glucose | Fasting serum glucose | log_log | 9·27E-02 | irn_irn | 1·62E-15 | 475 | log_log | 3·44E-02 | irn_irn | 4·59E-07 | 787 |
| Baseline | continuous | Height | Height | raw_irn | 2·70E-04 | irn_irn | 8·26E-01 | 482 | irn_log | 1·68E-02 | irn_log | 2·16E-04 | 810 |
| Medication | categorical | Systemic hormone therapy | NA | irn_cat | 8·20E-02 | irn_cat | 4·00E-08 | 483 | NA | NA | NA | NA | NA |
| Cardiovascular system | binary | Current hypertension (140/90 mmHg) or known hypertension controlled by medication (ISH-WHO 1999) | Systolic blood pressure | log_cat | 2·58E-01 | log_cat | 7·52E-24 | 483 | irn_log | 3·85E-02 | irn_irn | 1·30E-08 | 809 |
| Medication | binary | Taking ACE inhibitors | NA | irn_cat | 7·35E-02 | irn_cat | 3·47E-07 | 483 | NA | NA | NA | NA | NA |
| Medication | binary | Insulin | Drugs used in diabetes | irn_cat | 5·25E-02 | irn_cat | 5·18E-03 | 483 | log_cat | 1·32E-02 | log_cat | 1·52E-01 | 810 |
| Medication | binary | Anticoagulants | NA | irn_cat | 4·75E-02 | irn_cat | 3·51E-03 | 483 | NA | NA | NA | NA | NA |
| Medication | binary | Trombocyte aggregation inhibitors | | irn_cat | 7·61E-02 | irn_cat | 7·10E-07 | 483 | log_cat | 3·25E-03 | log_cat | 4·39E-01 | 810 |
| Medication | binary | Angiotensin antagonists | NA | irn_cat | 4·64E-02 | irn_cat | 8·65E-04 | 483 | NA | NA | NA | NA | NA |
| Medication | binary | Beta-blockers | NA | log_cat | 1·32E-01 | log_cat | 6·48E-11 | 483 | NA | NA | NA | NA | NA |
| Medication | binary | Bisphosphonates | NA | irn_cat | 2·46E-03 | irn_cat | 6·11E-01 | 483 | NA | NA | NA | NA | NA |

| | | | | | | | | | | | | | |
|---|---|---|---|---|---|---|---|---|---|---|---|---|---|
| Medication | binary | Calcium antagonists | NA | irn_cat | 6·06E-02 | irn_cat | 8·71E-05 | 483 | NA | NA | NA | NA | NA |
| Medication | binary | Anti-dementia medication | NA | irn_cat | 5·00E-02 | irn_cat | 8·31E-02 | 483 | NA | NA | NA | NA | NA |
| Medication | binary | Antidepressants | Antidepressants | irn_cat | 3·74E-03 | irn_cat | 4·59E-01 | 483 | log_cat | 1·44E-03 | log_cat | 6·29E-01 | 810 |
| Medication | binary | Diuretics | NA | irn_cat | 1·39E-01 | irn_cat | 2·41E-12 | 483 | NA | NA | NA | NA | NA |
| Medication | binary | Antidiabetic medication | Drugs used in diabetes | irn_cat | 3·85E-02 | irn_cat | 2·91E-03 | 483 | log_cat | 1·32E-02 | log_cat | 1·52E-01 | 810 |
| Medication | binary | Hyperuricemia medication | NA | irn_cat | 1·92E-01 | irn_cat | 5·42E-09 | 483 | NA | NA | NA | NA | NA |
| Medication | binary | Other antihypertensives (except diuretics, beta-blokers, ACE inhibitors, calcium and angiotensin antagonists) | NA | irn_cat | 4·56E-02 | irn_cat | 3·56E-02 | 483 | NA | NA | NA | NA | NA |
| Cardiovascular system | binary | Inpatient treated myocardial infarction (self-reported) | Diagnosed myocardial infarction | irn_cat | 1·02E-01 | irn_cat | 6·16E-06 | 483 | irn_cat | 2·54E-02 | irn_cat | 3·04E-02 | 809 |
| Medication | binary | L-Dopa | Dopa and dopa derivates | irn_cat | 3·84E-02 | irn_cat | 2·02E-01 | 483 | irn_cat | 8·31E-03 | irn_cat | 5·24E-01 | 810 |
| Medication | binary | Muscle relaxants, centrally acting agents | NA | irn_cat | 2·07E-02 | irn_cat | 2·85E-01 | 483 | NA | NA | NA | NA | NA |
| Medication | binary | Metformin | Drugs used in diabetes | irn_cat | 5·40E-03 | irn_cat | 3·49E-01 | 483 | log_cat | 1·32E-02 | log_cat | 1·52E-01 | 810 |
| Medication | binary | Mirtazapine | Other antidepressants | irn_cat | 3·92E-03 | irn_cat | 6·07E-01 | 483 | log_cat | 1·44E-03 | log_cat | 6·29E-01 | 810 |
| Medication | binary | Oral antidiabetics | Drugs used in diabetes | irn_cat | 1·82E-02 | irn_cat | 6·03E-02 | 483 | log_cat | 1·32E-02 | log_cat | 1·52E-01 | 810 |
| Medication | binary | Muscle relaxants | NA | irn_cat | 2·07E-02 | irn_cat | 2·85E-01 | 483 | NA | NA | NA | NA | NA |
| Medication | binary | Statins | NA | irn_cat | 5·18E-02 | irn_cat | 6·69E-05 | 483 | NA | NA | NA | NA | NA |
| Medication | binary | Regular systemic corticosteroid use | NA | irn_cat | 6·51E-03 | irn_cat | 4·61E-01 | 483 | NA | NA | NA | NA | NA |
| Medication | binary | Trizyclika | Other antidepressants | irn_cat | 9·81E-03 | irn_cat | 3·23E-01 | 483 | log_cat | 1·44E-03 | log_cat | 6·29E-01 | 810 |
| Baseline | binary | Fasting status on the day of examination with regard to food | NA | log_cat | 2·76E-02 | log_cat | 3·25E-01 | 483 | NA | NA | NA | NA | NA |
| Medication | categorical | Oral contraceptives | NA | irn_cat | 8·15E-02 | irn_cat | 3·44E-08 | 483 | NA | NA | NA | NA | NA |
| Cardiovascular system | continuous | Pulse frequency | NA | log_log | 1·62E-02 | Spearman | 1·30E-02 | 483 | NA | NA | NA | NA | NA |
| Smoking | continuous | Pack years | Pack years | raw_log | 1·63E-02 | Spearman | 2·92E-01 | 473 | log_raw | 1·17E-03 | irn_irn | 6·02E-02 | 456 |
| Smoking | ordinal | Passive smoker | NA | log_cat | 8·96E-03 | log_cat | 5·15E-01 | 380 | NA | NA | NA | NA | NA |
| Cardiovascular system | binary | Inpatient treated stroke (self-reported) | NA | irn_cat | 4·78E-02 | irn_cat | 7·04E-03 | 483 | NA | NA | NA | NA | NA |
| Sleep | continuous | Average sleep duration in hours | PSQI sleep quality index | raw_irn | 2·15E-03 | raw_irn | 5·32E-01 | 181 | log_raw | 2·56E-04 | Spearman | 8·55E-01 | 794 |
| Sleep | continuous | Sleep inertia on free days | PSQI sleep quality index | log_log | 1·30E-02 | log_log | 1·26E-01 | 178 | log_raw | 2·56E-04 | Spearman | 8·55E-01 | 794 |
| Sleep | continuous | Chronotyp – sleep, age and sex corrected | NA | log_log | 9·66E-03 | Spearman | 6·38E-01 | 181 | NA | NA | NA | NA | NA |
| Sleep | continuous | Chronotyp - sleep corrected | NA | log_log | 3·48E-03 | Spearman | 6·96E-01 | 181 | NA | NA | NA | NA | NA |
| Smoking | ordinal | Smoking status | Smoking status | log_cat | 3·04E-02 | log_cat | 7·71E-03 | 483 | irn_cat | 1·34E-02 | irn_cat | 8·62E-03 | 806 |
| Cardiovascular system | continuous | Systolic blood pressure | Systolic blood pressure | raw_log | 8·65E-02 | Spearman | 8·59E-09 | 483 | irn_log | 3·85E-02 | irn_irn | 1·30E-08 | 809 |
| Cardiovascular system | ordinal | Hypertension classification (ISH-WHO 1999) | Systolic blood pressure | log_cat | 9·45E-02 | log_cat | 1·76E-08 | 483 | irn_log | 3·85E-02 | irn_irn | 1·30E-08 | 809 |
| Body mass | continuous | Waist hip ratio | BMI | raw_irn | 1·51E-01 | raw_irn | 6·59E-19 | 481 | irn_raw | 6·18E-02 | irn_log | 1·69E-12 | 792 |
| Hypothyroidism | ordinal | Diagnosed hypothyroidism | NA | irn_cat | 1·50E-02 | irn_cat | 1·15E-01 | 483 | NA | NA | NA | NA | NA |
| Physical activity | continuous | Time usually spend outdoors in winter (self-reported) | NA | log_log | 7·67E-03 | Spearman | 5·01E-02 | 483 | NA | NA | NA | NA | NA |

| Group | Type | Detailed phenotype (KORA) | Aligned phenotype (BiDirect) | 3-DG (KORA) | | | | | 3-DG (BiDirect) | | | | |
|---|---|---|---|---|---|---|---|---|---|---|---|---|---|
| | | | | Transformation | R2 | Transformation p-value | p-value | N | Transformation | R2 | Transformation p-value | p-value | N |
| Age | categorical | Age Group | Age Group | log_cat | 4·52E-02 | log_cat | 6·09E-04 | 483 | log_cat | 1·64E-02 | log_cat | 7·31E-03 | 796 |
| Baseline | binary | Sex | Sex | irn_cat | 3·27E-02 | irn_cat | 4·99E-04 | 483 | log_cat | 3·64E-03 | log_cat | 1·37E-01 | 810 |
| Physical activity | ordinal | Success rates of diets (self-reported) | NA | irn_cat | 4·44E-02 | irn_cat | 8·44E-03 | 483 | NA | NA | NA | NA | NA |
| Cardiovascular system | categorical | Coronary heart disease | Diagnosed myocardial infarction | log_cat | 5·85E-02 | log_cat | 1·39E-03 | 483 | log_cat | 9·56E-02 | log_cat | 2·43E-05 | 809 |
| Physical activity | ordinal | Current physical condition (self-reported) | NA | log_cat | 1·06E-02 | log_cat | 2·27E-01 | 483 | NA | NA | NA | NA | NA |
| Cardiovascular system | ordinal | Total heart attacks | Diagnosed 1st -3rd myocardial infarction | log_cat | 1·36E-01 | log_cat | 2·98E-06 | 483 | log_cat | 9·56E-02 | log_cat | 2·43E-05 | 809 |
| Cardiovascular system | continuous | Year of the 1st heart attack | Diagnosed myocardial infarction | log_cat | 8·79E-02 | log_cat | 2·67E-04 | 479 | log_cat | 9·56E-02 | log_cat | 2·43E-05 | 809 |
| Cardiovascular system | binary | Year of the 1st stroke | Diagnosed stroke | irn_cat | 6·94E-03 | irn_cat | 3·68E-01 | 480 | log_cat | 3·48E-02 | log_cat | 4·64E-03 | 809 |
| Cancer | categorical | Diagnosed cancer | Diagnosed cancer | log_cat | 4·63E-03 | log_cat | 5·81E-01 | 483 | irn_cat | 3·05E-03 | irn_cat | 2·51E-01 | 809 |
| Cancer | categorical | Amount of cancers | Diagnosed 1st -3rd cancer | log_cat | 3·80E-03 | log_cat | 6·50E-01 | 482 | irn_cat | 3·05E-03 | irn_cat | 2·51E-01 | 809 |
| Cholesterol and triglycerides | continuous | Elevated cholesterol or triglyceride levels within last 12 month | NA | raw_irn | 1·45E-02 | raw_irn | 1·57E-01 | 137 | NA | NA | NA | NA | NA |
| Cardiovascular system | categorical | Previous heart operations | Diagnosed myocardial infarction | log_cat | 1·41E-01 | log_cat | 1·78E-04 | 483 | log_cat | 9·56E-02 | log_cat | 2·43E-05 | 809 |
| Physical activity | categorical | Duration of cycling on weekdays (self-reported) | NA | irn_cat | 5·96E-03 | irn_cat | 5·65E-01 | 483 | NA | NA | NA | NA | NA |
| Baseline | categorical | Examination date | NA | log_cat | 2·83E-03 | log_cat | 5·65E-01 | 483 | NA | NA | NA | NA | NA |
| Inflammation | continuous | hs C-reactive protein | hs C-reactive protein | raw_irn | 2·13E-03 | Spearman | 6·94E-01 | 482 | log_raw | 1·69E-03 | Spearman | 8·31E-01 | 781 |
| Iron metabolism | continuous | Iron | NA | raw_log | 9·43E-04 | irn_irn | 5·61E-01 | 483 | NA | NA | NA | NA | NA |
| Iron metabolism | continuous | Ferritin | NA | log_log | 6·33E-03 | irn_irn | 9·42E-02 | 483 | NA | NA | NA | NA | NA |
| Liver function | continuous | Alkaline phosphatase (ALP) | NA | raw_irn | 5·39E-03 | irn_irn | 1·54E-01 | 483 | NA | NA | NA | NA | NA |
| Liver function | continuous | Gamma-glutamyltransferase (GGT) | Gamma-glutamyltransferase (GGT) | log_log | 3·58E-02 | irn_irn | 1·17E-04 | 483 | log_log | 7·10E-03 | irn_irn | 5·19E-02 | 790 |
| Liver function | continuous | Glutamic oxalate transaminase (GOT, AST) | Glutamic oxalate transaminase (GOT, AST) | raw_irn | 7·34E-03 | Spearman | 1·16E-01 | 483 | irn_log | 6·47E-04 | irn_irn | 4·96E-01 | 789 |
| Liver function | continuous | Glutamate pyruvate transaminase (GPT, ALT) | Glutamate pyruvate transaminase (GPT, ALT) | raw_log | 2·52E-03 | irn_irn | 5·69E-01 | 483 | log_raw | 1·52E-03 | irn_irn | 7·64E-01 | 788 |
| Glucose level | continuous | HbA1c value | HbA1c value | raw_log | 1·43E-01 | Spearman | 1·87E-09 | 483 | log_raw | 1·17E-01 | irn_irn | 4·19E-12 | 798 |
| Cholesterol and triglycerides | continuous | HDL - cholesterol | HDL - cholesterol | log_log | 1·32E-02 | log_log | 1·11E-02 | 483 | log_log | 1·64E-03 | irn_log | 3·83E-01 | 787 |
| Neurodegenerative disorders and neuropathies | categorical | Diagnosed nerve damage (neuropathy, polyneuropathy or diabetic foot) within last 12 months | NA | irn_cat | 6·00E-02 | irn_cat | 7·46E-02 | 212 | NA | NA | NA | NA | NA |
| Inflammation | ordinal | Phlebitis in superficial veins within last 12 months | NA | log_cat | 1·86E-02 | log_cat | 3·59E-01 | 382 | NA | NA | NA | NA | NA |
| Kidney function | ordinal | Kidney disease within last 12 month | Glomerular filtration rate (CKD-EPI 2009) | log_cat | 3·21E-02 | log_cat | 9·62E-02 | 381 | irn_log | 4·28E-03 | irn_irn | 5·87E-02 | 788 |
| Gout and hyperuricemia | ordinal | Gout, elevated uric acid levels within last 12 month | NA | irn_cat | 2·05E-02 | irn_cat | 1·15E-01 | 382 | NA | NA | NA | NA | NA |

| | | | | | | | | | | | | | | |
|---|---|---|---|---|---|---|---|---|---|---|---|---|---|---|
| Anxiety | ordinal | Anxiety or panic disorders within last 12 month | NA | log_cat | 3·31E-03 | log_cat | 7·30E-01 | 381 | NA | NA | NA | NA | NA |
| Neurodegenerative disorders and neuropathies | categorical | Parkinson screening: Unsteady walking (self-reported) | NA | log_cat | 1·23E-01 | Kruskal_Wallis | 4·95E-02 | 99 | NA | NA | NA | NA | NA |
| Neurodegenerative disorders and neuropathies | ordinal | Parkinson screening: Feeling that the feet are stuck to the floor (self-reported) | NA | irn_cat | 1·72E-01 | Kruskal_Wallis | 1·64E-01 | 98 | NA | NA | NA | NA | NA |
| Alcohol | continuous | Alcohol consumption | Average alcohol consumption (last week) | raw_irn | 9·75E-03 | Spearman | 4·15E-02 | 483 | irn_raw | 5·98E-03 | Spearman | 5·59E-02 | 741 |
| Medication | binary | Antihypertensives (Assignment according to recommendations of the German League) | NA | log_cat | 5·53E-02 | log_cat | 1·01E-05 | 483 | NA | NA | NA | NA | NA |
| Body mass | continuous | Body Mass Index | Body Mass Index | raw_irn | 1·52E-03 | irn_irn | 3·66E-01 | 480 | log_log | 3·96E-03 | irn_irn | 1·59E-01 | 792 |
| Body mass | ordinal | Body Mass Index Categories according to WHO | Body Mass Index | irn_cat | 7·64E-03 | irn_cat | 5·99E-01 | 480 | log_log | 3·96E-03 | irn_irn | 1·59E-01 | 792 |
| Cardiovascular system | continuous | Diastolic blood pressure | Diastolic blood pressure | raw_irn | 1·80E-04 | Spearman | 5·11E-01 | 483 | log_raw | 1·24E-04 | log_raw | 7·51E-01 | 809 |
| Diabetes | categorical | Validated diabetes mellitus with type indication | Diagnosed diabetes mellitus | log_cat | 2·62E-01 | log_cat | 3·06E-14 | 483 | log_cat | 9·49E-02 | log_cat | 6·10E-08 | 809 |
| Diabetes | ordinal | Age at diagnosis of validated diabetes mellitus with type indication | Diagnosed diabetes mellitus | irn_cat | 2·72E-01 | irn_cat | 1·40E-10 | 483 | log_cat | 9·49E-02 | log_cat | 6·10E-08 | 809 |
| Anxiety | ordinal | Generalized Anxiety Disorder Scale | NA | log_cat | 2·17E-02 | log_cat | 7·38E-02 | 380 | NA | NA | NA | NA | NA |
| Baseline | continuous | Weight | Weight | raw_irn | 1·55E-04 | irn_irn | 7·46E-01 | 480 | log_raw | 3·64E-03 | irn_log | 1·86E-01 | 808 |
| Kidney function | continuous | Glomerular filtration rate (CKD-EPI 2009) | Glomerular filtration rate (CKD-EPI 2009) | log_log | 7·37E-02 | Spearman | 5·12E-07 | 482 | irn_log | 4·28E-03 | irn_irn | 5·87E-02 | 788 |
| Glucose level | continuous | Fasting serum glucose | Fasting serum glucose | raw_log | 1·54E-01 | Spearman | 3·19E-12 | 475 | log_raw | 1·34E-01 | Spearman | 4·22E-18 | 787 |
| Baseline | continuous | Height | Height | raw_log | 1·03E-03 | irn_irn | 4·90E-01 | 482 | log_log | 7·05E-05 | log_log | 8·11E-01 | 810 |
| Medication | categorical | Systemic hormone therapy | NA | log_cat | 3·30E-02 | log_cat | 1·45E-03 | 483 | NA | NA | NA | NA | NA |
| Cardiovascular system | binary | Current hypertension (140/90 mmHg) or known hypertension controlled by medication (ISH-WHO 1999) | Systolic blood pressure | log_cat | 8·40E-02 | log_cat | 3·01E-08 | 483 | log_raw | 1·74E-03 | irn_irn | 4·59E-01 | 809 |
| Medication | binary | Taking ACE inhibitors | NA | log_cat | 4·99E-02 | log_cat | 1·82E-04 | 483 | NA | NA | NA | NA | NA |
| Medication | binary | Insulin | Drugs used in diabetes | irn_cat | 2·69E-01 | irn_cat | 4·31E-08 | 483 | log_cat | 1·55E-01 | log_cat | 7·59E-07 | 810 |
| Medication | binary | Anticoagulants | NA | log_cat | 2·23E-02 | log_cat | 8·09E-02 | 483 | NA | NA | NA | NA | NA |
| Medication | binary | Trombocyte aggregation inhibitors | | log_cat | 3·11E-02 | log_cat | 3·13E-03 | 483 | irn_cat | 4·32E-03 | irn_cat | 3·72E-01 | 810 |
| Medication | binary | Angiotensin antagonists | NA | irn_cat | 4·03E-02 | irn_cat | 7·35E-03 | 483 | NA | NA | NA | NA | NA |
| Medication | binary | Beta-blockers | NA | log_cat | 2·45E-02 | log_cat | 5·64E-03 | 483 | NA | NA | NA | NA | NA |
| Medication | binary | Bisphosphonates | NA | log_cat | 5·59E-02 | log_cat | 2·13E-02 | 483 | NA | NA | NA | NA | NA |
| Medication | binary | Calcium antagonists | NA | log_cat | 5·77E-02 | log_cat | 4·96E-04 | 483 | NA | NA | NA | NA | NA |
| Medication | binary | Anti-dementia medication | NA | log_cat | 1·33E-04 | log_cat | 9·45E-01 | 483 | NA | NA | NA | NA | NA |
| Medication | binary | Antidepressants | Antidepressants | log_cat | 1·38E-02 | log_cat | 1·56E-01 | 483 | log_cat | 4·59E-03 | log_cat | 3·89E-01 | 810 |
| Medication | binary | Diuretics | NA | log_cat | 4·12E-02 | log_cat | 5·18E-04 | 483 | NA | NA | NA | NA | NA |
| Medication | binary | Antidiabetic medication | Drugs used in diabetes | log_cat | 2·19E-01 | log_cat | 3·01E-11 | 483 | log_cat | 1·55E-01 | log_cat | 7·59E-07 | 810 |
| Medication | binary | Hyperuricemia medication | NA | log_cat | 7·57E-02 | log_cat | 1·24E-03 | 483 | NA | NA | NA | NA | NA |

| | | | | | | | | | | | | | |
|---|---|---|---|---|---|---|---|---|---|---|---|---|---|
| Medication | binary | Other antihypertensives (except diuretics, beta-blokers, ACE inhibitors, calcium and angiotensin antagonists) | NA | irn_cat | 9·86E-02 | irn_cat | 6·20E-03 | 483 | NA | NA | NA | NA | NA |
| Cardiovascular system | binary | Inpatient treated myocardial infarction (self-reported) | Diagnosed myocardial infarction | log_cat | 1·45E-01 | log_cat | 7·11E-06 | 483 | log_cat | 9·56E-02 | log_cat | 2·43E-05 | 809 |
| Medication | binary | L-Dopa | Dopa and dopa derivates | irn_cat | 1·46E-01 | irn_cat | 3·90E-02 | 483 | irn_cat | 9·03E-03 | irn_cat | 5·06E-01 | 810 |
| Medication | binary | Muscle relaxants, centrally acting agents | NA | irn_cat | 1·74E-01 | irn_cat | 3·13E-03 | 483 | NA | NA | NA | NA | NA |
| Medication | binary | Metformin | Drugs used in diabetes | log_cat | 8·33E-02 | log_cat | 5·68E-04 | 483 | log_cat | 1·55E-01 | log_cat | 7·59E-07 | 810 |
| Medication | binary | Mirtazapine | Other antidepressants | irn_cat | 1·27E-01 | irn_cat | 1·41E-02 | 483 | log_cat | 4·59E-03 | log_cat | 3·89E-01 | 810 |
| Medication | binary | Oral antidiabetics | Drugs used in diabetes | log_cat | 1·19E-01 | log_cat | 9·76E-06 | 483 | log_cat | 1·55E-01 | log_cat | 7·59E-07 | 810 |
| Medication | binary | Muscle relaxants | NA | irn_cat | 1·74E-01 | irn_cat | 3·13E-03 | 483 | NA | NA | NA | NA | NA |
| Medication | binary | Statins | NA | log_cat | 3·57E-02 | log_cat | 2·51E-03 | 483 | NA | NA | NA | NA | NA |
| Medication | binary | Regular systemic corticosteroid use | NA | log_cat | 1·76E-01 | log_cat | 4·96E-04 | 483 | NA | NA | NA | NA | NA |
| Medication | binary | Trizyclika | Other antidepressants | log_cat | 1·06E-04 | log_cat | 9·23E-01 | 483 | log_cat | 4·59E-03 | log_cat | 3·89E-01 | 810 |
| Baseline | binary | Fasting status on the day of examination with regard to food | NA | irn_cat | 1·34E-01 | irn_cat | 2·46E-02 | 483 | NA | NA | NA | NA | NA |
| Medication | categorical | Oral contraceptives | NA | irn_cat | 3·15E-02 | irn_cat | 1·60E-03 | 483 | NA | NA | NA | NA | NA |
| Cardiovascular system | continuous | Pulse frequency | NA | log_log | 2·49E-03 | Spearman | 5·10E-01 | 483 | NA | NA | NA | NA | NA |
| Smoking | continuous | Pack years | Pack years | raw_log | 3·90E-02 | Spearman | 6·53E-05 | 473 | log_raw | 1·19E-02 | irn_irn | 1·09E-02 | 456 |
| Smoking | ordinal | Passive smoker | NA | irn_cat | 5·37E-02 | irn_cat | 3·94E-04 | 380 | NA | NA | NA | NA | NA |
| Cardiovascular system | binary | Inpatient treated stroke (self-reported) | NA | irn_cat | 5·94E-03 | irn_cat | 4·00E-01 | 483 | NA | NA | NA | NA | NA |
| Sleep | continuous | Average sleep duration in hours | PSQI sleep quality index | log_log | 4·78E-03 | log_log | 3·52E-01 | 181 | log_log | 5·18E-04 | Spearman | 6·10E-01 | 794 |
| Sleep | continuous | Sleep inertia on free days | PSQI sleep quality index | raw_irn | 5·34E-03 | Spearman | 4·96E-01 | 178 | log_log | 5·18E-04 | Spearman | 6·10E-01 | 794 |
| Sleep | continuous | Chronotyp – sleep, age and sex corrected | NA | log_log | 9·39E-04 | irn_irn | 8·63E-01 | 181 | NA | NA | NA | NA | NA |
| Sleep | continuous | Chronotyp - sleep corrected | NA | log_log | 9·40E-05 | irn_irn | 9·76E-01 | 181 | NA | NA | NA | NA | NA |
| Smoking | ordinal | Smoking status | Smoking status | irn_cat | 3·40E-02 | irn_cat | 2·80E-03 | 483 | log_cat | 9·50E-03 | log_cat | 3·42E-02 | 806 |
| Cardiovascular system | continuous | Systolic blood pressure | Systolic blood pressure | raw_irn | 4·03E-02 | Spearman | 2·90E-05 | 483 | log_raw | 1·74E-03 | irn_irn | 4·59E-01 | 809 |
| Cardiovascular system | ordinal | Hypertension classification (ISH-WHO 1999) | Systolic blood pressure | irn_cat | 5·64E-02 | irn_cat | 4·50E-05 | 483 | log_raw | 1·74E-03 | irn_irn | 4·59E-01 | 809 |
| Body mass | continuous | Waist hip ratio | BMI | raw_irn | 3·13E-02 | raw_irn | 9·27E-05 | 481 | log_log | 3·96E-03 | irn_irn | 1·59E-01 | 792 |
| Hypothyroidism | ordinal | Diagnosed hypothyroidism | NA | log_cat | 2·70E-02 | log_cat | 1·46E-02 | 483 | NA | NA | NA | NA | NA |
| Physical activity | continuous | Time usually spend outdoors in winter (self-reported) | NA | raw_log | 3·88E-03 | Spearman | 2·12E-01 | 483 | NA | NA | NA | NA | NA |

| Group | Type | Detailed phenotype (KORA) | Aligned phenotype (BiDirect) | Combined (KORA) | | | | | Combined (BiDirect) | | | | |
|---|---|---|---|---|---|---|---|---|---|---|---|---|---|
| | | | | Transformation | R2 | Transformation p-value | p-value | N | Transformation | R2 | Transformation p-value | p-value | N |
| Age | categorical | Age Group | Age Group | log_raw | 8·34E-02 | log_raw | 4·51E-15 | 473 | log_raw | 1·35E-02 | log_raw | 2·10E-08 | 771 |
| Baseline | binary | Sex | Sex | log_raw | 3·92E-02 | log_raw | 1·75E-08 | 475 | log_raw | 1·53E-02 | log_raw | 1·33E-09 | 787 |
| Physical activity | ordinal | Success rates of diets (self-reported) | NA | log_raw | 5·68E-03 | log_raw | 3·77E-01 | 472 | NA | NA | NA | NA | NA |
| Cardiovascular system | categorical | Coronary heart disease | Diagnosed myocardial infarction | log_raw | 1·81E-02 | log_raw | 2·66E-04 | 474 | log_raw | 1·68E-03 | log_raw | 1·16E-02 | 797 |
| Physical activity | ordinal | Current physical condition (self-reported) | NA | log_raw | 1·45E-02 | log_raw | 3·56E-02 | 471 | NA | NA | NA | NA | NA |
| Cardiovascular system | ordinal | Total heart attacks | Diagnosed 1st -3rd myocardial infarction | log_raw | 1·19E-02 | log_raw | 1·18E-03 | 469 | log_raw | 1·68E-03 | log_raw | 1·16E-02 | 797 |
| Cardiovascular system | continuous | Year of the 1st heart attack | Diagnosed myocardial infarction | log_raw | 1·57E-02 | log_raw | 2·21E-04 | 464 | log_raw | 1·68E-03 | log_raw | 1·16E-02 | 797 |
| Cardiovascular system | binary | Year of the 1st stroke | Diagnosed stroke | log_raw | 1·31E-03 | log_raw | 7·43E-02 | 470 | log_raw | 1·32E-03 | log_raw | 1·32E-02 | 794 |
| Cancer | categorical | Diagnosed cancer | Diagnosed cancer | log_raw | 3·88E-03 | log_raw | 6·54E-03 | 474 | log_raw | 1·63E-03 | log_raw | 1·15E-02 | 784 |
| Cancer | categorical | Amount of cancers | Diagnosed 1st -3rd cancer | log_raw | 3·37E-03 | log_raw | 1·03E-02 | 473 | log_raw | 1·63E-03 | log_raw | 1·15E-02 | 784 |
| Cholesterol and triglycerides | continuous | Elevated cholesterol or triglyceride levels within last 12 month | NA | log_irn | 3·30E-02 | log_irn | 4·25E-03 | 133 | NA | NA | NA | NA | NA |
| Cardiovascular system | categorical | Previous heart operations | Diagnosed myocardial infarction | log_raw | 1·15E-02 | log_raw | 1·26E-03 | 474 | log_raw | 1·68E-03 | log_raw | 1·16E-02 | 797 |
| Physical activity | categorical | Duration of cycling on weekdays (self-reported) | NA | log_raw | 4·58E-03 | log_raw | 2·28E-01 | 479 | NA | NA | NA | NA | NA |
| Baseline | categorical | Examination date | NA | log_raw | -1·86E-03 | log_raw | 1·66E-01 | 477 | NA | NA | NA | NA | NA |
| Inflammation | continuous | hs C-reactive protein | hs C-reactive protein | log_raw | 2·16E-02 | log_raw | 1·16E-05 | 478 | log_irn | 1·07E-02 | log_irn | 1·02E-06 | 758 |
| Iron metabolism | continuous | Iron | NA | log_log | 1·38E-03 | log_log | 6·90E-02 | 471 | NA | NA | NA | NA | NA |
| Iron metabolism | continuous | Ferritin | NA | log_irn | 3·35E-02 | log_irn | 2·31E-08 | 473 | NA | NA | NA | NA | NA |
| Liver function | continuous | Alkaline phosphatase (ALP) | NA | log_irn | 1·06E-02 | log_irn | 7·50E-04 | 470 | NA | NA | NA | NA | NA |
| Liver function | continuous | Gamma-glutamyltransferase (GGT) | Gamma-glutamyltransferase (GGT) | log_irn | 4·62E-02 | log_irn | 3·90E-10 | 474 | log_irn | 1·66E-02 | log_irn | 2·42E-11 | 770 |
| Liver function | continuous | Glutamic oxalate transaminase (GOT, AST) | Glutamic oxalate transaminase (GOT, AST) | log_irn | 2·23E-02 | log_irn | 7·47E-06 | 473 | log_irn | 1·65E-02 | log_irn | 6·38E-11 | 776 |
| Liver function | continuous | Glutamate pyruvate transaminase (GPT, ALT) | Glutamate pyruvate transaminase (GPT, ALT) | log_log | 8·98E-03 | log_log | 2·11E-03 | 475 | log_raw | 1·74E-02 | log_raw | 2·63E-15 | 782 |
| Glucose level | continuous | HbA1c value | HbA1c value | log_irn | 5·42E-02 | log_irn | 4·66E-12 | 477 | log_log | 1·81E-02 | log_log | 7·77E-13 | 784 |
| Cholesterol and triglycerides | continuous | HDL - cholesterol | HDL - cholesterol | log_raw | 2·78E-02 | log_raw | 8·14E-07 | 475 | log_log | 1·09E-02 | log_log | 1·51E-06 | 772 |
| Neurodegenerative disorders and neuropathies | categorical | Diagnosed nerve damage (neuropathy, polyneuropathy or diabetic foot) within last 12 months | NA | log_raw | -4·51E-03 | log_raw | 5·74E-01 | 206 | NA | NA | NA | NA | NA |
| Inflammation | ordinal | Phlebitis in superficial veins within last 12 months | NA | log_raw | -3·96E-03 | log_raw | 6·98E-01 | 377 | NA | NA | NA | NA | NA |
| Kidney function | ordinal | Kidney disease within last 12 month | Glomerular filtration rate (CKD-EPI 2009) | log_raw | -1·81E-03 | log_raw | 5·98E-01 | 374 | log_irn | 3·35E-02 | log_irn | 6·23E-17 | 773 |
| Gout and hyperuricemia | ordinal | Gout, elevated uric acid levels within last 12 month | NA | log_raw | 8·58E-03 | log_raw | 3·33E-02 | 372 | NA | NA | NA | NA | NA |

| | | | | | | | | | | | | | |
|---|---|---|---|---|---|---|---|---|---|---|---|---|---|
| Anxiety | ordinal | Anxiety or panic disorders within last 12 month | NA | log_raw | -3·16E-03 | log_raw | 8·34E-01 | 369 | NA | NA | NA | NA | NA |
| Neurodegenerative disorders and neuropathies | categorical | Parkinson screening: Unsteady walking (self-reported) | NA | log_raw | 7·10E-03 | log_raw | 1·16E-01 | 95 | NA | NA | NA | NA | NA |
| Neurodegenerative disorders and neuropathies | ordinal | Parkinson screening: Feeling that the feet are stuck to the floor (self-reported) | NA | log_raw | -5·43E-04 | log_raw | 1·99E-01 | 94 | NA | NA | NA | NA | NA |
| Alcohol | continuous | Alcohol consumption | Average alcohol consumption (last week) | log_raw | 8·49E-03 | log_raw | 4·33E-03 | 477 | log_log | 5·90E-03 | log_log | 1·01E-03 | 720 |
| Medication | binary | Antihypertensives (Assignment according to recommendations of the German League) | NA | log_raw | 6·35E-02 | log_raw | 3·60E-13 | 478 | NA | NA | NA | NA | NA |
| Body mass | continuous | Body Mass Index | Body Mass Index | log_irn | 2·71E-02 | log_irn | 2·54E-07 | 471 | log_irn | 1·47E-02 | log_irn | 4·01E-11 | 781 |
| Body mass | ordinal | Body Mass Index Categories according to WHO | Body Mass Index | log_raw | 1·47E-02 | log_raw | 4·72E-04 | 474 | log_irn | 1·47E-02 | log_irn | 4·01E-11 | 781 |
| Cardiovascular system | continuous | Diastolic blood pressure | Diastolic blood pressure | log_log | 3·27E-03 | log_log | 3·54E-02 | 477 | log_raw | 7·98E-03 | log_raw | 6·02E-07 | 784 |
| Diabetes | categorical | Validated diabetes mellitus with type indication | Diagnosed diabetes mellitus | log_raw | 4·63E-02 | log_raw | 5·41E-11 | 472 | log_raw | 4·59E-03 | log_raw | 6·87E-05 | 795 |
| Diabetes | ordinal | Age at diagnosis of validated diabetes mellitus with type indication | Diagnosed diabetes mellitus | log_raw | 3·14E-02 | log_raw | 5·18E-04 | 471 | log_raw | 4·59E-03 | log_raw | 6·87E-05 | 795 |
| Anxiety | ordinal | Generalized Anxiety Disorder Scale | NA | log_raw | -3·01E-03 | log_raw | 6·72E-01 | 376 | NA | NA | NA | NA | NA |
| Baseline | continuous | Weight | Weight | log_log | 1·86E-02 | log_log | 1·57E-05 | 472 | log_log | 2·19E-02 | log_log | 2·59E-13 | 793 |
| Kidney function | continuous | Glomerular filtration rate (CKD-EPI 2009) | Glomerular filtration rate (CKD-EPI 2009) | log_log | 1·61E-01 | log_log | 6·30E-33 | 477 | log_irn | 3·35E-02 | log_irn | 6·23E-17 | 773 |
| Glucose level | continuous | Fasting serum glucose | Fasting serum glucose | log_irn | 1·00E-01 | log_irn | 1·50E-19 | 467 | log_raw | 3·73E-02 | log_raw | 9·31E-25 | 776 |
| Baseline | continuous | Height | Height | log_log | -9·18E-04 | log_log | 2·09E-01 | 476 | log_irn | 6·80E-03 | log_irn | 2·02E-04 | 793 |
| Medication | categorical | Systemic hormone therapy | NA | log_raw | 3·52E-02 | log_raw | 4·08E-06 | 474 | NA | NA | NA | NA | NA |
| Cardiovascular system | binary | Current hypertension (140/90 mmHg) or known hypertension controlled by medication (ISH-WHO 1999) | Systolic blood pressure | log_raw | 1·02E-01 | log_raw | 1·65E-20 | 479 | log_irn | 8·69E-03 | log_irn | 3·21E-07 | 792 |
| Medication | binary | Taking ACE inhibitors | NA | log_raw | 2·21E-02 | log_raw | 7·81E-06 | 470 | NA | NA | NA | NA | NA |
| Medication | binary | Insulin | Drugs used in diabetes | log_raw | 1·51E-02 | log_raw | 1·18E-04 | 474 | log_raw | 5·21E-03 | log_raw | 6·81E-06 | 800 |
| Medication | binary | Anticoagulants | NA | log_raw | 5·24E-03 | log_raw | 1·52E-02 | 472 | NA | NA | NA | NA | NA |
| Medication | binary | Trombocyte aggregation inhibitors | | log_raw | 1·90E-02 | log_raw | 2·70E-05 | 468 | log_raw | -9·34E-04 | log_raw | 4·86E-01 | 796 |
| Medication | binary | Angiotensin antagonists | NA | log_raw | 6·04E-03 | log_raw | 1·13E-02 | 474 | NA | NA | NA | NA | NA |
| Medication | binary | Beta-blockers | NA | log_raw | 3·36E-02 | log_raw | 4·01E-08 | 477 | NA | NA | NA | NA | NA |
| Medication | binary | Bisphosphonates | NA | log_raw | -9·68E-04 | log_raw | 2·41E-01 | 473 | NA | NA | NA | NA | NA |
| Medication | binary | Calcium antagonists | NA | log_raw | 1·27E-02 | log_raw | 5·09E-04 | 472 | NA | NA | NA | NA | NA |
| Medication | binary | Anti-dementia medication | NA | log_raw | 2·65E-03 | log_raw | 3·29E-02 | 483 | NA | NA | NA | NA | NA |
| Medication | binary | Antidepressants | Antidepressants | log_raw | 7·80E-03 | log_raw | 3·15E-03 | 474 | log_raw | -1·08E-03 | log_raw | 4·61E-01 | 798 |
| Medication | binary | Diuretics | NA | log_raw | 4·34E-02 | log_raw | 7·26E-10 | 473 | NA | NA | NA | NA | NA |
| Medication | binary | Antidiabetic medication | Drugs used in diabetes | log_raw | 3·73E-02 | log_raw | 4·33E-09 | 473 | log_raw | 5·21E-03 | log_raw | 6·81E-06 | 800 |
| Medication | binary | Hyperuricemia medication | NA | log_raw | 2·17E-02 | log_raw | 1·76E-05 | 471 | NA | NA | NA | NA | NA |

| | | | | | | | | | | | | | | |
|---|---|---|---|---|---|---|---|---|---|---|---|---|---|---|
| Medication | binary | Other antihypertensives (except diuretics, beta-blokers, ACE inhibitors, calcium and angiotensin antagonists) | NA | log_raw | 8·87E-04 | log_raw | 1·25E-01 | 479 | NA | NA | NA | NA | NA |
| Cardiovascular system | binary | Inpatient treated myocardial infarction (self-reported) | Diagnosed myocardial infarction | log_raw | 1·56E-02 | log_raw | 2·19E-04 | 470 | log_raw | 1·68E-03 | log_raw | 1·16E-02 | 797 |
| Medication | binary | L-Dopa | Dopa and dopa derivates | log_raw | 2·60E-03 | log_raw | 3·70E-02 | 483 | NA | NA | NA | NA | NA |
| Medication | binary | Muscle relaxants, centrally acting agents | NA | log_raw | 3·90E-03 | log_raw | 1·46E-02 | 483 | NA | NA | NA | NA | NA |
| Medication | binary | Metformin | Drugs used in diabetes | log_raw | 2·39E-03 | log_raw | 4·50E-02 | 473 | log_raw | 5·21E-03 | log_raw | 6·81E-06 | 800 |
| Medication | binary | Mirtazapine | Other antidepressants | log_raw | -1·15E-03 | log_raw | 2·63E-01 | 479 | log_raw | -1·08E-03 | log_raw | 4·61E-01 | 798 |
| Medication | binary | Oral antidiabetics | Drugs used in diabetes | log_raw | 1·35E-02 | log_raw | 3·31E-04 | 474 | log_raw | 5·21E-03 | log_raw | 6·81E-06 | 800 |
| Medication | binary | Muscle relaxants | NA | log_raw | 3·90E-03 | log_raw | 1·46E-02 | 483 | NA | NA | NA | NA | NA |
| Medication | binary | Statins | NA | log_raw | 1·38E-02 | log_raw | 3·06E-04 | 471 | NA | NA | NA | NA | NA |
| Medication | binary | Regular systemic corticosteroid use | NA | log_raw | 2·39E-03 | log_raw | 5·58E-02 | 478 | NA | NA | NA | NA | NA |
| Medication | binary | Trizyclika | Other antidepressants | log_raw | 7·00E-04 | log_raw | 1·48E-01 | 474 | log_raw | -1·08E-03 | log_raw | 4·61E-01 | 798 |
| Baseline | binary | Fasting status on the day of examination with regard to food | NA | log_raw | -1·86E-04 | log_raw | 6·33E-02 | 481 | NA | NA | NA | NA | NA |
| Medication | categorical | Oral contraceptives | NA | log_raw | 4·36E-02 | log_raw | 6·70E-10 | 473 | NA | NA | NA | NA | NA |
| Cardiovascular system | continuous | Pulse frequency | NA | log_irn | 3·70E-03 | log_irn | 2·40E-02 | 475 | NA | NA | NA | NA | NA |
| Smoking | continuous | Pack years | Pack years | log_raw | 2·02E-02 | log_raw | 3·75E-05 | 463 | log_raw | 7·72E-04 | log_raw | 3·31E-02 | 444 |
| Smoking | ordinal | Passive smoker | NA | log_raw | 2·14E-02 | log_raw | 9·29E-04 | 370 | NA | NA | NA | NA | NA |
| Cardiovascular system | binary | Inpatient treated stroke (self-reported) | NA | log_raw | 1·17E-03 | log_raw | 7·74E-02 | 473 | NA | NA | NA | NA | NA |
| Sleep | continuous | Average sleep duration in hours | PSQI sleep quality index | log_log | -1·66E-03 | log_log | 2·10E-01 | 177 | log_raw | 9·02E-04 | log_raw | 7·13E-02 | 778 |
| Sleep | continuous | Sleep inertia on free days | PSQI sleep quality index | log_log | 8·46E-03 | log_log | 3·42E-02 | 175 | log_raw | 9·02E-04 | log_raw | 7·13E-02 | 778 |
| Sleep | continuous | Chronotyp – sleep, age and sex corrected | NA | log_log | -6·91E-04 | log_log | 1·90E-01 | 174 | NA | NA | NA | NA | NA |
| Sleep | continuous | Chronotyp - sleep corrected | NA | log_raw | -2·19E-03 | log_raw | 1·66E-01 | 175 | NA | NA | NA | NA | NA |
| Smoking | ordinal | Smoking status | Smoking status | log_raw | 1·99E-02 | log_raw | 1·36E-03 | 474 | log_raw | 2·64E-03 | log_raw | 5·24E-03 | 784 |
| Cardiovascular system | continuous | Systolic blood pressure | Systolic blood pressure | log_raw | 4·22E-02 | log_raw | 4·89E-09 | 478 | log_irn | 8·69E-03 | log_irn | 3·21E-07 | 792 |
| Cardiovascular system | ordinal | Hypertension classification (ISH-WHO 1999) | Systolic blood pressure | log_raw | 3·51E-02 | log_raw | 6·45E-04 | 479 | log_irn | 8·69E-03 | log_irn | 3·21E-07 | 792 |
| Body mass | continuous | Waist hip ratio | BMI | log_log | 8·29E-02 | log_log | 1·11E-16 | 471 | log_irn | 1·47E-02 | log_irn | 4·01E-11 | 781 |
| Hypothyroidism | ordinal | Diagnosed hypothyroidism | NA | log_raw | 1·02E-02 | log_raw | 1·34E-01 | 466 | NA | NA | NA | NA | NA |
| Physical activity | continuous | Time usually spend outdoors in winter (self-reported) | NA | log_raw | 3·85E-03 | log_raw | 1·98E-02 | 480 | NA | NA | NA | NA | NA |

**Supplementary table 4: Meta-analysis of lead traits.**

A phenotype was considered to be a lead trait of a category if in KORA

i) the association of that trait reached Bonferroni-corrected significance for at least one of the 3 dicarbonyls (MG, GO, 3-DG)

and

ii) that association was more significant than the respective associations of other traits in that category.

12 of the 15 lead traits were available in both population samples (exactly or as a proxy) and could be included in the meta-analysis.

| Group | Detailed phenotype (KORA) | Aligned phenotype (BiDirect) | MG | | | | | | | | | | | | | |
|---|---|---|---|---|---|---|---|---|---|---|---|---|---|---|---|---|
| | | | KORA | | | BiDirect | | | Meta random effect | | | | | Meta fixed effect | | |
| | | | beta | SE | p-value | beta | SE | p-value | p-value_het | I2 | beta | SE | p-value | beta | SE | p-value |
| Age | Age Group | Age Group | 0·005 | 0·001 | 6·90E-06 | -0·002 | 0·002 | 1·91E-01 | 6·21E-04 | 91·461 | 0·001 | 0·004 | 7·02E-01 | 0·003 | 0·001 | 1·09E-03 |
| Baseline | Sex (female) | Sex (female) | -0·113 | 0·031 | 2·68E-04 | -0·040 | 0·030 | 1·79E-01 | 9·12E-02 | 64·943 | -0·076 | 0·036 | 3·54E-02 | -0·076 | 0·021 | 4·21E-04 |
| Body mass | BMI | BMI | 0·005 | 0·003 | 1·47E-01 | 0·001 | 0·003 | 7·02E-01 | 4·74E-01 | 0·000 | 0·003 | 0·002 | 1·87E-01 | 0·003 | 0·002 | 1·87E-01 |
| Cardiovascular system | Systolic blood pressure | Systolic blood pressure | 0·003 | 0·001 | 1·59E-04 | 0·002 | 0·001 | 1·14E-01 | 1·60E-01 | 49·242 | 0·003 | 0·001 | 6·70E-03 | 0·003 | 0·001 | 1·05E-04 |
| Cholesterol and triglycerides | HDL - cholesterol | HDL - cholesterol | -0·002 | 0·001 | 2·33E-02 | -0·614 | 0·462 | 1·84E-01 | 1·85E-01 | 42·957 | -0·134 | 0·251 | 5·94E-01 | -0·002 | 0·001 | 2·26E-02 |
| Diabetes | Validated diabetes mellitus with type indication | Diagnosed diabetes mellitus | 0·158 | 0·054 | 3·76E-03 | 0·026 | 0·064 | 6·85E-01 | 1·13E-01 | 60·133 | 0·096 | 0·066 | 1·46E-01 | 0·102 | 0·041 | 1·33E-02 |
| Glucose level | Fasting serum glucose | Serum glucose | 0·003 | 0·001 | 2·52E-03 | -0·001 | 0·001 | 1·19E-01 | 8·62E-04 | 90·993 | 0·001 | 0·002 | 7·01E-01 | 0·000 | 0·001 | 4·55E-01 |
| Inflammation | hs C-reactive protein | hs C-reactive protein | 0·015 | 0·004 | 6·18E-04 | 0·009 | 0·007 | 2·42E-01 | 4·16E-01 | 0·000 | 0·014 | 0·004 | 3·88E-04 | 0·014 | 0·004 | 3·88E-04 |
| Kidney function | Glomerular filtration rate (CKD-EPI 2009) | Glomerular filtration rate (CKD-EPI 2009) | -0·009 | 0·001 | 6·06E-22 | -0·002 | 0·001 | 1·79E-02 | 2·71E-06 | 95·457 | -0·006 | 0·003 | 7·47E-02 | -0·006 | 0·001 | 1·80E-20 |
| Liver function | Gamma-glutamyltransferase (GGT) | Gamma-glutamyltransferase (GGT) | 0·090 | 0·027 | 8·72E-04 | -0·008 | 0·026 | 7·56E-01 | 9·15E-03 | 85·279 | 0·041 | 0·049 | 4·06E-01 | 0·040 | 0·019 | 3·34E-02 |
| Medication | Antidiabetic medication | Drugs used in diabetes | 0·155 | 0·061 | 1·09E-02 | -0·214 | 0·106 | 4·48E-02 | 2·59E-03 | 88·981 | -0·019 | 0·184 | 9·17E-01 | 0·065 | 0·053 | 2·20E-01 |
| Smoking | Pack years | Pack years | 0·053 | 0·039 | 1·81E-01 | 0·066 | 0·052 | 2·09E-01 | 8·40E-01 | 0·000 | 0·057 | 0·031 | 6·78E-02 | 0·057 | 0·031 | 6·78E-02 |

| Group | Detailed phenotype (KORA) | Aligned phenotype (BiDirect) | GO | | | | | | | | | | | | | |
|---|---|---|---|---|---|---|---|---|---|---|---|---|---|---|---|---|
| | | | KORA | | | BiDirect | | | Meta random effect | | | | | Meta fixed effect | | |
| | | | beta | SE | p-value | beta | SE | p-value | p-value_het | I2 | beta | SE | p-value | beta | SE | p-value |
| Age | Age Group | Age Group | 0·011 | 0·001 | 8·29E-22 | 0·007 | 0·007 | 1·76E-07 | 5·94E-01 | 0·000 | 0·011 | 0·001 | 4·71E-24 | 0·011 | 0·001 | 4·71E-24 |
| Baseline | Sex (female) | Sex (female) | -0·194 | 0·034 | 2·86E-08 | -0·160 | 0·023 | 2·75E-12 | 4·15E-01 | 0·000 | -0·171 | 0·019 | 1·66E-19 | -0·171 | 0·019 | 1·66E-19 |
| Body mass | BMI | BMI | 0·017 | 0·004 | 3·39E-06 | 0·019 | 0·003 | 2·47E-13 | 5·88E-01 | 0·000 | 0·018 | 0·002 | 1·44E-18 | 0·018 | 0·002 | 1·44E-18 |
| Cardiovascular system | Systolic blood pressure | Systolic blood pressure | 0·006 | 0·001 | 2·15E-09 | 0·005 | 0·001 | 5·61E-10 | 2·53E-01 | 23·505 | 0·005 | 0·001 | 5·28E-14 | 0·005 | 0·001 | 3·91E-18 |
| Cholesterol and triglycerides | HDL - cholesterol | HDL - cholesterol | -0·006 | 0·001 | 3·29E-06 | -0·280 | 0·353 | 4·28E-01 | 4·37E-01 | 0·000 | -0·006 | 0·001 | 2·48E-06 | -0·006 | 0·001 | 2·48E-06 |
| Diabetes | Validated diabetes mellitus with type indication | Diagnosed diabetes mellitus | 0·203 | 0·062 | 1·04E-03 | 0·142 | 0·049 | 3·93E-03 | 4·34E-01 | 0·000 | 0·166 | 0·038 | 1·58E-05 | 0·166 | 0·038 | 1·58E-05 |
| Glucose level | Fasting serum glucose | Serum glucose | 0·005 | 0·001 | 7·19E-09 | 0·002 | 0·001 | 4·90E-06 | 5·30E-03 | 87·137 | 0·004 | 0·001 | 9·38E-03 | 0·003 | 0·000 | 3·98E-12 |
| Inflammation | hs C-reactive protein | hs C-reactive protein | 0·015 | 0·005 | 3·48E-03 | 0·028 | 0·006 | 5·85E-07 | 8·38E-02 | 66·558 | 0·021 | 0·007 | 1·08E-03 | 0·021 | 0·004 | 2·55E-08 |
| Kidney function | Glomerular filtration rate (CKD-EPI 2009) | Glomerular filtration rate (CKD-EPI 2009) | -0·012 | 0·001 | 2·53E-36 | -0·006 | 0·001 | 1·56E-16 | 3·97E-07 | 96·110 | -0·009 | 0·003 | 1·72E-03 | -0·009 | 0·001 | 1·06E-52 |
| Liver function | Gamma-glutamyltransferase (GGT) | Gamma-glutamyltransferase (GGT) | 0·118 | 0·030 | 1·12E-04 | 0·102 | 0·020 | 5·38E-07 | 6·58E-01 | 0·000 | 0·107 | 0·017 | 1·93E-10 | 0·107 | 0·017 | 1·93E-10 |
| Medication | Antidiabetic medication | Drugs used in diabetes | 0·191 | 0·069 | 5·85E-03 | 0·000 | 0·083 | 9·99E-01 | 7·59E-02 | 68·260 | 0·101 | 0·095 | 2·90E-01 | 0·113 | 0·053 | 3·30E-02 |
| Smoking | Pack years | Pack years | 0·040 | 0·045 | 3·74E-01 | -0·042 | 0·041 | 2·98E-01 | 1·74E-01 | 45·821 | -0·003 | 0·041 | 9·34E-01 | -0·005 | 0·030 | 8·64E-01 |

| Group | Detailed phenotype (KORA) | Aligned phenotype (BiDirect) | 3-DG | | | | | | | | | | | | | |
|---|---|---|---|---|---|---|---|---|---|---|---|---|---|---|---|---|
| | | | KORA | | | BiDirect | | | Meta random effect | | | | | Meta fixed effect | | |
| | | | beta | SE | p-value | beta | SE | p-value | p-value_het | I2 | beta | SE | p-value | beta | SE | p-value |
| Age | Age Group | Age Group | 0·005 | 0·001 | 1·10E-05 | 0·005 | 0·002 | 1·68E-03 | 8·84E-01 | 0·000 | 0·005 | 0·001 | 5·15E-08 | 0·005 | 0·001 | 5·15E-08 |
| Baseline | Sex (female) | Sex (female) | -0·111 | 0·036 | 1·88E-03 | -0·043 | 0·027 | 1·12E-01 | 1·29E-01 | 56·646 | -0·073 | 0·034 | 2·99E-02 | -0·068 | 0·022 | 1·57E-03 |
| Body mass | BMI | BMI | 0·002 | 0·004 | 5·84E-01 | 0·002 | 0·003 | 5·28E-01 | 9·88E-01 | 0·000 | 0·002 | 0·002 | 4·03E-01 | 0·002 | 0·002 | 4·03E-01 |
| Cardiovascular system | Systolic blood pressure | Systolic blood pressure | 0·004 | 0·001 | 4·32E-04 | 0·001 | 0·001 | 5·40E-01 | 2·20E-02 | 80·938 | 0·002 | 0·002 | 1·87E-01 | 0·002 | 0·001 | 5·54E-03 |
| Cholesterol and triglycerides | HDL - cholesterol | HDL - cholesterol | -0·003 | 0·001 | 1·09E-02 | 0·680 | 0·423 | 1·09E-01 | 1·06E-01 | 61·625 | 0·207 | 0·316 | 5·11E-01 | -0·003 | 0·001 | 1·08E-02 |
| Diabetes | Validated diabetes mellitus with type indication | Diagnosed diabetes mellitus | 0·467 | 0·060 | 2·89E-14 | 0·219 | 0·057 | 1·31E-04 | 2·71E-03 | 88·877 | 0·342 | 0·124 | 5·60E-03 | 0·338 | 0·041 | 2·37E-16 |
| Glucose level | Fasting serum glucose | Serum glucose | 0·008 | 0·001 | 1·33E-18 | 0·005 | 0·001 | 1·48E-17 | 7·36E-03 | 86·077 | 0·007 | 0·001 | 3·82E-06 | 0·006 | 0·000 | 3·03E-35 |
| Inflammation | hs C-reactive protein | hs C-reactive protein | 0·005 | 0·005 | 3·70E-01 | 0·001 | 0·007 | 8·61E-01 | 6·76E-01 | 0·000 | 0·003 | 0·004 | 4·16E-01 | 0·003 | 0·004 | 4·16E-01 |
| Kidney function | Glomerular filtration rate (CKD-EPI 2009) | Glomerular filtration rate (CKD-EPI 2009) | -0·006 | 0·001 | 1·76E-07 | -0·002 | 0·001 | 3·56E-02 | 9·76E-03 | 85·026 | -0·004 | 0·002 | 3·95E-02 | -0·004 | 0·001 | 3·67E-07 |
| Liver function | Gamma-glutamyltransferase (GGT) | Gamma-glutamyltransferase (GGT) | 0·110 | 0·031 | 3·92E-04 | 0·035 | 0·024 | 1·41E-01 | 5·56E-02 | 72·716 | 0·070 | 0·037 | 5·99E-02 | 0·064 | 0·019 | 7·95E-04 |
| Medication | Antidiabetic medication | Drugs used in diabetes | 0·470 | 0·067 | 7·84E-12 | 0·408 | 0·096 | 2·23E-05 | 5·95E-01 | 0·000 | 0·450 | 0·055 | 2·58E-16 | 0·450 | 0·055 | 2·58E-16 |
| Smoking | Pack years | Pack years | 0·187 | 0·045 | 3·27E-05 | 0·096 | 0·048 | 4·32E-02 | 1·63E-01 | 48·695 | 0·143 | 0·045 | 1·65E-03 | 0·145 | 0·033 | 8·87E-06 |

**Supplementary table 5: Stepwise conditional analysis of traits in KORA that passed the false discovery rate (FDR) threshold of 5% for at least one dicarbonyl compound (MG, GO, 3-DG).**

Stepwise conditional phenotypic association study of a dicarbonyl started with using the most significantly associated trait as a covariate in the repetition of the analyses of all other traits. The most significant trait in the repetition was then added as covariate for another round of analyses. This procedure was repeated until no further trait passed the Bonferroni-corrected significance threshold in the likelihood-ratio-test (P Lrtest≤0·05/84) anymore (of note, p-values usually do not represent the significance of an unconditioned analysis). Permutation p-values (P perm.) are calculated based on the simulated null distribution of the likelihood-ratio statistic, providing a robust reference for possible complex collinearity and data distribution in this study.

N indicates the number of traits that were conditioned on. If N=2, for instance, the analysis of the respective trait was conditioned on the trait with N=0 and the trait with N=1.

| Group | Type | Detailed phenotype (KORA) | Shapiro-Wilk | MG | | | | | GO | | | | | 3-DG | | | | |
|---|---|---|---|---|---|---|---|---|---|---|---|---|---|---|---|---|---|---|
| | | | | R2 | R2 SE | P Lrtest | P perm. | N | R2 | R2 SE | P Lrtest | P perm. | N | R2 | R2 SE | P Lrtest | P perm. | N |
| Kidney function | continous | Glomerular filtration rate (CKD-EPI 2009) | 1·94E-04 | 2·40E-01 | 7·14E-02 | 8·80E-19 | 0·00E+00 | 0 | 3·39E-01 | 6·32E-02 | 6·05E-28 | 0·00E+00 | 0 | 2·91E-02 | 1·45E-02 | 1·12E-05 | 2·00E-04 | 1 |
| Cardiovascular system | ordinal | Hypertension classification (ISH-WHO 1999) | NA | 1·82E-02 | 6·00E-03 | 1·47E-02 | 1·22E-01 | 2 | 1·34E-02 | 3·95E-03 | 1·97E-02 | 2·68E-01 | 4 | 1·78E-02 | 7·22E-03 | 5·30E-02 | 1·53E-01 | 3 |
| Cardiovascular system | binary | Current hypertension (140/90 mmHg) or known hypertension controlled by medication (ISH-WHO 1999) | NA | 1·43E-03 | 4·26E-03 | 2·96E-01 | 4·11E-01 | 2 | 1·22E-02 | 8·22E-03 | 5·57E-04 | 1·74E-02 | 3 | 4·82E-03 | 4·96E-03 | 1·13E-01 | 1·88E-01 | 3 |
| Baseline | catological | Examination date | NA | 1·53E-03 | 2·65E-03 | 5·57E-01 | 6·84E-01 | 2 | 3·82E-03 | 4·34E-03 | 1·51E-01 | 3·98E-01 | 4 | 1·71E-02 | 6·71E-03 | 1·12E-02 | 4·10E-02 | 3 |
| Age | catological | Age Group | NA | 3·08E-03 | 3·85E-03 | 7·98E-01 | 9·14E-01 | 2 | 3·07E-03 | 4·05E-03 | 6·95E-01 | 9·17E-01 | 4 | 7·66E-03 | 4·86E-03 | 4·06E-01 | 5·92E-01 | 3 |
| Glucose level | continous | HbA1c value | 4·40E-23 | 1·98E-03 | 3·04E-03 | 2·18E-01 | 3·25E-01 | 2 | 2·18E-03 | 2·80E-03 | 1·42E-01 | 3·02E-01 | 4 | 5·41E-03 | 6·88E-03 | 9·31E-02 | 1·55E-01 | 3 |
| Body mass | continous | Waist hip ratio | 1·58E-01 | 3·87E-04 | 2·69E-03 | 5·88E-01 | 6·67E-01 | 2 | 5·79E-02 | 1·51E-02 | 1·07E-12 | 0·00E+00 | 1 | 3·91E-03 | 6·58E-03 | 1·56E-01 | 2·31E-01 | 3 |
| Diabetes | catological | Validated diabetes mellitus with type indication | NA | 1·34E-03 | 2·16E-03 | 3·12E-01 | 4·04E-01 | 2 | 1·13E-03 | 1·76E-03 | 2·90E-01 | 4·61E-01 | 4 | 1·01E-02 | 4·41E-03 | 2·16E-02 | 5·56E-02 | 3 |
| Medication | binary | Antihypertensives (Assignment according to recommendations of the German League) | NA | 3·02E-04 | 2·00E-03 | 6·31E-01 | 6·94E-01 | 2 | 5·75E-03 | 3·25E-03 | 1·69E-02 | 9·84E-02 | 4 | 1·56E-04 | 1·92E-03 | 7·76E-01 | 8·17E-01 | 3 |
| Medication | binary | Antidiabetic medication | NA | 1·50E-03 | 1·88E-03 | 2·84E-01 | 3·89E-01 | 2 | 1·67E-03 | 1·89E-03 | 1·99E-01 | 3·64E-01 | 4 | 3·69E-03 | 2·76E-03 | 1·66E-01 | 2·53E-01 | 3 |
| Glucose level | continous | Fasting serum glucose | 8·46E-28 | 2·77E-04 | 1·89E-03 | 6·50E-01 | 7·15E-01 | 2 | 6·00E-05 | 1·51E-03 | 8·09E-01 | 8·69E-01 | 4 | 1·54E-01 | 3·71E-02 | 5·10E-19 | 0·00E+00 | 0 |
| Medication | binary | Insulin | NA | 4·11E-04 | 9·43E-04 | 5·75E-01 | 6·56E-01 | 2 | 1·06E-03 | 9·67E-04 | 3·07E-01 | 4·75E-01 | 4 | 1·09E-02 | 2·46E-03 | 1·72E-02 | 4·64E-02 | 3 |
| Medication | binary | Diuretics | NA | 1·89E-04 | 1·31E-03 | 7·03E-01 | 7·65E-01 | 2 | 6·82E-05 | 1·02E-03 | 7·95E-01 | 8·52E-01 | 4 | 2·64E-03 | 2·47E-03 | 2·42E-01 | 3·25E-01 | 3 |
| Cardiovascular system | continous | Year of the 1st heart attack | NA | 3·70E-04 | 1·41E-03 | 5·97E-01 | 6·69E-01 | 2 | 5·00E-04 | 1·19E-03 | 4·86E-01 | 6·13E-01 | 4 | 5·06E-04 | 7·39E-04 | 6·08E-01 | 6·63E-01 | 3 |
| Medication | binary | Beta-blockers | NA | 5·83E-04 | 2·35E-03 | 5·04E-01 | 5·93E-01 | 2 | 3·29E-04 | 1·11E-03 | 5·69E-01 | 6·92E-01 | 4 | 8·76E-03 | 6·31E-03 | 3·25E-02 | 6·66E-02 | 3 |
| Cardiovascular system | ordinal | Total heart attacks | NA | 5·82E-04 | 1·28E-03 | 8·00E-01 | 8·64E-01 | 2 | 7·67E-04 | 1·19E-03 | 6·85E-01 | 8·18E-01 | 4 | 6·06E-05 | 2·25E-04 | 8·60E-01 | 8·77E-01 | 3 |
| Cardiovascular system | binary | Inpatient treated myocardial infarction (self-reported) | NA | 6·15E-05 | 6·74E-04 | 8·28E-01 | 8·62E-01 | 2 | 9·04E-04 | 1·57E-03 | 3·45E-01 | 5·09E-01 | 4 | 4·95E-05 | 2·92E-04 | 8·73E-01 | 8·98E-01 | 3 |
| Cardiovascular system | continous | Systolic blood pressure | 2·03E-05 | 9·49E-04 | 2·09E-03 | 3·94E-01 | 4·95E-01 | 2 | 9·84E-04 | 2·96E-03 | 3·25E-01 | 4·84E-01 | 4 | 3·68E-03 | 4·08E-03 | 1·67E-01 | 2·42E-01 | 3 |
| Alcohol | continous | Alcohol consumption | 3·03E-28 | 8·81E-04 | 3·03E-03 | 4·11E-01 | 5·19E-01 | 2 | 3·47E-03 | 3·76E-03 | 6·41E-02 | 2·02E-01 | 4 | 7·10E-03 | 6·23E-03 | 5·43E-02 | 1·08E-01 | 3 |
| Cardiovascular system | continous | Diastolic blood pressure | 6·34E-01 | 1·75E-05 | 1·31E-03 | 9·08E-01 | 9·26E-01 | 2 | 6·14E-04 | 2·25E-03 | 4·37E-01 | 5·83E-01 | 4 | 1·04E-06 | 1·87E-03 | 9·81E-01 | 9·81E-01 | 3 |
| Baseline | continous | Weight | 1·73E-04 | 3·93E-04 | 1·82E-03 | 5·85E-01 | 6·68E-01 | 2 | 6·32E-04 | 2·34E-03 | 4·32E-01 | 5·85E-01 | 4 | 2·17E-02 | 1·07E-02 | 7·71E-04 | 3·80E-03 | 3 |
| Iron metabolism | continous | Iron | 2·41E-07 | 2·04E-03 | 3·77E-03 | 2·11E-01 | 3·22E-01 | 2 | 4·91E-03 | 4·63E-03 | 2·74E-02 | 1·22E-01 | 4 | 3·93E-06 | 2·28E-03 | 9·64E-01 | 9·70E-01 | 3 |
| Body mass | continous | Body Mass Index | 3·75E-09 | 2·79E-04 | 1·93E-03 | 6·46E-01 | 7·19E-01 | 2 | 2·23E-06 | 1·36E-03 | 9·63E-01 | 9·72E-01 | 4 | 1·93E-02 | 1·01E-02 | 1·54E-03 | 6·60E-03 | 3 |
| Iron metabolism | continous | Ferritin | 1·17E-29 | 2·22E-05 | 1·89E-03 | 8·96E-01 | 9·14E-01 | 2 | 5·81E-03 | 4·72E-03 | 1·64E-02 | 8·98E-02 | 4 | 1·09E-03 | 2·85E-03 | 4·52E-01 | 5·32E-01 | 3 |
| Baseline | continous | Height | 2·47E-02 | 2·49E-05 | 1·84E-03 | 8·90E-01 | 9·14E-01 | 2 | 1·19E-03 | 2·21E-03 | 2·79E-01 | 4·51E-01 | 4 | 2·74E-03 | 4·30E-03 | 2·33E-01 | 3·17E-01 | 3 |

| | | | | | | | | | | | | | | | | | | |
|---|---|---|---|---|---|---|---|---|---|---|---|---|---|---|---|---|---|---|
| Liver function | continous | Glutamate pyruvate transaminase (GPT, ALT) | 1·03E-28 | 5·90E-03 | 5·35E-03 | 3·32E-02 | 9·68E-02 | 2 | 1·32E-03 | 2·28E-03 | 2·53E-01 | 4·33E-01 | 4 | 9·41E-05 | 3·27E-03 | 8·25E-01 | 8·57E-01 | 3 |
| Medication | binary | Hyperuricemia medication | NA | 1·01E-02 | 6·37E-03 | 5·22E-03 | 3·18E-02 | 2 | 6·89E-03 | 3·67E-03 | 8·92E-03 | 6·76E-02 | 4 | 2·96E-03 | 1·72E-03 | 2·14E-01 | 2·93E-01 | 3 |
| Liver function | continous | Glutamic oxalate transaminase (GOT, AST) | 1·04E-32 | 9·29E-03 | 7·06E-03 | 7·45E-03 | 3·80E-02 | 2 | 5·01E-03 | 5·03E-03 | 2·60E-02 | 1·22E-01 | 4 | 5·70E-03 | 6·12E-03 | 8·47E-02 | 1·49E-01 | 3 |
| Inflammation | continous | hs C-reactive protein | 6·23E-40 | 4·04E-03 | 4·23E-03 | 7·87E-02 | 1·70E-01 | 2 | 5·07E-06 | 1·44E-03 | 9·44E-01 | 9·59E-01 | 4 | 9·00E-04 | 7·40E-03 | 4·94E-01 | 5·60E-01 | 3 |
| Liver function | continous | Alkaline phosphatase (ALP) | 3·56E-10 | 5·89E-04 | 2·33E-03 | 5·02E-01 | 5·99E-01 | 2 | 1·25E-03 | 2·45E-03 | 2·66E-01 | 4·32E-01 | 4 | 8·44E-04 | 3·33E-03 | 5·08E-01 | 5·77E-01 | 3 |
| Smoking | continous | Pack years | 1·73E-29 | 7·49E-03 | 5·29E-03 | 1·75E-02 | 5·22E-02 | 2 | 8·49E-04 | 2·92E-03 | 3·67E-01 | 5·25E-01 | 4 | 1·02E-02 | 7·33E-03 | 2·20E-02 | 5·40E-02 | 3 |
| Medication | catological | Systemic hormone therapy | NA | 4·38E-03 | 3·15E-03 | 6·69E-02 | 1·42E-01 | 2 | 6·50E-03 | 5·40E-03 | 3·98E-02 | 2·07E-01 | 4 | 2·51E-03 | 2·29E-03 | 5·21E-01 | 6·30E-01 | 3 |
| Medication | catological | Oral contraceptives | NA | 4·85E-02 | 1·62E-02 | 1·76E-08 | 0·00E+00 | 1 | 6·20E-03 | 5·00E-03 | 4·61E-02 | 2·24E-01 | 4 | 8·23E-04 | 2·65E-03 | 8·08E-01 | 8·53E-01 | 3 |
| Medication | binary | ACE inhibitors | NA | 1·51E-03 | 2·57E-03 | 2·82E-01 | 3·92E-01 | 2 | 3·71E-03 | 3·41E-03 | 5·55E-02 | 1·84E-01 | 4 | 5·81E-04 | 1·32E-03 | 5·83E-01 | 6·36E-01 | 3 |
| Medication | binary | Trombocyte aggregation inhibitors | NA | 2·73E-05 | 1·41E-03 | 8·85E-01 | 9·11E-01 | 2 | 8·44E-05 | 1·29E-03 | 7·73E-01 | 8·45E-01 | 4 | 7·23E-04 | 1·62E-03 | 5·40E-01 | 6·09E-01 | 3 |
| Cardiovascular system | catological | Coronary heart disease | NA | 7·36E-04 | 9·01E-04 | 7·55E-01 | 8·22E-01 | 2 | 2·52E-03 | 2·13E-03 | 2·89E-01 | 5·39E-01 | 4 | 1·06E-03 | 8·64E-04 | 7·59E-01 | 8·27E-01 | 3 |
| Medication | binary | Oral antidiabetics | NA | 8·70E-04 | 1·21E-03 | 4·15E-01 | 5·08E-01 | 2 | 4·66E-04 | 8·90E-04 | 4·98E-01 | 6·27E-01 | 4 | 7·87E-04 | 1·32E-03 | 5·23E-01 | 5·93E-01 | 3 |
| Cardiovascular system | catological | Previous heart operations | NA | 4·75E-05 | 5·35E-04 | 9·82E-01 | 9·87E-01 | 2 | 4·98E-03 | 2·33E-03 | 8·48E-02 | 2·91E-01 | 4 | 1·56E-03 | 8·81E-04 | 3·68E-01 | 4·41E-01 | 3 |
| Medication | binary | Regular systemic corticosteroid use | NA | 4·54E-04 | 4·91E-04 | 5·56E-01 | 6·27E-01 | 2 | 3·37E-06 | 7·29E-05 | 9·54E-01 | 9·61E-01 | 4 | 1·90E-02 | 2·87E-03 | 1·55E-03 | 8·40E-03 | 3 |
| Smoking | ordinal | Passive smoker | NA | 2·18E-02 | 9·19E-03 | 2·66E-02 | 7·86E-02 | 2 | 4·84E-03 | 5·13E-03 | 5·37E-01 | 7·69E-01 | 4 | 4·79E-02 | 1·69E-02 | 7·79E-05 | 1·40E-03 | 2 |
| Medication | binary | Calcium antagonists | NA | 5·95E-04 | 1·31E-03 | 5·00E-01 | 5·90E-01 | 2 | 2·18E-03 | 2·42E-03 | 1·43E-01 | 3·01E-01 | 4 | 1·18E-02 | 5·97E-03 | 1·29E-02 | 4·10E-02 | 3 |
| Medication | binary | Angiotensin antagonists | NA | 9·69E-05 | 6·04E-04 | 7·85E-01 | 8·28E-01 | 2 | 2·31E-04 | 1·19E-03 | 6·34E-01 | 7·42E-01 | 4 | 1·05E-04 | 6·21E-04 | 8·16E-01 | 8·40E-01 | 3 |
| Medication | binary | Statins | NA | 1·15E-03 | 1·97E-03 | 3·48E-01 | 4·54E-01 | 2 | 8·21E-05 | 1·13E-03 | 7·76E-01 | 8·45E-01 | 4 | 4·36E-05 | 9·16E-04 | 8·80E-01 | 9·03E-01 | 3 |
| Body mass | ordinal | Body Mass Index Categories according to WHO | NA | 1·72E-03 | 2·78E-03 | 9·34E-01 | 9·72E-01 | 2 | 8·72E-03 | 4·06E-03 | 1·27E-01 | 5·06E-01 | 4 | 2·97E-02 | 7·24E-03 | 8·21E-03 | 5·24E-02 | 3 |
| Sleep | continous | Average sleep duration in hours | 9·50E-01 | 4·08E-05 | 4·58E-03 | 9·21E-01 | 9·32E-01 | 2 | 7·36E-04 | 4·94E-03 | 6·11E-01 | 7·15E-01 | 4 | 7·00E-03 | 7·87E-03 | 1·66E-01 | 2·63E-01 | 3 |
| Medication | binary | Metformin | NA | 1·96E-04 | 6·14E-04 | 6·98E-01 | 7·59E-01 | 2 | 1·43E-03 | 1·09E-03 | 2·35E-01 | 4·00E-01 | 4 | 7·75E-05 | 5·31E-04 | 8·41E-01 | 8·71E-01 | 3 |
| Sleep | continous | Chronotyp - sleep corrected | 2·43E-01 | 2·76E-03 | 6·51E-03 | 4·12E-01 | 4·85E-01 | 2 | 5·21E-04 | 3·67E-03 | 6·69E-01 | 7·62E-01 | 4 | 5·02E-03 | 5·83E-03 | 2·41E-01 | 3·37E-01 | 3 |
| Liver function | continous | Gamma-glutamyltransferase (GGT) | 1·89E-38 | 5·11E-03 | 5·83E-03 | 4·76E-02 | 1·16E-01 | 2 | 1·99E-02 | 8·75E-03 | 1·52E-05 | 1·40E-03 | 2 | 3·05E-05 | 1·67E-03 | 9·00E-01 | 9·14E-01 | 3 |
| Gout and hyperuricemia | ordinal | Gout, elevated uric acid levels within last 12 month | NA | 6·55E-03 | 4·15E-03 | 1·95E-01 | 2·80E-01 | 2 | 6·53E-03 | 3·00E-03 | 1·22E-01 | 2·79E-01 | 4 | 7·09E-03 | 4·69E-03 | 1·57E-01 | 2·68E-01 | 3 |
| Sleep | continous | Chronotyp - sleep, age and sex corrected | 6·81E-01 | 2·07E-03 | 6·09E-03 | 4·78E-01 | 5·29E-01 | 2 | 6·66E-05 | 4·19E-03 | 8·78E-01 | 9·13E-01 | 4 | 5·47E-03 | 5·90E-03 | 2·21E-01 | 3·15E-01 | 3 |
| Anxiety | ordinal | Anxiety or panic disorders within last 12 month | NA | 1·79E-02 | 8·71E-03 | 1·13E-02 | 3·70E-02 | 2 | 9·57E-04 | 1·95E-03 | 7·37E-01 | 8·41E-01 | 4 | 1·03E-03 | 1·02E-03 | 7·66E-01 | 8·15E-01 | 3 |
| Cancer | catological | Amount of cancers | NA | 1·75E-03 | 2·08E-03 | 5·11E-01 | 6·39E-01 | 2 | 1·71E-03 | 1·66E-03 | 4·31E-01 | 6·46E-01 | 4 | 3·19E-03 | 2·27E-03 | 4·39E-01 | 5·55E-01 | 3 |
| Kidney function | ordinal | Kidney disease within last 12 month | NA | 1·15E-02 | 3·05E-03 | 5·67E-02 | 1·13E-01 | 2 | 2·20E-03 | 1·14E-03 | 4·96E-01 | 6·57E-01 | 4 | 6·26E-03 | 3·54E-03 | 1·97E-01 | 3·11E-01 | 3 |
| Cardiovascular system | continous | Pulse frequency | 1·23E-01 | 1·02E-04 | 1·70E-03 | 7·80E-01 | 8·32E-01 | 2 | 1·23E-03 | 2·15E-03 | 2·71E-01 | 4·41E-01 | 4 | 7·04E-04 | 3·36E-03 | 5·45E-01 | 6·11E-01 | 3 |
| Smoking | ordinal | Smoking status | NA | 1·22E-02 | 5·86E-03 | 5·19E-02 | 2·16E-01 | 2 | 2·63E-03 | 2·50E-03 | 6·27E-01 | 8·67E-01 | 4 | 9·84E-03 | 6·36E-03 | 7·64E-02 | 1·64E-01 | 3 |
| Anxiety | ordinal | Generalized Anxiety Disorder Scale | NA | 7·78E-03 | 3·88E-03 | 2·79E-01 | 3·95E-01 | 2 | 9·25E-04 | 2·28E-03 | 9·00E-01 | 9·46E-01 | 4 | 1·67E-02 | 3·44E-03 | 3·39E-02 | 9·60E-02 | 3 |
| Medication | binary | L-Dopa | NA | 2·69E-04 | 2·15E-04 | 6·50E-01 | 7·04E-01 | 2 | NA | NA | 1·00E+00 | 1·00E+00 | 4 | NA | NA | 1·00E+00 | 1·00E+00 | 3 |
| Medication | binary | Antidepressants | NA | 3·12E-04 | 6·93E-04 | 6·25E-01 | 6·85E-01 | 2 | 2·51E-05 | 2·73E-04 | 8·75E-01 | 9·06E-01 | 4 | 7·32E-03 | 4·17E-03 | 5·07E-02 | 1·08E-01 | 3 |
| Neurodegenerative disorders and neuropathies | catological | Parkinson screening: Unsteady walking (self-reported) | NA | 1·08E-02 | 1·58E-02 | 2·06E-01 | 5·72E-01 | 2 | 3·51E-03 | 3·84E-03 | 6·05E-01 | 8·34E-01 | 4 | NA | NA | 1·00E+00 | 1·00E+00 | 3 |

| | | | | | | | | | | | | | | | | | | |
|---|---|---|---|---|---|---|---|---|---|---|---|---|---|---|---|---|---|---|
| Cancer | catological | Diagnosed cancer | NA | 2·53E-03 | 2·66E-03 | 3·79E-01 | 5·20E-01 | 2 | 1·52E-04 | 1·08E-03 | 9·28E-01 | 9·66E-01 | 4 | 3·71E-03 | 3·83E-03 | 3·81E-01 | 4·91E-01 | 3 |
| Medication | binary | Anticoagulants | NA | 1·00E-03 | 1·05E-03 | 3·81E-01 | 4·75E-01 | 2 | 3·66E-04 | 1·52E-03 | 5·48E-01 | 6·75E-01 | 4 | 2·54E-04 | 3·29E-04 | 7·17E-01 | 7·59E-01 | 3 |
| Medication | binary | Anti-dementia medication | NA | 3·26E-03 | 1·49E-03 | 1·14E-01 | 1·84E-01 | 2 | 1·59E-08 | 1·30E-04 | 9·97E-01 | 9·97E-01 | 4 | NA | NA | 1·00E+00 | 1·00E+00 | 3 |
| Medication | binary | Mirtazapine | NA | 1·09E-03 | 5·52E-04 | 3·61E-01 | 4·48E-01 | 2 | 9·92E-04 | 4·35E-04 | 3·23E-01 | 4·72E-01 | 4 | 1·29E-02 | 1·98E-03 | 9·46E-03 | 2·84E-02 | 3 |
| Medication | binary | Muscle relaxants | NA | 7·36E-04 | 2·25E-03 | 4·53E-01 | 5·31E-01 | 2 | 2·97E-03 | 1·44E-03 | 8·66E-02 | 2·31E-01 | 4 | 1·23E-02 | 2·45E-03 | 1·12E-02 | 3·02E-02 | 3 |
| Medication | binary | Muscle relaxants, centrally acting agents | NA | 7·36E-04 | 2·21E-03 | 4·53E-01 | 5·31E-01 | 2 | 2·97E-03 | 1·38E-03 | 8·66E-02 | 2·31E-01 | 4 | 1·23E-02 | 2·49E-03 | 1·12E-02 | 3·02E-02 | 3 |
| Sleep | continous | Sleep inertia on free days | 3·00E-17 | 1·93E-02 | 1·30E-02 | 2·83E-02 | 6·06E-02 | 2 | 8·27E-03 | 9·04E-03 | 8·80E-02 | 2·16E-01 | 4 | 2·40E-03 | 4·49E-03 | 4·20E-01 | 5·17E-01 | 3 |
| Cardiovascular system | binary | Year of the 1st stroke | NA | 6·54E-05 | 1·14E-03 | 8·24E-01 | 8·55E-01 | 2 | 5·39E-04 | 1·02E-03 | 4·69E-01 | 6·11E-01 | 4 | 3·37E-05 | 2·50E-04 | 8·96E-01 | 9·06E-01 | 3 |
| Baseline | binary | Fasting status on the day of examination with regard to food | NA | 2·08E-04 | 2·05E-04 | 6·90E-01 | 7·28E-01 | 2 | 5·27E-03 | 1·12E-03 | 2·23E-02 | 9·52E-02 | 4 | NA | NA | 1·00E+00 | 1·00E+00 | 3 |
| Medication | binary | Trizyclika | NA | 1·62E-03 | 1·14E-03 | 2·66E-01 | 3·53E-01 | 2 | 2·02E-03 | 1·45E-03 | 1·58E-01 | 3·07E-01 | 4 | 8·11E-04 | 7·72E-04 | 5·16E-01 | 5·78E-01 | 3 |
| Physical activity | catological | Duration of cycling on weekdays (self-reported) | NA | 5·53E-03 | 4·65E-03 | 3·74E-01 | 6·20E-01 | 2 | 3·94E-03 | 3·57E-03 | 4·20E-01 | 7·57E-01 | 4 | 5·09E-03 | 2·92E-03 | 6·18E-01 | 7·55E-01 | 3 |
| Hypothyroidism | ordinal | Diagnosed hypothyroidism | NA | 6·56E-03 | 5·24E-03 | 1·69E-01 | 3·62E-01 | 2 | 6·38E-03 | 3·38E-03 | 9·66E-02 | 3·93E-01 | 4 | 1·14E-02 | 5·47E-03 | 1·13E-01 | 2·40E-01 | 3 |
| Cholesterol and triglycerides | continous | Elevated cholesterol or triglyceride levels within last 12 month | 2·66E-01 | 1·69E-02 | 1·42E-02 | 5·21E-02 | 1·38E-01 | 2 | 9·84E-04 | 6·39E-03 | 5·73E-01 | 7·15E-01 | 4 | 2·69E-03 | 6·27E-03 | 4·91E-01 | 6·02E-01 | 3 |
| Medication | binary | Bisphosphonates | NA | 1·27E-03 | 5·68E-04 | 3·23E-01 | 4·25E-01 | 2 | 6·39E-04 | 6·47E-04 | 4·27E-01 | 5·68E-01 | 4 | 4·75E-03 | 1·14E-03 | 1·16E-01 | 1·78E-01 | 3 |
| Cholesterol and triglycerides | continous | HDL - cholesterol | 1·19E-09 | 1·33E-04 | 1·65E-03 | 7·49E-01 | 7·98E-01 | 2 | 7·85E-04 | 1·92E-03 | 3·79E-01 | 5·36E-01 | 4 | 5·94E-03 | 7·07E-03 | 7·85E-02 | 1·33E-01 | 3 |
| Physical activity | ordinal | Current physical condition (self-reported) | NA | 6·08E-03 | 5·81E-03 | 1·97E-01 | 4·00E-01 | 2 | 3·02E-03 | 2·42E-03 | 3·94E-01 | 6·85E-01 | 4 | 4·21E-03 | 3·79E-03 | 5·34E-01 | 6·65E-01 | 3 |
| Cardiovascular system | binary | Inpatient treated stroke (self-reported) | NA | 3·29E-04 | 1·08E-03 | 6·16E-01 | 6·78E-01 | 2 | 7·16E-04 | 9·47E-04 | 4·01E-01 | 5·44E-01 | 4 | 1·11E-04 | 7·39E-04 | 8·10E-01 | 8·38E-01 | 3 |
| Neurodegenerative disorders and neuropathies | ordinal | Parkinson screening: Feeling that the feet are stuck to the floor (self-reported) | NA | 1·31E-02 | 9·52E-03 | 5·66E-02 | 2·55E-01 | 2 | 3·04E-03 | 4·10E-03 | 3·56E-01 | 5·80E-01 | 4 | NA | NA | 1·00E+00 | 1·00E+00 | 3 |
| Physical activity | continous | Time usually spend outdoors in winter (self-reported) | 7·96E-28 | 7·03E-03 | 6·17E-03 | 2·00E-02 | 6·94E-02 | 2 | 1·05E-04 | 1·12E-03 | 7·48E-01 | 8·25E-01 | 4 | 1·68E-04 | 2·75E-03 | 7·68E-01 | 8·07E-01 | 3 |
| Diabetes | ordinal | Age at diagnosis of validated diabetes mellitus with type indication | NA | 1·42E-02 | 5·25E-03 | 5·64E-01 | 8·21E-01 | 2 | 2·08E-02 | 4·72E-03 | 6·40E-02 | 5·77E-01 | 4 | 1·83E-02 | 4·37E-03 | 1·65E-01 | 3·56E-01 | 3 |
| Physical activity | ordinal | Success rates of diets (self-reported) | NA | 6·92E-03 | 2·33E-03 | 2·56E-01 | 4·76E-01 | 2 | 7·41E-03 | 3·89E-03 | 1·18E-01 | 4·65E-01 | 4 | 3·45E-02 | 3·77E-03 | 1·05E-03 | 1·10E-02 | 3 |
| Medication | binary | Other antihypertensives (except diuretics, beta-blokers, ACE inhibitors, calcium and angiotensin antagonists) | NA | 2·28E-04 | 1·25E-03 | 6·76E-01 | 7·31E-01 | 2 | 1·17E-04 | 3·32E-04 | 7·34E-01 | 8·03E-01 | 4 | 4·28E-03 | 1·83E-03 | 1·35E-01 | 2·14E-01 | 3 |
| Inflammation | ordinal | Phlebitis in superficial veins within last 12 months | NA | 1·37E-03 | 3·57E-03 | 7·12E-01 | 7·76E-01 | 2 | 1·99E-03 | 2·08E-03 | 5·28E-01 | 6·96E-01 | 4 | 7·64E-04 | 5·77E-04 | 8·20E-01 | 8·73E-01 | 3 |
| Neurodegenerative disorders and neuropathies | catological | Diagnosed nerve damage (neuropathy, polyneuropathy or diabetic foot) within last 12 months | NA | 2·24E-04 | 5·66E-04 | 7·60E-01 | 8·16E-01 | 2 | 9·62E-07 | 3·69E-04 | 9·83E-01 | 9·87E-01 | 4 | 2·07E-02 | 5·61E-03 | 4·03E-02 | 1·33E-01 | 3 |
| Baseline | binary | Sex | NA | 0·00E+00 | 0·00E+00 | 1·00E+00 | 1·00E+00 | 2 | 5·97E-03 | 4·23E-03 | 1·49E-02 | 8·54E-02 | 4 | 6·00E-04 | 2·60E-03 | 5·77E-01 | 6·41E-01 | 3 |

**Supplementary table 6: Association analyses of dicarbonyls with lead SNPs of GWA-studies on BMI, glucose, GFR, GGT, and systolic blood pressure (SBP).**

2,385 leads SNPs of available previously published GWA studies on traits selected by RDA in both studies (Figures 2B and 2C) were selected for genotypic association analyses with the dicarbonyls MG, GO, and 3-DG. These analyses were done with an extended model that accounted for the dependency of the dicarbonyls on intake and excretion. One SNP (highlighted in green) overcame the Bonferroni-corrected significance threshold of 0·05/1,890 = 2·65E-05. The effective number of 1,890 was used for correction because some SNPs were in linkage disequilibrium. Nominally significant results are shown (p<0·05).

| SNP | Chr | Pos | EA | OA | Trait | eaf | beta | SE | p-value | Trait | eaf | beta | SE | p-value | Sample size | 2nd Trait | eaf | beta | SE | p-value | Sample size |
|---|---|---|---|---|---|---|---|---|---|---|---|---|---|---|---|---|---|---|---|---|---|
| rs1741177 | 14 | 35383219 | A | G | 3-DG | 0·684 | 0·053 | 0·012 | 2·33E-05 | eGFR | 0·703 | -0·002 | 0·000 | 8·82E-10 | 1156030 | | | | | | |
| rs10797067 | 1 | 154237638 | T | G | 3-DG | 0·369 | 0·041 | 0·012 | 9·85E-04 | GGT | 0·367 | 0·003 | 0·001 | 9·90E-10 | 437194 | | | | | | |
| rs1625427 | 11 | 131957293 | C | T | 3-DG | 0·346 | -0·039 | 0·012 | 1·08E-03 | BMI | 0·350 | -0·013 | 0·002 | 1·40E-12 | 676334 | | | | | | |
| rs11983783 | 7 | 96463812 | T | C | 3-DG | 0·581 | 0·037 | 0·012 | 1·34E-03 | SBP | 0·581 | 0·176 | 0·031 | 9·48E-09 | 728171 | | | | | | |
| rs11670142 | 19 | 49650471 | G | T | 3-DG | 0·418 | 0·037 | 0·012 | 1·36E-03 | BMI | 0·394 | 0·011 | 0·002 | 4·70E-10 | 681469 | | | | | | |
| rs183869 | 5 | 139531018 | T | C | 3-DG | 0·181 | -0·048 | 0·015 | 1·49E-03 | GGT | 0·172 | 0·004 | 0·001 | 5·00E-10 | 437194 | | | | | | |
| rs62055084 | 16 | 73097845 | T | C | 3-DG | 0·275 | -0·043 | 0·015 | 3·60E-03 | SBP | 0·268 | 0·297 | 0·037 | 5·94E-16 | 640761 | | | | | | |
| rs702395 | 5 | 140086677 | T | C | 3-DG | 0·442 | -0·033 | 0·011 | 3·66E-03 | SBP | 0·437 | 0·232 | 0·031 | 3·24E-14 | 727577 | | | | | | |
| rs2161097 | 5 | 103945178 | T | C | 3-DG | 0·433 | -0·033 | 0·012 | 4·02E-03 | BMI | 0·444 | 0·010 | 0·002 | 2·50E-08 | 692354 | | | | | | |
| rs12615778 | 2 | 102436738 | G | A | 3-DG | 0·327 | 0·035 | 0·012 | 4·31E-03 | BMI | 0·306 | -0·010 | 0·002 | 3·90E-08 | 684324 | | | | | | |
| rs1365466 | 18 | 36182440 | T | C | 3-DG | 0·722 | -0·036 | 0·013 | 4·92E-03 | BMI | 0·741 | -0·014 | 0·002 | 3·30E-13 | 791868 | | | | | | |
| rs2613765 | 19 | 5066330 | A | G | 3-DG | 0·491 | 0·033 | 0·012 | 4·97E-03 | SBP | 0·475 | -0·235 | 0·030 | 5·32E-15 | 738552 | | | | | | |
| rs10942267 | 5 | 80841914 | G | A | 3-DG | 0·289 | 0·036 | 0·013 | 5·06E-03 | BMI | 0·309 | -0·016 | 0·002 | 3·90E-17 | 689084 | | | | | | |
| rs11071738 | 15 | 63580155 | C | T | 3-DG | 0·485 | 0·033 | 0·012 | 5·30E-03 | eGFR | 0·450 | 0·003 | 0·000 | 1·59E-23 | 1201590 | | | | | | |
| rs12894354 | 14 | 102987884 | C | T | 3-DG | 0·631 | -0·034 | 0·012 | 5·47E-03 | eGFR | 0·653 | 0·002 | 0·000 | 2·98E-11 | 1150630 | | | | | | |
| rs10782230 | 6 | 126228512 | A | G | 3-DG | 0·498 | 0·032 | 0·012 | 5·85E-03 | SBP | 0·485 | 0·211 | 0·030 | 2·91E-12 | 732600 | | | | | | |
| rs35889227 | 14 | 90055468 | T | G | 3-DG | 0·603 | 0·034 | 0·012 | 6·43E-03 | Glucose | 0·617 | -0·013 | 0·002 | 3·37E-10 | 166896 | | | | | | |
| rs7535253 | 1 | 214744893 | C | T | 3-DG | 0·788 | 0·040 | 0·015 | 6·78E-03 | eGFR | 0·760 | -0·002 | 0·000 | 2·16E-13 | 1199770 | | | | | | |
| rs6587552 | 1 | 151018861 | G | A | 3-DG | 0·766 | 0·038 | 0·014 | 6·82E-03 | BMI | 0·759 | -0·017 | 0·002 | 1·60E-17 | 689723 | | | | | | |
| rs111230791 | 11 | 10270621 | C | T | 3-DG | 0·166 | 0·042 | 0·015 | 7·05E-03 | SBP | 0·186 | 0·478 | 0·039 | 5·17E-35 | 735584 | | | | | | |
| rs6468118 | 8 | 32399290 | G | C | 3-DG | 0·588 | 0·032 | 0·012 | 7·33E-03 | eGFR | 0·629 | 0·002 | 0·000 | 2·92E-15 | 1200740 | | | | | | |
| rs281380 | 19 | 49214470 | C | T | 3-DG | 0·387 | 0·032 | 0·012 | 7·40E-03 | eGFR | 0·405 | 0·002 | 0·000 | 8·72E-16 | 1151060 | | | | | | |
| rs818524 | 1 | 85201228 | C | T | 3-DG | 0·713 | 0·034 | 0·013 | 7·76E-03 | BMI | 0·694 | 0·011 | 0·002 | 3·40E-08 | 670078 | | | | | | |
| rs4516268 | 17 | 1846831 | A | C | 3-DG | 0·188 | -0·038 | 0·015 | 8·45E-03 | BMI | 0·193 | -0·022 | 0·002 | 5·20E-25 | 786617 | | | | | | |
| rs903959 | 8 | 142630782 | A | T | 3-DG | 0·410 | 0·031 | 0·012 | 8·74E-03 | BMI | 0·397 | 0·011 | 0·002 | 1·60E-09 | 689895 | | | | | | |
| rs217671 | 14 | 62360464 | G | A | 3-DG | 0·246 | -0·036 | 0·014 | 9·19E-03 | BMI | 0·272 | 0·014 | 0·002 | 1·30E-13 | 691456 | | | | | | |
| rs4858193 | 3 | 20441050 | C | T | 3-DG | 0·314 | 0·032 | 0·012 | 9·63E-03 | BMI | 0·278 | -0·013 | 0·002 | 1·60E-11 | 686850 | | | | | | |
| rs2354862 | 8 | 64501744 | C | A | 3-DG | 0·357 | -0·032 | 0·012 | 9·65E-03 | SBP | 0·359 | -0·251 | 0·032 | 2·42E-15 | 723156 | | | | | | |
| rs35530564 | 2 | 192112050 | G | T | 3-DG | 0·305 | -0·034 | 0·013 | 9·83E-03 | GGT | 0·324 | 0·011 | 0·001 | 4·50E-87 | 437194 | | | | | | |
| rs4980515 | 11 | 63744609 | C | T | 3-DG | 0·458 | 0·030 | 0·012 | 9·98E-03 | SBP | 0·501 | -0·225 | 0·030 | 9·73E-14 | 727998 | | | | | | |
| rs6557155 | 6 | 151910126 | G | T | 3-DG | 0·595 | 0·031 | 0·012 | 1·03E-02 | SBP | 0·599 | -0·191 | 0·031 | 1·14E-09 | 706941 | | | | | | |
| rs1879057 | 4 | 111335722 | T | C | 3-DG | 0·795 | -0·036 | 0·014 | 1·07E-02 | SBP | 0·795 | -0·325 | 0·038 | 1·67E-17 | 704405 | | | | | | |
| rs12033039 | 1 | 22257202 | T | G | 3-DG | 0·130 | -0·045 | 0·018 | 1·11E-02 | SBP | 0·107 | -0·270 | 0·049 | 4·89E-08 | 720556 | | | | | | |
| rs7963801 | 12 | 79685226 | C | T | 3-DG | 0·591 | 0·030 | 0·012 | 1·11E-02 | SBP | 0·578 | 0·236 | 0·031 | 2·87E-14 | 709837 | | | | | | |
| rs6991885 | 8 | 143500243 | C | T | 3-DG | 0·711 | 0·032 | 0·013 | 1·12E-02 | SBP | 0·693 | 0·183 | 0·033 | 3·20E-08 | 715747 | | | | | | |
| rs7209235 | 17 | 73759552 | A | G | 3-DG | 0·688 | 0·034 | 0·014 | 1·18E-02 | BMI | 0·695 | -0·011 | 0·002 | 8·00E-09 | 683445 | | | | | | |
| rs7703560 | 5 | 67678506 | G | A | 3-DG | 0·289 | 0·032 | 0·013 | 1·22E-02 | SBP | 0·300 | 0·225 | 0·033 | 1·51E-11 | 714892 | | | | | | |

| | | | | | | | | | | | | | | | | | | | | | |
|---|---|---|---|---|---|---|---|---|---|---|---|---|---|---|---|---|---|---|---|---|---|
| rs60580012 | 17 | 45969808 | T | C | 3-DG | 0·351 | -0·031 | 0·012 | 1·25E-02 | eGFR | 0·322 | -0·002 | 0·000 | 4·24E-12 | 1159620 | | | | | | |
| rs3814828 | 14 | 93406702 | G | A | 3-DG | 0·615 | -0·031 | 0·012 | 1·31E-02 | eGFR | 0·634 | -0·002 | 0·000 | 4·62E-15 | 1153390 | | | | | | |
| rs7324484 | 13 | 48648079 | A | G | 3-DG | 0·645 | 0·030 | 0·012 | 1·32E-02 | eGFR | 0·648 | 0·002 | 0·000 | 4·41E-15 | 1196300 | | | | | | |
| rs551619 | 11 | 78597949 | G | C | 3-DG | 0·344 | -0·031 | 0·013 | 1·48E-02 | GGT | 0·352 | 0·006 | 0·001 | 4·70E-30 | 437194 | | | | | | |
| rs11228824 | 11 | 56634316 | A | G | 3-DG | 0·206 | 0·035 | 0·014 | 1·53E-02 | BMI | 0·188 | -0·013 | 0·002 | 7·60E-09 | 679916 | | | | | | |
| rs7439567 | 4 | 138464842 | T | C | 3-DG | 0·397 | -0·028 | 0·012 | 1·54E-02 | SBP | 0·411 | 0·254 | 0·031 | 2·31E-16 | 723243 | | | | | | |
| rs7187540 | 16 | 85318302 | A | C | 3-DG | 0·318 | 0·033 | 0·014 | 1·54E-02 | SBP | 0·335 | -0·195 | 0·034 | 1·02E-08 | 657562 | | | | | | |
| rs2652812 | 15 | 63406170 | T | C | 3-DG | 0·736 | 0·032 | 0·013 | 1·56E-02 | SBP | 0·754 | -0·252 | 0·035 | 1·03E-12 | 727494 | | | | | | |
| rs34905952 | 3 | 138104635 | A | G | 3-DG | 0·168 | 0·037 | 0·015 | 1·57E-02 | SBP | 0·161 | 0·287 | 0·041 | 3·00E-12 | 729433 | | | | | | |
| rs11752007 | 6 | 7242605 | C | T | 3-DG | 0·114 | -0·046 | 0·019 | 1·57E-02 | GGT | 0·126 | 0·007 | 0·001 | 3·70E-18 | 437194 | | | | | | |
| rs6433243 | 2 | 171605495 | C | T | 3-DG | 0·667 | -0·030 | 0·012 | 1·70E-02 | BMI | 0·654 | -0·010 | 0·002 | 1·20E-08 | 691231 | | | | | | |
| rs34357137 | 6 | 41672041 | G | C | 3-DG | 0·239 | -0·034 | 0·014 | 1·70E-02 | eGFR | 0·269 | 0·003 | 0·000 | 1·25E-25 | 1159620 | | | | | | |
| rs1117080 | 20 | 43428323 | G | C | 3-DG | 0·284 | 0·032 | 0·013 | 1·85E-02 | BMI | 0·299 | -0·011 | 0·002 | 7·00E-09 | 690702 | | | | | | |
| rs78998485 | 12 | 434755 | G | C | 3-DG | 0·234 | -0·033 | 0·014 | 1·90E-02 | SBP | 0·256 | 0·245 | 0·035 | 1·48E-12 | 732650 | | | | | | |
| rs6849518 | 4 | 20120274 | T | C | 3-DG | 0·135 | -0·040 | 0·017 | 1·91E-02 | BMI | 0·126 | 0·017 | 0·003 | 2·60E-11 | 687979 | | | | | | |
| rs12762034 | 10 | 33969931 | C | T | 3-DG | 0·083 | 0·049 | 0·021 | 1·91E-02 | BMI | 0·076 | 0·024 | 0·003 | 7·30E-14 | 692191 | | | | | | |
| rs2198679 | 13 | 89131240 | G | A | 3-DG | 0·427 | -0·028 | 0·012 | 1·95E-02 | BMI | 0·468 | -0·011 | 0·002 | 5·60E-10 | 688014 | | | | | | |
| rs839755 | 1 | 43856410 | C | A | 3-DG | 0·366 | 0·029 | 0·013 | 2·06E-02 | SBP | 0·385 | 0·267 | 0·031 | 5·41E-18 | 736630 | | | | | | |
| rs7165200 | 15 | 66937421 | A | G | 3-DG | 0·436 | -0·028 | 0·012 | 2·11E-02 | SBP | 0·440 | 0·201 | 0·030 | 2·87E-11 | 738706 | | | | | | |
| rs28621435 | 12 | 13860990 | A | G | 3-DG | 0·107 | -0·046 | 0·020 | 2·13E-02 | SBP | 0·115 | -0·298 | 0·048 | 6·47E-10 | 720349 | | | | | | |
| rs1474698 | 6 | 56064197 | T | C | 3-DG | 0·528 | -0·026 | 0·011 | 2·15E-02 | SBP | 0·552 | -0·177 | 0·031 | 6·73E-09 | 724276 | | | | | | |
| rs17789218 | 6 | 100600097 | C | T | 3-DG | 0·208 | 0·039 | 0·017 | 2·17E-02 | BMI | 0·239 | 0·013 | 0·002 | 7·40E-12 | 793904 | | | | | | |
| rs946824 | 1 | 243684019 | C | T | 3-DG | 0·864 | 0·039 | 0·017 | 2·18E-02 | BMI | 0·859 | -0·021 | 0·003 | 1·10E-15 | 689849 | | | | | | |
| rs7826312 | 8 | 32400115 | C | T | 3-DG | 0·569 | 0·027 | 0·012 | 2·19E-02 | BMI | 0·588 | 0·010 | 0·002 | 4·90E-10 | 785343 | | | | | | |
| rs9747973 | 17 | 37905107 | T | C | 3-DG | 0·459 | 0·027 | 0·012 | 2·19E-02 | eGFR | 0·497 | 0·002 | 0·000 | 7·40E-09 | 1159620 | | | | | | |
| rs3811417 | 1 | 151804907 | C | T | 3-DG | 0·115 | -0·041 | 0·018 | 2·24E-02 | GGT | 0·133 | 0·006 | 0·001 | 1·10E-15 | 437194 | | | | | | |
| rs55834529 | 6 | 27072542 | G | A | 3-DG | 0·091 | 0·048 | 0·021 | 2·24E-02 | eGFR | 0·101 | -0·003 | 0·001 | 8·14E-11 | 1027640 | | | | | | |
| rs7405696 | 17 | 36102035 | C | G | 3-DG | 0·402 | 0·029 | 0·013 | 2·28E-02 | eGFR | 0·434 | -0·002 | 0·000 | 1·42E-08 | 1142060 | | | | | | |
| rs55944332 | 2 | 145726621 | G | A | 3-DG | 0·252 | -0·030 | 0·013 | 2·33E-02 | SBP | 0·237 | 0·261 | 0·036 | 1·79E-13 | 729179 | | | | | | |
| rs28374392 | 20 | 61189717 | T | C | 3-DG | 0·612 | -0·029 | 0·013 | 2·40E-02 | SBP | 0·623 | 0·192 | 0·034 | 1·21E-08 | 640744 | | | | | | |
| rs58117425 | 2 | 145681570 | A | G | 3-DG | 0·252 | -0·030 | 0·013 | 2·54E-02 | eGFR | 0·228 | -0·002 | 0·000 | 3·44E-09 | 1201810 | | | | | | |
| rs3860770 | 5 | 173301427 | A | G | 3-DG | 0·319 | 0·028 | 0·012 | 2·55E-02 | SBP | 0·292 | -0·266 | 0·033 | 1·20E-15 | 729395 | | | | | | |
| rs8142376 | 22 | 32001037 | T | C | 3-DG | 0·474 | -0·026 | 0·012 | 2·61E-02 | SBP | 0·491 | 0·168 | 0·030 | 2·19E-08 | 744681 | | | | | | |
| rs1891730 | 9 | 130309028 | C | T | 3-DG | 0·373 | 0·027 | 0·012 | 2·61E-02 | SBP | 0·383 | 0·181 | 0·031 | 7·74E-09 | 714729 | | | | | | |
| rs7412 | 19 | 45412079 | T | C | 3-DG | 0·087 | 0·047 | 0·021 | 2·63E-02 | SBP | 0·082 | -0·434 | 0·057 | 4·04E-14 | 696370 | | | | | | |
| rs236671 | 7 | 75061419 | G | A | 3-DG | 0·541 | -0·029 | 0·013 | 2·69E-02 | GGT | 0·580 | 0·004 | 0·001 | 3·50E-15 | 437194 | | | | | | |
| rs11690717 | 2 | 205093360 | T | G | 3-DG | 0·386 | -0·027 | 0·012 | 2·71E-02 | SBP | 0·373 | -0·202 | 0·032 | 1·92E-10 | 712479 | | | | | | |
| rs10433609 | 3 | 51311574 | A | T | 3-DG | 0·831 | -0·036 | 0·016 | 2·74E-02 | BMI | 0·832 | 0·016 | 0·002 | 1·70E-12 | 689684 | | | | | | |
| rs62477728 | 7 | 75132471 | C | G | 3-DG | 0·527 | -0·027 | 0·012 | 2·83E-02 | SBP | 0·555 | 0·179 | 0·032 | 1·52E-08 | 680539 | | | | | | |
| rs10744146 | 12 | 17212881 | A | G | 3-DG | 0·495 | -0·026 | 0·012 | 2·83E-02 | BMI | 0·515 | -0·012 | 0·002 | 1·40E-12 | 689141 | | | | | | |
| rs273504 | 19 | 18215247 | G | A | 3-DG | 0·404 | 0·026 | 0·012 | 2·84E-02 | BMI | 0·427 | 0·015 | 0·002 | 4·40E-18 | 690672 | | | | | | |
| rs11671389 | 19 | 18210922 | T | A | 3-DG | 0·232 | 0·031 | 0·014 | 2·85E-02 | GGT | 0·254 | 0·008 | 0·001 | 9·70E-41 | 437194 | | | | | | |
| rs13250058 | 8 | 112270826 | T | G | 3-DG | 0·678 | -0·027 | 0·012 | 2·86E-02 | BMI | 0·677 | 0·011 | 0·002 | 2·90E-10 | 787870 | | | | | | |
| rs4745794 | 10 | 77534000 | G | A | 3-DG | 0·484 | -0·026 | 0·012 | 2·87E-02 | BMI | 0·516 | 0·011 | 0·002 | 7·20E-11 | 678128 | | | | | | |
| rs907091 | 17 | 37921742 | T | C | 3-DG | 0·469 | 0·025 | 0·012 | 3·05E-02 | GGT | 0·486 | -0·003 | 0·001 | 1·40E-10 | 437194 | | | | | | |
| rs10447437 | 6 | 126258892 | G | A | 3-DG | 0·470 | 0·025 | 0·012 | 3·07E-02 | eGFR | 0·468 | 0·002 | 0·000 | 1·68E-09 | 1148220 | | | | | | |

| | | | | | | | | | | | | | | | | | | | | | |
|---|---|---|---|---|---|---|---|---|---|---|---|---|---|---|---|---|---|---|---|---|---|
| rs11619319 | 13 | 28487599 | G | A | 3-DG | 0·181 | 0·032 | 0·015 | 3·13E-02 | Glucose | 0·234 | 0·017 | 0·002 | 3·41E-20 | 197615 | | | | | | |
| rs1784460 | 11 | 118938371 | A | T | 3-DG | 0·393 | 0·026 | 0·012 | 3·16E-02 | BMI | 0·404 | 0·013 | 0·002 | 9·00E-14 | 680042 | | | | | | |
| rs11123169 | 2 | 113967075 | T | C | 3-DG | 0·645 | 0·028 | 0·013 | 3·17E-02 | eGFR | 0·675 | 0·003 | 0·000 | 2·73E-21 | 1195190 | | | | | | |
| rs7526754 | 1 | 209233764 | T | C | 3-DG | 0·632 | 0·026 | 0·012 | 3·19E-02 | BMI | 0·645 | -0·010 | 0·002 | 1·80E-08 | 675804 | | | | | | |
| rs7912283 | 10 | 133773019 | A | G | 3-DG | 0·610 | 0·026 | 0·012 | 3·22E-02 | SBP | 0·647 | -0·214 | 0·032 | 2·94E-11 | 703775 | | | | | | |
| rs580114 | 1 | 28566173 | A | G | 3-DG | 0·301 | 0·027 | 0·013 | 3·23E-02 | eGFR | 0·285 | -0·002 | 0·000 | 3·45E-11 | 1201900 | | | | | | |
| rs7123876 | 11 | 72444583 | C | T | 3-DG | 0·246 | 0·029 | 0·014 | 3·25E-02 | BMI | 0·246 | 0·012 | 0·002 | 2·20E-10 | 793551 | | | | | | |
| rs10015716 | 4 | 95548550 | A | G | 3-DG | 0·631 | 0·027 | 0·013 | 3·33E-02 | eGFR | 0·642 | 0·002 | 0·000 | 7·01E-09 | 1158670 | | | | | | |
| rs17695092 | 5 | 173337853 | G | T | 3-DG | 0·333 | 0·026 | 0·012 | 3·34E-02 | BMI | 0·308 | -0·011 | 0·002 | 3·40E-09 | 775102 | | | | | | |
| rs4319878 | 19 | 21924452 | T | C | 3-DG | 0·569 | -0·026 | 0·012 | 3·38E-02 | SBP | 0·561 | 0·169 | 0·031 | 3·77E-08 | 717097 | | | | | | |
| rs2293605 | 3 | 184044433 | T | C | 3-DG | 0·136 | -0·038 | 0·018 | 3·39E-02 | BMI | 0·131 | -0·017 | 0·003 | 3·60E-10 | 668940 | eGFR | 0·120 | 0·002 | 0·000 | 4·59E-09 | 1134700 |
| rs4812536 | 20 | 40258709 | G | A | 3-DG | 0·327 | 0·026 | 0·012 | 3·42E-02 | SBP | 0·342 | -0·271 | 0·032 | 4·09E-17 | 713714 | | | | | | |
| rs12460791 | 19 | 1842919 | G | A | 3-DG | 0·206 | 0·032 | 0·015 | 3·45E-02 | SBP | 0·208 | -0·230 | 0·038 | 1·11E-09 | 712694 | | | | | | |
| rs3935648 | 17 | 79085335 | G | C | 3-DG | 0·214 | -0·031 | 0·015 | 3·45E-02 | BMI | 0·233 | -0·013 | 0·002 | 6·80E-09 | 629303 | | | | | | |
| rs1336486 | 13 | 40784814 | G | T | 3-DG | 0·305 | -0·027 | 0·013 | 3·46E-02 | BMI | 0·321 | 0·014 | 0·002 | 1·80E-14 | 688180 | | | | | | |
| rs61373376 | 19 | 46183586 | T | C | 3-DG | 0·166 | 0·034 | 0·016 | 3·49E-02 | SBP | 0·158 | 0·230 | 0·042 | 4·54E-08 | 718900 | | | | | | |
| rs28590346 | 16 | 2080653 | T | A | 3-DG | 0·353 | -0·026 | 0·012 | 3·60E-02 | SBP | 0·340 | 0·271 | 0·033 | 3·37E-16 | 685757 | | | | | | |
| rs6804842 | 3 | 25106437 | G | A | 3-DG | 0·582 | -0·025 | 0·012 | 3·65E-02 | BMI | 0·572 | 0·016 | 0·002 | 3·60E-21 | 789179 | | | | | | |
| rs5742643 | 12 | 102837863 | C | T | 3-DG | 0·742 | -0·028 | 0·013 | 3·69E-02 | SBP | 0·751 | 0·223 | 0·035 | 1·53E-10 | 732162 | | | | | | |
| rs11915371 | 3 | 70539559 | C | A | 3-DG | 0·218 | -0·029 | 0·014 | 3·69E-02 | BMI | 0·204 | 0·015 | 0·002 | 2·60E-12 | 690829 | | | | | | |
| rs12148121 | 15 | 67494922 | C | T | 3-DG | 0·212 | -0·030 | 0·014 | 3·70E-02 | eGFR | 0·275 | -0·002 | 0·000 | 2·60E-11 | 1200950 | | | | | | |
| rs6864049 | 5 | 124330522 | G | A | 3-DG | 0·516 | -0·025 | 0·012 | 3·71E-02 | BMI | 0·539 | 0·013 | 0·002 | 6·70E-14 | 776397 | | | | | | |
| rs10163018 | 15 | 53525415 | T | C | 3-DG | 0·387 | 0·026 | 0·012 | 3·72E-02 | BMI | 0·375 | -0·011 | 0·002 | 1·50E-10 | 689715 | | | | | | |
| rs17207196 | 7 | 75101065 | T | C | 3-DG | 0·410 | 0·028 | 0·013 | 3·79E-02 | BMI | 0·412 | -0·022 | 0·002 | 2·10E-35 | 668894 | | | | | | |
| rs813412 | 10 | 32284825 | T | C | 3-DG | 0·755 | 0·028 | 0·014 | 3·83E-02 | SBP | 0·751 | -0·210 | 0·035 | 1·87E-09 | 730888 | | | | | | |
| rs138289 | 22 | 32182708 | T | A | 3-DG | 0·452 | -0·025 | 0·012 | 3·84E-02 | BMI | 0·483 | -0·010 | 0·002 | 3·30E-09 | 687258 | | | | | | |
| rs73039433 | 19 | 33524604 | A | G | 3-DG | 0·064 | 0·051 | 0·025 | 3·88E-02 | GGT | 0·047 | 0·015 | 0·001 | 9·70E-33 | 437194 | | | | | | |
| rs10144318 | 14 | 53189731 | C | A | 3-DG | 0·520 | -0·024 | 0·012 | 3·90E-02 | BMI | 0·511 | -0·009 | 0·002 | 1·40E-08 | 794528 | | | | | | |
| rs9816226 | 3 | 185834499 | T | A | 3-DG | 0·815 | -0·032 | 0·016 | 3·96E-02 | BMI | 0·820 | 0·032 | 0·002 | 1·60E-52 | 778333 | | | | | | |
| rs4683096 | 3 | 45374496 | G | T | 3-DG | 0·569 | -0·024 | 0·012 | 4·14E-02 | BMI | 0·561 | -0·012 | 0·002 | 2·80E-11 | 682633 | | | | | | |
| rs79539362 | 3 | 154680449 | C | T | 3-DG | 0·108 | -0·038 | 0·019 | 4·18E-02 | SBP | 0·101 | -0·400 | 0·050 | 2·09E-15 | 729915 | | | | | | |
| rs17806379 | 20 | 51107290 | T | C | 3-DG | 0·153 | 0·032 | 0·016 | 4·19E-02 | BMI | 0·179 | -0·026 | 0·002 | 1·50E-30 | 690043 | | | | | | |
| rs2842870 | 1 | 156200671 | C | T | 3-DG | 0·360 | 0·025 | 0·012 | 4·20E-02 | eGFR | 0·365 | 0·002 | 0·000 | 6·34E-11 | 1201620 | | | | | | |
| rs756717 | 16 | 72996162 | A | G | 3-DG | 0·387 | -0·026 | 0·013 | 4·22E-02 | BMI | 0·397 | -0·015 | 0·002 | 5·40E-18 | 771976 | | | | | | |
| rs3784924 | 16 | 11644842 | G | A | 3-DG | 0·292 | -0·027 | 0·013 | 4·29E-02 | GGT | 0·314 | 0·007 | 0·001 | 4·70E-34 | 437194 | | | | | | |
| rs3918226 | 7 | 150690176 | T | C | 3-DG | 0·077 | -0·046 | 0·023 | 4·33E-02 | SBP | 0·081 | 0·664 | 0·058 | 8·46E-31 | 680941 | eGFR | 0·083 | -0·003 | 0·001 | 2·51E-11 | 1017730 |
| rs55924785 | 9 | 136929586 | T | C | 3-DG | 0·183 | 0·030 | 0·015 | 4·35E-02 | eGFR | 0·162 | -0·002 | 0·000 | 1·54E-08 | 1135840 | | | | | | |
| rs779206 | 3 | 118023515 | A | G | 3-DG | 0·729 | 0·027 | 0·013 | 4·40E-02 | BMI | 0·734 | -0·013 | 0·002 | 1·10E-10 | 689160 | | | | | | |
| rs17257081 | 2 | 135630498 | G | A | 3-DG | 0·232 | -0·027 | 0·014 | 4·43E-02 | SBP | 0·194 | -0·227 | 0·039 | 6·35E-09 | 705214 | | | | | | |
| rs7560871 | 2 | 145616899 | A | G | 3-DG | 0·077 | -0·044 | 0·022 | 4·49E-02 | BMI | 0·073 | 0·022 | 0·003 | 9·60E-11 | 691571 | | | | | | |
| rs13245051 | 7 | 113362799 | G | A | 3-DG | 0·531 | -0·026 | 0·013 | 4·51E-02 | eGFR | 0·548 | 0·002 | 0·000 | 2·27E-13 | 1030500 | | | | | | |
| rs61311941 | 9 | 92285323 | G | A | 3-DG | 0·075 | -0·047 | 0·024 | 4·54E-02 | eGFR | 0·121 | 0·003 | 0·000 | 5·61E-10 | 1142110 | | | | | | |
| rs6056413 | 20 | 9106087 | T | A | 3-DG | 0·811 | -0·030 | 0·015 | 4·55E-02 | BMI | 0·829 | -0·013 | 0·002 | 1·10E-08 | 748884 | | | | | | |
| rs9326846 | 5 | 111301546 | G | A | 3-DG | 0·328 | 0·025 | 0·012 | 4·60E-02 | BMI | 0·311 | 0·011 | 0·002 | 7·10E-09 | 688531 | | | | | | |
| rs7249143 | 19 | 12992579 | T | G | 3-DG | 0·268 | -0·027 | 0·014 | 4·61E-02 | BMI | 0·308 | 0·013 | 0·002 | 3·60E-11 | 678777 | | | | | | |
| rs1048303 | 7 | 100804140 | T | C | 3-DG | 0·602 | 0·024 | 0·012 | 4·63E-02 | BMI | 0·586 | -0·011 | 0·002 | 2·70E-10 | 690582 | | | | | | |

| | | | | | | | | | | | | | | | | | | | | | |
|---|---|---|---|---|---|---|---|---|---|---|---|---|---|---|---|---|---|---|---|---|---|
| rs1075901 | 17 | 15943910 | C | T | 3-DG | 0·577 | -0·023 | 0·012 | 4·65E-02 | BMI | 0·564 | 0·012 | 0·002 | 1·20E-13 | 794789 | | | | | | |
| rs1133400 | 10 | 134459388 | G | A | 3-DG | 0·214 | 0·028 | 0·014 | 4·65E-02 | SBP | 0·214 | 0·298 | 0·038 | 2·53E-15 | 709605 | | | | | | |
| rs11000570 | 10 | 75025252 | C | G | 3-DG | 0·068 | -0·046 | 0·023 | 4·66E-02 | eGFR | 0·119 | -0·003 | 0·001 | 2·76E-08 | 1141960 | | | | | | |
| rs11856921 | 15 | 39278107 | A | C | 3-DG | 0·410 | -0·024 | 0·012 | 4·70E-02 | eGFR | 0·413 | 0·003 | 0·000 | 3·18E-25 | 1158520 | | | | | | |
| rs6921291 | 6 | 97066242 | T | C | 3-DG | 0·186 | -0·030 | 0·015 | 4·72E-02 | SBP | 0·191 | 0·358 | 0·039 | 1·58E-20 | 731010 | | | | | | |
| rs35599935 | 6 | 32886487 | C | T | 3-DG | 0·054 | 0·054 | 0·027 | 4·78E-02 | eGFR | 0·066 | 0·003 | 0·001 | 2·75E-09 | 1172510 | | | | | | |
| rs629864 | 11 | 100699139 | T | C | 3-DG | 0·623 | 0·023 | 0·012 | 4·80E-02 | SBP | 0·650 | -0·187 | 0·032 | 4·69E-09 | 720099 | | | | | | |
| rs825680 | 16 | 73606563 | T | A | 3-DG | 0·378 | -0·027 | 0·014 | 4·81E-02 | BMI | 0·417 | -0·010 | 0·002 | 6·70E-09 | 680731 | | | | | | |
| rs1808056 | 20 | 62890932 | A | G | 3-DG | 0·387 | 0·025 | 0·013 | 4·81E-02 | eGFR | 0·440 | 0·002 | 0·000 | 8·25E-11 | 1201620 | | | | | | |
| rs11672660 | 19 | 46180184 | T | C | 3-DG | 0·211 | 0·028 | 0·014 | 4·87E-02 | BMI | 0·205 | -0·034 | 0·002 | 1·70E-60 | 768426 | | | | | | |
| rs12149704 | 16 | 69789516 | A | G | 3-DG | 0·060 | -0·056 | 0·029 | 4·90E-02 | SBP | 0·063 | 0·588 | 0·068 | 8·56E-18 | 622172 | | | | | | |
| rs10750441 | 11 | 130469044 | T | C | 3-DG | 0·606 | -0·026 | 0·013 | 4·94E-02 | SBP | 0·662 | 0·175 | 0·032 | 3·74E-08 | 733371 | | | | | | |
| rs3741841 | 12 | 21695584 | G | A | 3-DG | 0·128 | 0·035 | 0·018 | 4·94E-02 | GGT | 0·138 | 0·005 | 0·001 | 5·00E-11 | 437194 | | | | | | |
| rs9436446 | 1 | 50590638 | A | G | 3-DG | 0·201 | 0·029 | 0·015 | 4·96E-02 | BMI | 0·219 | 0·017 | 0·002 | 4·00E-17 | 692423 | | | | | | |
| rs112175548 | 16 | 71754040 | C | T | 3-DG | 0·363 | 0·024 | 0·012 | 4·99E-02 | eGFR | 0·361 | 0·002 | 0·000 | 7·32E-14 | 1201910 | | | | | | |
| rs7784465 | 7 | 6418275 | C | T | GO | 0·144 | -0·049 | 0·013 | 8·47E-05 | BMI | 0·139 | 0·016 | 0·003 | 1·10E-10 | 685477 | | | | | | |
| rs35680304 | 7 | 130973495 | T | C | GO | 0·559 | 0·033 | 0·009 | 1·30E-04 | SBP | 0·593 | 0·269 | 0·031 | 3·76E-18 | 720141 | | | | | | |
| rs6767619 | 3 | 156893782 | C | G | GO | 0·335 | -0·029 | 0·009 | 1·30E-03 | BMI | 0·345 | 0·012 | 0·002 | 8·10E-11 | 691865 | | | | | | |
| rs243067 | 2 | 60621643 | T | G | GO | 0·436 | 0·028 | 0·009 | 1·37E-03 | eGFR | 0·396 | 0·001 | 0·000 | 1·74E-08 | 1049430 | | | | | | |
| rs6849518 | 4 | 20120274 | T | C | GO | 0·133 | -0·039 | 0·013 | 1·99E-03 | BMI | 0·126 | 0·017 | 0·003 | 2·60E-11 | 687979 | | | | | | |
| rs967532 | 4 | 128160430 | A | G | GO | 0·642 | 0·027 | 0·009 | 2·77E-03 | eGFR | 0·645 | -0·002 | 0·000 | 9·53E-13 | 1192700 | | | | | | |
| rs9989141 | 14 | 94006257 | T | C | GO | 0·649 | -0·027 | 0·009 | 2·78E-03 | BMI | 0·639 | 0·016 | 0·002 | 3·60E-21 | 752768 | | | | | | |
| rs12035349 | 1 | 77557339 | G | A | GO | 0·114 | 0·045 | 0·015 | 2·79E-03 | BMI | 0·130 | 0·019 | 0·003 | 1·10E-12 | 666990 | | | | | | |
| rs2979470 | 8 | 30288272 | C | T | GO | 0·523 | -0·026 | 0·009 | 2·99E-03 | SBP | 0·510 | -0·199 | 0·030 | 4·62E-11 | 727290 | | | | | | |
| rs17360994 | 1 | 27278573 | C | T | GO | 0·070 | 0·049 | 0·017 | 3·96E-03 | GGT | 0·080 | -0·007 | 0·001 | 2·10E-16 | 437194 | | | | | | |
| rs2865805 | 7 | 69318195 | C | A | GO | 0·270 | 0·028 | 0·010 | 4·62E-03 | BMI | 0·308 | 0·011 | 0·002 | 2·50E-09 | 691404 | | | | | | |
| rs2973564 | 5 | 73151033 | G | A | GO | 0·273 | 0·027 | 0·010 | 4·62E-03 | BMI | 0·284 | -0·011 | 0·002 | 3·30E-09 | 690248 | | | | | | |
| rs629864 | 11 | 100699139 | T | C | GO | 0·631 | 0·025 | 0·009 | 4·71E-03 | SBP | 0·650 | -0·187 | 0·032 | 4·69E-09 | 720099 | | | | | | |
| rs9521715 | 13 | 111015333 | T | G | GO | 0·223 | -0·030 | 0·011 | 5·33E-03 | GGT | 0·227 | 0·004 | 0·001 | 1·10E-10 | 437194 | | | | | | |
| rs571689 | 19 | 49207554 | T | C | GO | 0·465 | 0·024 | 0·009 | 5·73E-03 | SBP | 0·520 | 0·228 | 0·030 | 6·77E-14 | 727109 | | | | | | |
| rs1123571 | 1 | 2326009 | A | G | GO | 0·442 | -0·027 | 0·010 | 5·91E-03 | eGFR | 0·444 | 0·002 | 0·000 | 6·39E-09 | 1190600 | | | | | | |
| rs4980379 | 11 | 1888614 | T | C | GO | 0·371 | 0·026 | 0·010 | 6·07E-03 | SBP | 0·372 | 0·576 | 0·032 | 2·47E-72 | 701947 | | | | | | |
| rs3887080 | 12 | 121661966 | A | G | GO | 0·117 | -0·037 | 0·014 | 6·43E-03 | BMI | 0·121 | 0·018 | 0·003 | 5·10E-12 | 690624 | | | | | | |
| rs11785629 | 8 | 142367559 | A | G | GO | 0·694 | 0·027 | 0·010 | 6·65E-03 | SBP | 0·705 | -0·197 | 0·034 | 4·32E-09 | 714047 | | | | | | |
| rs8066234 | 17 | 17099717 | T | G | GO | 0·682 | -0·025 | 0·009 | 7·75E-03 | eGFR | 0·662 | 0·002 | 0·000 | 3·35E-09 | 1158670 | | | | | | |
| rs11619319 | 13 | 28487599 | G | A | GO | 0·189 | 0·030 | 0·011 | 7·83E-03 | Glucose | 0·234 | 0·017 | 0·002 | 3·41E-20 | 197615 | | | | | | |
| rs2781649 | 6 | 131876637 | G | A | GO | 0·231 | -0·028 | 0·010 | 7·93E-03 | eGFR | 0·241 | -0·003 | 0·000 | 6·20E-21 | 1120110 | | | | | | |
| rs303937 | 13 | 72372524 | A | T | GO | 0·376 | -0·023 | 0·009 | 8·75E-03 | eGFR | 0·436 | 0·003 | 0·000 | 2·73E-27 | 1159620 | | | | | | |
| rs17391694 | 1 | 78623626 | T | C | GO | 0·115 | 0·034 | 0·013 | 1·00E-02 | BMI | 0·119 | 0·032 | 0·003 | 7·50E-38 | 767720 | | | | | | |
| rs12369179 | 12 | 122963550 | T | C | GO | 0·097 | 0·040 | 0·016 | 1·01E-02 | BMI | 0·088 | -0·036 | 0·003 | 2·50E-31 | 674260 | | | | | | |
| rs1982441 | 8 | 28021769 | T | G | GO | 0·150 | -0·030 | 0·012 | 1·03E-02 | BMI | 0·138 | 0·018 | 0·003 | 7·00E-12 | 687705 | | | | | | |
| rs2954022 | 8 | 126482621 | A | C | GO | 0·470 | 0·022 | 0·009 | 1·04E-02 | GGT | 0·465 | -0·009 | 0·001 | 4·00E-65 | 437194 | | | | | | |
| rs1454148 | 3 | 176341188 | T | C | GO | 0·272 | 0·024 | 0·010 | 1·07E-02 | BMI | 0·267 | 0·011 | 0·002 | 4·60E-08 | 691136 | | | | | | |
| rs34460334 | 1 | 27215079 | C | T | GO | 0·071 | 0·042 | 0·017 | 1·12E-02 | eGFR | 0·085 | 0·003 | 0·001 | 2·10E-12 | 1043920 | | | | | | |
| rs12042924 | 1 | 197297417 | C | T | GO | 0·482 | -0·022 | 0·009 | 1·19E-02 | SBP | 0·472 | 0·181 | 0·030 | 2·62E-09 | 725176 | | | | | | |
| rs10942267 | 5 | 80841914 | G | A | GO | 0·285 | 0·024 | 0·010 | 1·19E-02 | BMI | 0·309 | -0·016 | 0·002 | 3·90E-17 | 689084 | | | | | | |

| | | | | | | | | | | | | | | | | | | | | | |
|---|---|---|---|---|---|---|---|---|---|---|---|---|---|---|---|---|---|---|---|---|---|
| rs1371108 | 2 | 81816251 | A | C | GO | 0·325 | -0·023 | 0·009 | 1·20E-02 | BMI | 0·325 | 0·012 | 0·002 | 9·00E-11 | 684620 | | | | | | |
| rs4839104 | 1 | 111708627 | A | G | GO | 0·106 | 0·034 | 0·014 | 1·25E-02 | GGT | 0·108 | -0·007 | 0·001 | 2·40E-20 | 437194 | | | | | | |
| rs2267161 | 22 | 30953295 | T | C | GO | 0·329 | 0·022 | 0·009 | 1·27E-02 | eGFR | 0·315 | 0·002 | 0·000 | 4·68E-11 | 1150400 | | | | | | |
| rs10151563 | 14 | 31721103 | A | G | GO | 0·610 | 0·022 | 0·009 | 1·36E-02 | eGFR | 0·621 | -0·002 | 0·000 | 1·09E-12 | 1201700 | | | | | | |
| rs7315980 | 12 | 53449321 | A | G | GO | 0·070 | 0·042 | 0·017 | 1·40E-02 | SBP | 0·083 | 0·480 | 0·056 | 7·35E-18 | 715521 | | | | | | |
| rs11187142 | 10 | 94468685 | C | T | GO | 0·883 | 0·034 | 0·014 | 1·42E-02 | SBP | 0·895 | -0·331 | 0·050 | 2·53E-11 | 725644 | | | | | | |
| rs1790998 | 7 | 134138380 | A | C | GO | 0·452 | -0·022 | 0·009 | 1·42E-02 | SBP | 0·454 | -0·209 | 0·031 | 9·09E-12 | 718632 | | | | | | |
| rs1006896 | 3 | 88104411 | C | A | GO | 0·095 | 0·036 | 0·015 | 1·43E-02 | BMI | 0·106 | -0·023 | 0·003 | 5·50E-18 | 691892 | | | | | | |
| rs911554 | 14 | 103999371 | T | G | GO | 0·380 | 0·022 | 0·009 | 1·46E-02 | eGFR | 0·397 | -0·002 | 0·000 | 1·78E-08 | 1153100 | | | | | | |
| rs2611774 | 17 | 56732304 | A | G | GO | 0·335 | -0·023 | 0·009 | 1·48E-02 | SBP | 0·338 | 0·219 | 0·032 | 6·85E-12 | 731230 | | | | | | |
| rs17767510 | 4 | 68156598 | C | G | GO | 0·173 | -0·029 | 0·012 | 1·51E-02 | BMI | 0·170 | 0·013 | 0·002 | 1·90E-08 | 683125 | | | | | | |
| rs6557780 | 8 | 21937441 | A | G | GO | 0·832 | -0·032 | 0·013 | 1·55E-02 | GGT | 0·835 | -0·013 | 0·001 | 4·70E-78 | 437194 | | | | | | |
| rs7539054 | 1 | 186618699 | C | A | GO | 0·260 | -0·024 | 0·010 | 1·66E-02 | eGFR | 0·290 | 0·002 | 0·000 | 2·79E-14 | 1199260 | | | | | | |
| rs194518 | 7 | 89853149 | A | G | GO | 0·558 | -0·020 | 0·009 | 1·67E-02 | Glucose | 0·520 | 0·010 | 0·002 | 8·76E-09 | 166893 | | | | | | |
| rs6968865 | 7 | 17287269 | T | A | GO | 0·641 | 0·022 | 0·009 | 1·71E-02 | eGFR | 0·596 | 0·002 | 0·000 | 1·13E-19 | 1197150 | | | | | | |
| rs9326846 | 5 | 111301546 | G | A | GO | 0·326 | 0·022 | 0·009 | 1·74E-02 | BMI | 0·311 | 0·011 | 0·002 | 7·10E-09 | 688531 | | | | | | |
| rs3757387 | 7 | 128576086 | C | T | GO | 0·411 | 0·021 | 0·009 | 1·77E-02 | eGFR | 0·430 | -0·003 | 0·000 | 6·54E-27 | 1158180 | SBP | 0·450 | -0·190 | 0·030 | 4·01E-10 | 726716 |
| rs10892873 | 11 | 122535333 | C | G | GO | 0·374 | 0·021 | 0·009 | 1·82E-02 | GGT | 0·368 | 0·004 | 0·001 | 1·30E-16 | 437194 | | | | | | |
| rs12065553 | 1 | 80793118 | G | A | GO | 0·277 | -0·022 | 0·010 | 1·87E-02 | BMI | 0·293 | 0·012 | 0·002 | 8·90E-10 | 691372 | | | | | | |
| rs1000294 | 8 | 23080135 | A | G | GO | 0·236 | 0·024 | 0·010 | 1·92E-02 | GGT | 0·247 | 0·004 | 0·001 | 3·10E-09 | 437194 | | | | | | |
| rs17236194 | 15 | 59002755 | C | T | GO | 0·134 | 0·030 | 0·013 | 1·92E-02 | BMI | 0·135 | 0·015 | 0·002 | 8·30E-11 | 790933 | | | | | | |
| rs486359 | 6 | 160774441 | C | G | GO | 0·493 | -0·020 | 0·009 | 1·96E-02 | BMI | 0·485 | 0·011 | 0·002 | 1·60E-11 | 770999 | | | | | | |
| rs75248620 | 11 | 9909127 | A | G | GO | 0·074 | -0·038 | 0·016 | 1·96E-02 | eGFR | 0·081 | 0·004 | 0·001 | 2·25E-13 | 1001420 | | | | | | |
| rs883541 | 17 | 66449122 | A | G | GO | 0·788 | -0·025 | 0·011 | 2·00E-02 | eGFR | 0·739 | -0·002 | 0·000 | 6·17E-19 | 1191470 | | | | | | |
| rs7562149 | 2 | 63008414 | C | T | GO | 0·332 | -0·021 | 0·009 | 2·03E-02 | GGT | 0·325 | 0·005 | 0·001 | 1·60E-21 | 437194 | | | | | | |
| rs8084719 | 18 | 46315420 | G | C | GO | 0·294 | -0·023 | 0·010 | 2·05E-02 | eGFR | 0·309 | 0·002 | 0·000 | 2·51E-08 | 1159620 | | | | | | |
| rs35021865 | 16 | 10618314 | T | C | GO | 0·096 | 0·035 | 0·015 | 2·09E-02 | GGT | 0·103 | -0·005 | 0·001 | 3·20E-08 | 437194 | | | | | | |
| rs6972171 | 7 | 156267032 | C | A | GO | 0·246 | 0·023 | 0·010 | 2·12E-02 | eGFR | 0·299 | 0·002 | 0·000 | 3·08E-18 | 1200360 | | | | | | |
| rs2907948 | 7 | 150638484 | A | G | GO | 0·220 | 0·024 | 0·010 | 2·12E-02 | BMI | 0·243 | -0·014 | 0·002 | 1·30E-13 | 794299 | | | | | | |
| rs4371687 | 4 | 69989998 | A | G | GO | 0·484 | -0·020 | 0·009 | 2·30E-02 | GGT | 0·482 | -0·004 | 0·001 | 2·80E-16 | 437194 | | | | | | |
| rs2160077 | 14 | 92428410 | A | G | GO | 0·443 | -0·019 | 0·008 | 2·32E-02 | BMI | 0·429 | 0·009 | 0·002 | 1·60E-08 | 795067 | | | | | | |
| rs11128904 | 3 | 18742270 | C | G | GO | 0·711 | 0·022 | 0·010 | 2·35E-02 | BMI | 0·727 | 0·011 | 0·002 | 4·50E-08 | 691222 | | | | | | |
| rs10815254 | 9 | 5630344 | T | C | GO | 0·269 | 0·022 | 0·010 | 2·44E-02 | GGT | 0·281 | -0·004 | 0·001 | 2·00E-13 | 437194 | | | | | | |
| rs6905288 | 6 | 43758873 | A | G | GO | 0·565 | 0·020 | 0·009 | 2·44E-02 | SBP | 0·568 | 0·190 | 0·031 | 1·09E-09 | 701811 | | | | | | |
| rs6968554 | 7 | 17287106 | G | A | GO | 0·647 | 0·020 | 0·009 | 2·46E-02 | BMI | 0·640 | 0·010 | 0·002 | 3·50E-09 | 794218 | | | | | | |
| rs3828783 | 6 | 33767727 | A | G | GO | 0·196 | -0·024 | 0·011 | 2·46E-02 | BMI | 0·181 | -0·017 | 0·002 | 5·60E-15 | 792749 | | | | | | |
| rs4665624 | 2 | 23875990 | C | A | GO | 0·392 | -0·021 | 0·010 | 2·49E-02 | SBP | 0·384 | -0·181 | 0·032 | 1·35E-08 | 694783 | | | | | | |
| rs2832283 | 21 | 30690558 | A | G | GO | 0·222 | 0·025 | 0·011 | 2·49E-02 | BMI | 0·221 | 0·012 | 0·002 | 5·80E-09 | 792324 | | | | | | |
| rs6851147 | 4 | 55056566 | G | C | GO | 0·274 | 0·022 | 0·010 | 2·51E-02 | SBP | 0·273 | 0·189 | 0·034 | 2·55E-08 | 724973 | | | | | | |
| rs3098186 | 15 | 50810621 | T | C | GO | 0·510 | 0·019 | 0·009 | 2·61E-02 | SBP | 0·516 | -0·242 | 0·030 | 1·41E-15 | 726862 | | | | | | |
| rs2126474 | 8 | 76878957 | T | G | GO | 0·401 | -0·020 | 0·009 | 2·62E-02 | SBP | 0·413 | -0·260 | 0·031 | 1·87E-17 | 735582 | | | | | | |
| rs6597650 | 9 | 133787225 | G | A | GO | 0·397 | -0·021 | 0·010 | 2·63E-02 | BMI | 0·406 | 0·012 | 0·002 | 7·90E-12 | 665828 | | | | | | |
| rs17216707 | 20 | 52732362 | C | T | GO | 0·194 | 0·026 | 0·012 | 2·64E-02 | eGFR | 0·190 | 0·005 | 0·000 | 6·88E-46 | 1146580 | | | | | | |
| rs1743259 | 6 | 118609205 | G | C | GO | 0·081 | -0·035 | 0·016 | 2·65E-02 | SBP | 0·081 | 0·398 | 0·056 | 8·87E-13 | 726510 | | | | | | |
| rs7165200 | 15 | 66937421 | A | G | GO | 0·429 | -0·020 | 0·009 | 2·72E-02 | SBP | 0·440 | 0·201 | 0·030 | 2·87E-11 | 738706 | | | | | | |
| rs72661887 | 1 | 38416310 | T | C | GO | 0·454 | 0·019 | 0·009 | 2·75E-02 | SBP | 0·453 | 0·246 | 0·031 | 3·88E-15 | 696848 | | | | | | |

| | | | | | | | | | | | | | | | | | | | | | |
|---|---|---|---|---|---|---|---|---|---|---|---|---|---|---|---|---|---|---|---|---|---|
| rs10487796 | 7 | 15063430 | A | T | GO | 0·465 | -0·019 | 0·009 | 2·77E-02 | Glucose | 0·484 | -0·026 | 0·002 | 4·62E-52 | 199033 | | | | | | |
| rs6766610 | 3 | 11664859 | A | C | GO | 0·541 | -0·019 | 0·009 | 2·78E-02 | BMI | 0·556 | -0·009 | 0·002 | 1·40E-08 | 777389 | | | | | | |
| rs281380 | 19 | 49214470 | C | T | GO | 0·393 | -0·020 | 0·009 | 2·79E-02 | eGFR | 0·405 | 0·002 | 0·000 | 8·72E-16 | 1151060 | | | | | | |
| rs3119304 | 6 | 160628557 | C | T | GO | 0·112 | -0·031 | 0·014 | 2·86E-02 | eGFR | 0·121 | -0·008 | 0·000 | 1·07E-96 | 1158180 | | | | | | |
| rs7172627 | 15 | 31877690 | G | A | GO | 0·474 | 0·019 | 0·009 | 2·88E-02 | BMI | 0·472 | 0·012 | 0·002 | 1·10E-11 | 690458 | | | | | | |
| rs9816226 | 3 | 185834499 | T | A | GO | 0·818 | -0·025 | 0·012 | 2·95E-02 | BMI | 0·820 | 0·032 | 0·002 | 1·60E-52 | 778333 | | | | | | |
| rs4666821 | 2 | 183077254 | T | G | GO | 0·499 | 0·019 | 0·009 | 3·00E-02 | eGFR | 0·521 | 0·002 | 0·000 | 7·10E-15 | 1199260 | | | | | | |
| rs13012099 | 2 | 47010742 | A | G | GO | 0·347 | 0·020 | 0·009 | 3·05E-02 | BMI | 0·341 | -0·014 | 0·002 | 1·20E-14 | 686541 | | | | | | |
| rs1519845 | 8 | 129916890 | G | A | GO | 0·397 | -0·019 | 0·009 | 3·13E-02 | eGFR | 0·376 | -0·002 | 0·000 | 1·64E-11 | 1196300 | | | | | | |
| rs1391980 | 4 | 129218126 | T | C | GO | 0·551 | -0·019 | 0·009 | 3·17E-02 | eGFR | 0·552 | 0·002 | 0·000 | 1·48E-08 | 1191600 | | | | | | |
| rs475805 | 11 | 65848738 | A | G | GO | 0·755 | -0·021 | 0·010 | 3·19E-02 | GGT | 0·765 | -0·004 | 0·001 | 6·60E-12 | 437194 | | | | | | |
| rs12153395 | 5 | 179411477 | A | G | GO | 0·122 | 0·029 | 0·013 | 3·20E-02 | SBP | 0·115 | -0·330 | 0·049 | 1·07E-11 | 700177 | | | | | | |
| rs742493 | 6 | 40998167 | C | T | GO | 0·132 | -0·028 | 0·013 | 3·21E-02 | GGT | 0·112 | -0·004 | 0·001 | 9·20E-09 | 437194 | | | | | | |
| rs17405819 | 8 | 76806584 | C | T | GO | 0·298 | -0·020 | 0·009 | 3·26E-02 | BMI | 0·301 | -0·022 | 0·002 | 4·30E-33 | 795493 | | | | | | |
| rs11056875 | 12 | 16470918 | C | T | GO | 0·849 | -0·025 | 0·012 | 3·27E-02 | BMI | 0·853 | -0·014 | 0·002 | 1·10E-08 | 691176 | | | | | | |
| rs2440452 | 8 | 6264879 | G | T | GO | 0·852 | -0·027 | 0·013 | 3·39E-02 | BMI | 0·866 | -0·014 | 0·003 | 4·30E-08 | 692117 | | | | | | |
| rs4854645 | 3 | 132204810 | A | G | GO | 0·484 | 0·019 | 0·009 | 3·46E-02 | eGFR | 0·483 | -0·002 | 0·000 | 1·33E-10 | 1201650 | | | | | | |
| rs2007231 | 1 | 115266306 | T | C | GO | 0·638 | -0·019 | 0·009 | 3·52E-02 | BMI | 0·639 | -0·010 | 0·002 | 5·20E-09 | 691969 | | | | | | |
| rs2273654 | 10 | 102689217 | C | T | GO | 0·454 | -0·019 | 0·009 | 3·53E-02 | SBP | 0·440 | -0·176 | 0·031 | 7·89E-09 | 725875 | | | | | | |
| rs962796 | 13 | 54385284 | C | T | GO | 0·792 | 0·022 | 0·011 | 3·57E-02 | BMI | 0·795 | -0·015 | 0·002 | 5·90E-12 | 692479 | | | | | | |
| rs6927301 | 6 | 154872080 | G | T | GO | 0·328 | -0·019 | 0·009 | 3·59E-02 | GGT | 0·315 | -0·004 | 0·001 | 1·10E-15 | 437194 | | | | | | |
| rs10069690 | 5 | 1279790 | T | C | GO | 0·244 | -0·023 | 0·011 | 3·60E-02 | SBP | 0·258 | 0·310 | 0·037 | 4·47E-17 | 654501 | | | | | | |
| rs10184839 | 2 | 181946115 | T | A | GO | 0·714 | 0·020 | 0·009 | 3·64E-02 | SBP | 0·709 | 0·185 | 0·033 | 2·40E-08 | 732550 | | | | | | |
| rs12448738 | 16 | 56489343 | C | A | GO | 0·130 | -0·027 | 0·013 | 3·67E-02 | BMI | 0·137 | 0·017 | 0·003 | 2·80E-11 | 691932 | | | | | | |
| rs7095788 | 10 | 95384152 | T | C | GO | 0·331 | 0·020 | 0·009 | 3·72E-02 | Glucose | 0·358 | -0·011 | 0·002 | 1·98E-09 | 178446 | | | | | | |
| rs2235564 | 1 | 6713114 | T | C | GO | 0·339 | 0·019 | 0·009 | 3·79E-02 | BMI | 0·347 | 0·013 | 0·002 | 3·70E-13 | 691544 | | | | | | |
| rs12950305 | 17 | 1361363 | G | A | GO | 0·693 | -0·020 | 0·010 | 3·87E-02 | SBP | 0·708 | 0·242 | 0·034 | 9·12E-13 | 710077 | | | | | | |
| rs2160236 | 2 | 40557276 | C | G | GO | 0·387 | 0·019 | 0·009 | 3·96E-02 | SBP | 0·380 | -0·244 | 0·032 | 1·22E-14 | 705705 | | | | | | |
| rs17085528 | 6 | 154876716 | G | T | GO | 0·191 | -0·022 | 0·011 | 4·01E-02 | eGFR | 0·182 | 0·002 | 0·000 | 2·08E-11 | 1194850 | | | | | | |
| rs4957026 | 5 | 361148 | G | A | GO | 0·666 | 0·019 | 0·009 | 4·05E-02 | SBP | 0·660 | -0·198 | 0·032 | 8·12E-10 | 716315 | | | | | | |
| rs927315 | 9 | 4117713 | T | C | GO | 0·477 | 0·018 | 0·009 | 4·09E-02 | SBP | 0·471 | 0·169 | 0·030 | 2·44E-08 | 725247 | | | | | | |
| rs1867324 | 5 | 112539955 | G | A | GO | 0·663 | 0·018 | 0·009 | 4·23E-02 | GGT | 0·681 | -0·004 | 0·001 | 7·40E-13 | 437194 | | | | | | |
| rs9522285 | 13 | 112230701 | A | G | GO | 0·411 | 0·018 | 0·009 | 4·27E-02 | BMI | 0·414 | 0·013 | 0·002 | 2·50E-13 | 690681 | | | | | | |
| rs2652812 | 15 | 63406170 | T | C | GO | 0·740 | 0·020 | 0·010 | 4·33E-02 | SBP | 0·754 | -0·252 | 0·035 | 1·03E-12 | 727494 | | | | | | |
| rs2246012 | 6 | 131898208 | C | T | GO | 0·179 | -0·024 | 0·012 | 4·34E-02 | BMI | 0·163 | 0·016 | 0·002 | 3·10E-13 | 795598 | | | | | | |
| rs62035088 | 15 | 81165842 | A | G | GO | 0·181 | -0·025 | 0·012 | 4·36E-02 | eGFR | 0·164 | 0·003 | 0·000 | 6·73E-16 | 1154210 | | | | | | |
| rs13174863 | 5 | 139080745 | G | A | GO | 0·148 | 0·026 | 0·013 | 4·36E-02 | BMI | 0·155 | 0·019 | 0·002 | 2·90E-16 | 773762 | | | | | | |
| rs2270778 | 3 | 124692689 | T | C | GO | 0·567 | -0·018 | 0·009 | 4·41E-02 | BMI | 0·584 | -0·010 | 0·002 | 4·30E-09 | 794903 | | | | | | |
| rs17713396 | 2 | 227201 | T | C | GO | 0·361 | -0·018 | 0·009 | 4·45E-02 | eGFR | 0·338 | -0·002 | 0·000 | 1·61E-17 | 1200810 | | | | | | |
| rs634501 | 5 | 180218668 | G | A | GO | 0·758 | 0·022 | 0·011 | 4·46E-02 | GGT | 0·734 | -0·004 | 0·001 | 5·90E-12 | 437194 | | | | | | |
| rs16871902 | 5 | 3488462 | A | G | GO | 0·485 | 0·017 | 0·009 | 4·50E-02 | BMI | 0·488 | 0·013 | 0·002 | 4·60E-13 | 690830 | | | | | | |
| rs8096658 | 18 | 77156537 | G | C | GO | 0·493 | 0·020 | 0·010 | 4·50E-02 | eGFR | 0·472 | -0·005 | 0·000 | 1·88E-75 | 1059950 | | | | | | |
| rs7340705 | 3 | 53734443 | C | T | GO | 0·314 | 0·018 | 0·009 | 4·55E-02 | SBP | 0·327 | 0·243 | 0·032 | 4·87E-14 | 727632 | | | | | | |
| rs329122 | 5 | 133864599 | A | G | GO | 0·419 | -0·018 | 0·009 | 4·62E-02 | BMI | 0·423 | -0·013 | 0·002 | 4·30E-14 | 792745 | | | | | | |
| rs6419734 | 3 | 78458928 | C | T | GO | 0·836 | 0·024 | 0·012 | 4·63E-02 | BMI | 0·848 | -0·017 | 0·003 | 1·90E-12 | 688250 | | | | | | |
| rs7117842 | 11 | 122534504 | C | T | GO | 0·375 | 0·018 | 0·009 | 4·65E-02 | BMI | 0·379 | 0·011 | 0·002 | 6·30E-11 | 784222 | | | | | | |

| | | | | | | | | | | | | | | | | | | | | | |
|---|---|---|---|---|---|---|---|---|---|---|---|---|---|---|---|---|---|---|---|---|---|
| rs62187537 | 20 | 1333060 | T | C | GO | 0·058 | -0·038 | 0·019 | 4·66E-02 | eGFR | 0·067 | 0·004 | 0·001 | 2·07E-11 | 1039570 | | | | | | |
| rs2419077 | 2 | 71648425 | T | G | GO | 0·262 | -0·020 | 0·010 | 4·72E-02 | SBP | 0·260 | 0·197 | 0·034 | 1·07E-08 | 729470 | | | | | | |
| rs2274786 | 9 | 112225774 | C | T | GO | 0·358 | 0·018 | 0·009 | 4·81E-02 | eGFR | 0·355 | -0·002 | 0·000 | 2·79E-09 | 1158520 | | | | | | |
| rs1575290 | 6 | 7715689 | T | C | GO | 0·469 | -0·017 | 0·009 | 4·82E-02 | SBP | 0·473 | 0·197 | 0·030 | 5·59E-11 | 735850 | | | | | | |
| rs6792725 | 3 | 24520283 | G | A | GO | 0·661 | 0·019 | 0·010 | 4·85E-02 | GGT | 0·692 | -0·003 | 0·001 | 3·50E-10 | 437194 | | | | | | |
| rs665731 | 11 | 114032484 | T | C | GO | 0·180 | 0·024 | 0·012 | 4·88E-02 | eGFR | 0·184 | -0·002 | 0·000 | 1·36E-10 | 1174480 | | | | | | |
| rs13110266 | 4 | 162129844 | A | G | GO | 0·405 | 0·017 | 0·009 | 4·93E-02 | BMI | 0·407 | -0·012 | 0·002 | 1·90E-12 | 791087 | | | | | | |
| rs911554 | 14 | 103999371 | T | G | MG | 0·382 | 0·039 | 0·010 | 1·12E-04 | eGFR | 0·397 | -0·002 | 0·000 | 1·78E-08 | 1153100 | | | | | | |
| rs11708118 | 3 | 160110255 | A | G | MG | 0·251 | 0·039 | 0·011 | 3·29E-04 | GGT | 0·262 | 0·007 | 0·001 | 3·10E-35 | 437194 | | | | | | |
| rs9294260 | 6 | 83433228 | A | G | MG | 0·475 | 0·033 | 0·010 | 5·83E-04 | BMI | 0·473 | 0·015 | 0·002 | 1·80E-19 | 783533 | | | | | | |
| rs12148121 | 15 | 67494922 | C | T | MG | 0·222 | -0·040 | 0·012 | 6·01E-04 | eGFR | 0·275 | -0·002 | 0·000 | 2·60E-11 | 1200950 | | | | | | |
| rs2241358 | 19 | 14588476 | G | C | MG | 0·317 | 0·040 | 0·012 | 7·15E-04 | eGFR | 0·313 | 0·002 | 0·000 | 6·16E-10 | 1144310 | | | | | | |
| rs9894634 | 17 | 1967501 | T | C | MG | 0·603 | 0·034 | 0·010 | 8·75E-04 | eGFR | 0·572 | -0·002 | 0·000 | 3·66E-14 | 1200730 | | | | | | |
| rs9807214 | 18 | 42782952 | A | G | MG | 0·310 | 0·036 | 0·011 | 8·79E-04 | eGFR | 0·292 | -0·002 | 0·000 | 2·73E-13 | 1201920 | | | | | | |
| rs10282122 | 7 | 2529623 | T | C | MG | 0·658 | -0·034 | 0·010 | 9·55E-04 | SBP | 0·668 | -0·302 | 0·033 | 2·46E-20 | 706826 | | | | | | |
| rs3808356 | 7 | 100492322 | T | C | MG | 0·279 | -0·034 | 0·011 | 1·46E-03 | SBP | 0·284 | 0·197 | 0·034 | 4·91E-09 | 720360 | | | | | | |
| rs2065418 | 11 | 30422068 | G | T | MG | 0·349 | -0·032 | 0·010 | 1·52E-03 | BMI | 0·362 | -0·017 | 0·002 | 3·60E-20 | 691707 | | | | | | |
| rs8075273 | 17 | 61728881 | A | C | MG | 0·295 | -0·033 | 0·011 | 1·97E-03 | BMI | 0·282 | -0·013 | 0·002 | 3·70E-13 | 795026 | | | | | | |
| rs6972171 | 7 | 156267032 | C | A | MG | 0·245 | 0·035 | 0·011 | 2·04E-03 | eGFR | 0·299 | 0·002 | 0·000 | 3·08E-18 | 1200360 | | | | | | |
| rs1811081 | 17 | 61996506 | A | C | MG | 0·252 | -0·036 | 0·012 | 2·18E-03 | GGT | 0·244 | -0·003 | 0·001 | 5·20E-09 | 437194 | | | | | | |
| rs1006896 | 3 | 88104411 | C | A | MG | 0·097 | 0·050 | 0·016 | 2·38E-03 | BMI | 0·106 | -0·023 | 0·003 | 5·50E-18 | 691892 | | | | | | |
| rs2246754 | 15 | 96767312 | T | C | MG | 0·208 | 0·035 | 0·012 | 2·57E-03 | SBP | 0·211 | 0·250 | 0·037 | 1·17E-11 | 735372 | | | | | | |
| rs4577304 | 2 | 73403040 | C | T | MG | 0·488 | 0·028 | 0·010 | 3·45E-03 | SBP | 0·477 | 0·177 | 0·030 | 4·99E-09 | 726869 | | | | | | |
| rs11753995 | 6 | 160575366 | A | G | MG | 0·163 | 0·037 | 0·013 | 3·62E-03 | eGFR | 0·166 | 0·004 | 0·000 | 1·63E-22 | 1035970 | | | | | | |
| rs11071738 | 15 | 63580155 | C | T | MG | 0·468 | 0·029 | 0·010 | 3·89E-03 | eGFR | 0·450 | 0·003 | 0·000 | 1·59E-23 | 1201590 | | | | | | |
| rs12042924 | 1 | 197297417 | C | T | MG | 0·467 | -0·029 | 0·010 | 4·23E-03 | SBP | 0·472 | 0·181 | 0·030 | 2·62E-09 | 725176 | | | | | | |
| rs12615778 | 2 | 102436738 | G | A | MG | 0·316 | 0·029 | 0·010 | 4·26E-03 | BMI | 0·306 | -0·010 | 0·002 | 3·90E-08 | 684324 | | | | | | |
| rs662138 | 6 | 160564476 | G | C | MG | 0·171 | 0·036 | 0·013 | 4·90E-03 | GGT | 0·186 | -0·005 | 0·001 | 1·10E-12 | 437194 | | | | | | |
| rs7539054 | 1 | 186618699 | C | A | MG | 0·258 | -0·032 | 0·011 | 5·19E-03 | eGFR | 0·290 | 0·002 | 0·000 | 2·79E-14 | 1199260 | | | | | | |
| rs248139 | 5 | 167352783 | A | G | MG | 0·185 | -0·035 | 0·013 | 5·39E-03 | BMI | 0·193 | 0·013 | 0·002 | 1·20E-09 | 683675 | | | | | | |
| rs762147 | 21 | 39238610 | G | A | MG | 0·739 | -0·031 | 0·011 | 5·75E-03 | BMI | 0·729 | 0·012 | 0·002 | 3·30E-09 | 692496 | | | | | | |
| rs3829849 | 9 | 129390800 | T | C | MG | 0·344 | 0·031 | 0·011 | 6·44E-03 | BMI | 0·359 | 0·010 | 0·002 | 5·90E-09 | 793851 | | | | | | |
| rs17420882 | 1 | 100818728 | G | T | MG | 0·295 | -0·029 | 0·011 | 6·70E-03 | eGFR | 0·286 | -0·002 | 0·000 | 5·06E-20 | 1196300 | | | | | | |
| rs12614953 | 2 | 43421461 | C | T | MG | 0·483 | -0·029 | 0·011 | 6·85E-03 | eGFR | 0·504 | 0·003 | 0·000 | 4·79E-24 | 1159620 | | | | | | |
| rs2652812 | 15 | 63406170 | T | C | MG | 0·743 | 0·030 | 0·011 | 7·24E-03 | SBP | 0·754 | -0·252 | 0·035 | 1·03E-12 | 727494 | | | | | | |
| rs1337101 | 1 | 219726100 | T | G | MG | 0·286 | -0·027 | 0·010 | 7·82E-03 | GGT | 0·317 | -0·003 | 0·001 | 1·10E-09 | 437194 | | | | | | |
| rs13240600 | 7 | 99064466 | G | A | MG | 0·144 | 0·036 | 0·014 | 8·20E-03 | BMI | 0·155 | -0·020 | 0·002 | 3·50E-17 | 692233 | | | | | | |
| rs12628891 | 22 | 38317137 | T | C | MG | 0·304 | 0·029 | 0·011 | 8·54E-03 | BMI | 0·317 | -0·011 | 0·002 | 3·00E-09 | 686575 | | | | | | |
| rs6851147 | 4 | 55056566 | G | C | MG | 0·270 | 0·029 | 0·011 | 9·05E-03 | SBP | 0·273 | 0·189 | 0·034 | 2·55E-08 | 724973 | | | | | | |
| rs2275848 | 9 | 95887320 | T | G | MG | 0·797 | -0·033 | 0·013 | 1·10E-02 | eGFR | 0·811 | 0·002 | 0·000 | 5·04E-10 | 1132270 | | | | | | |
| rs72869573 | 4 | 78830584 | T | C | MG | 0·130 | 0·036 | 0·014 | 1·15E-02 | GGT | 0·146 | 0·004 | 0·001 | 7·50E-09 | 437194 | | | | | | |
| rs7154723 | 14 | 98590629 | A | G | MG | 0·394 | 0·025 | 0·010 | 1·19E-02 | SBP | 0·385 | 0·253 | 0·031 | 2·72E-16 | 738875 | | | | | | |
| rs1528435 | 2 | 181550962 | T | C | MG | 0·628 | 0·025 | 0·010 | 1·33E-02 | BMI | 0·633 | 0·016 | 0·002 | 9·10E-23 | 794198 | | | | | | |
| rs2906163 | 7 | 2516594 | T | C | MG | 0·633 | -0·026 | 0·011 | 1·34E-02 | eGFR | 0·665 | 0·001 | 0·000 | 2·81E-08 | 1153920 | | | | | | |
| rs4952668 | 2 | 43386568 | A | G | MG | 0·634 | -0·026 | 0·010 | 1·39E-02 | SBP | 0·623 | -0·248 | 0·031 | 2·60E-15 | 721184 | | | | | | |
| rs10224210 | 7 | 151413194 | C | T | MG | 0·276 | 0·028 | 0·011 | 1·40E-02 | eGFR | 0·277 | -0·008 | 0·000 | 1·57E-139 | 978562 | SBP | 0·279 | 0·383 | 0·034 | 1·60E-29 | 717138 |

| | | | | | | | | | | | | | | | | | | | | | |
|---|---|---|---|---|---|---|---|---|---|---|---|---|---|---|---|---|---|---|---|---|---|
| rs62173695 | 2 | 178122727 | A | G | MG | 0·341 | -0·026 | 0·011 | 1·42E-02 | eGFR | 0·349 | 0·003 | 0·000 | 3·27E-35 | 1159620 | | | | | | |
| rs813412 | 10 | 32284825 | T | C | MG | 0·747 | 0·027 | 0·011 | 1·43E-02 | SBP | 0·751 | -0·210 | 0·035 | 1·87E-09 | 730888 | | | | | | |
| rs2272168 | 7 | 99102777 | C | G | MG | 0·142 | 0·034 | 0·014 | 1·43E-02 | GGT | 0·152 | -0·004 | 0·001 | 1·30E-08 | 437194 | | | | | | |
| rs28850910 | 11 | 27291960 | A | G | MG | 0·299 | 0·026 | 0·011 | 1·49E-02 | SBP | 0·319 | -0·207 | 0·032 | 1·66E-10 | 730267 | | | | | | |
| rs12190287 | 6 | 134214525 | G | C | MG | 0·376 | 0·025 | 0·010 | 1·53E-02 | eGFR | 0·383 | -0·002 | 0·000 | 2·02E-11 | 1157380 | | | | | | |
| rs57946343 | 10 | 63499951 | C | T | MG | 0·148 | -0·033 | 0·014 | 1·54E-02 | SBP | 0·147 | -0·716 | 0·043 | 2·10E-63 | 731715 | | | | | | |
| rs9373263 | 6 | 140553414 | A | G | MG | 0·234 | 0·027 | 0·011 | 1·57E-02 | GGT | 0·204 | -0·004 | 0·001 | 4·40E-10 | 437194 | | | | | | |
| rs8092503 | 18 | 52479487 | G | A | MG | 0·219 | 0·028 | 0·012 | 1·63E-02 | BMI | 0·232 | 0·017 | 0·002 | 2·30E-17 | 777619 | | | | | | |
| rs62325228 | 4 | 54318751 | T | G | MG | 0·137 | 0·034 | 0·014 | 1·67E-02 | eGFR | 0·144 | 0·002 | 0·000 | 3·33E-10 | 1201910 | | | | | | |
| rs8904 | 14 | 35871217 | A | G | MG | 0·379 | -0·024 | 0·010 | 1·67E-02 | SBP | 0·368 | 0·306 | 0·031 | 1·71E-22 | 728260 | | | | | | |
| rs12939549 | 17 | 78611724 | G | A | MG | 0·437 | -0·023 | 0·010 | 1·70E-02 | BMI | 0·434 | -0·018 | 0·002 | 2·70E-28 | 793950 | | | | | | |
| rs6265 | 11 | 27679916 | T | C | MG | 0·198 | -0·028 | 0·012 | 1·73E-02 | BMI | 0·195 | -0·041 | 0·002 | 1·00E-86 | 795458 | | | | | | |
| rs55754617 | 22 | 19128559 | T | C | MG | 0·203 | 0·028 | 0·012 | 1·77E-02 | eGFR | 0·191 | 0·002 | 0·000 | 1·07E-08 | 1159620 | | | | | | |
| rs5402 | 3 | 170727739 | A | T | MG | 0·124 | 0·036 | 0·015 | 1·78E-02 | GGT | 0·121 | 0·016 | 0·001 | 5·70E-95 | 437194 | | | | | | |
| rs427943 | 21 | 46570896 | C | A | MG | 0·535 | 0·023 | 0·010 | 1·94E-02 | BMI | 0·567 | 0·017 | 0·002 | 7·30E-23 | 712095 | | | | | | |
| rs10433609 | 3 | 51311574 | A | T | MG | 0·830 | -0·030 | 0·013 | 1·95E-02 | BMI | 0·832 | 0·016 | 0·002 | 1·70E-12 | 689684 | | | | | | |
| rs9812319 | 3 | 136238860 | T | C | MG | 0·335 | -0·023 | 0·010 | 2·09E-02 | eGFR | 0·335 | 0·002 | 0·000 | 1·12E-17 | 1196300 | | | | | | |
| rs6879279 | 5 | 106878792 | C | G | MG | 0·154 | -0·032 | 0·014 | 2·14E-02 | GGT | 0·154 | -0·012 | 0·001 | 6·90E-61 | 437194 | | | | | | |
| rs10746963 | 9 | 77238558 | G | A | MG | 0·808 | 0·029 | 0·013 | 2·25E-02 | SBP | 0·817 | 0·218 | 0·039 | 2·05E-08 | 738423 | | | | | | |
| rs1047891 | 2 | 211540507 | A | C | MG | 0·289 | -0·025 | 0·011 | 2·28E-02 | eGFR | 0·304 | -0·007 | 0·000 | 4·91E-116 | 1158670 | SBP | 0·319 | -0·253 | 0·033 | 1·37E-14 | 711099 |
| rs4480845 | 17 | 1958609 | C | T | MG | 0·629 | 0·024 | 0·011 | 2·34E-02 | SBP | 0·635 | -0·316 | 0·032 | 1·85E-23 | 720266 | | | | | | |
| rs6137201 | 20 | 20957669 | T | A | MG | 0·115 | -0·035 | 0·016 | 2·42E-02 | SBP | 0·122 | -0·267 | 0·047 | 1·64E-08 | 702173 | | | | | | |
| rs11663085 | 18 | 782898 | G | A | MG | 0·089 | 0·040 | 0·018 | 2·52E-02 | GGT | 0·105 | 0·005 | 0·001 | 9·80E-09 | 437194 | | | | | | |
| rs1076485 | 11 | 116772441 | T | C | MG | 0·138 | -0·031 | 0·014 | 2·52E-02 | SBP | 0·130 | 0·339 | 0·046 | 1·19E-13 | 715764 | | | | | | |
| rs2061813 | 16 | 7071254 | T | A | MG | 0·740 | -0·025 | 0·011 | 2·58E-02 | BMI | 0·742 | -0·011 | 0·002 | 3·20E-08 | 678583 | | | | | | |
| rs12575164 | 11 | 128349430 | C | T | MG | 0·198 | 0·027 | 0·012 | 2·58E-02 | eGFR | 0·215 | 0·002 | 0·000 | 1·90E-10 | 1201410 | | | | | | |
| rs1021066 | 8 | 106368050 | G | T | MG | 0·523 | -0·021 | 0·010 | 2·61E-02 | BMI | 0·524 | 0·010 | 0·002 | 2·50E-08 | 690198 | | | | | | |
| rs1365466 | 18 | 36182440 | T | C | MG | 0·724 | -0·023 | 0·011 | 2·71E-02 | BMI | 0·741 | -0·014 | 0·002 | 3·30E-13 | 791868 | | | | | | |
| rs7784465 | 7 | 6418275 | C | T | MG | 0·138 | -0·032 | 0·014 | 2·72E-02 | BMI | 0·139 | 0·016 | 0·003 | 1·10E-10 | 685477 | | | | | | |
| rs13270267 | 8 | 11444837 | T | C | MG | 0·448 | -0·022 | 0·010 | 2·74E-02 | eGFR | 0·464 | 0·002 | 0·000 | 1·04E-16 | 1046490 | | | | | | |
| rs7042372 | 9 | 6959840 | G | A | MG | 0·336 | -0·022 | 0·010 | 2·84E-02 | BMI | 0·343 | -0·012 | 0·002 | 3·80E-11 | 681234 | | | | | | |
| rs3746778 | 20 | 61341472 | A | G | MG | 0·400 | -0·027 | 0·012 | 2·85E-02 | GGT | 0·420 | -0·004 | 0·001 | 5·90E-11 | 437194 | | | | | | |
| rs754466 | 10 | 79680434 | T | A | MG | 0·277 | -0·024 | 0·011 | 2·93E-02 | GGT | 0·253 | 0·017 | 0·001 | 6·90E-192 | 437194 | | | | | | |
| rs6011457 | 20 | 61530915 | A | T | MG | 0·494 | -0·021 | 0·010 | 2·96E-02 | BMI | 0·498 | -0·012 | 0·002 | 2·70E-11 | 690692 | | | | | | |
| rs28374392 | 20 | 61189717 | T | C | MG | 0·600 | -0·025 | 0·011 | 3·00E-02 | SBP | 0·623 | 0·192 | 0·034 | 1·21E-08 | 640744 | | | | | | |
| rs7095788 | 10 | 95384152 | T | C | MG | 0·335 | 0·023 | 0·011 | 3·01E-02 | Glucose | 0·358 | -0·011 | 0·002 | 1·98E-09 | 178446 | | | | | | |
| rs227426 | 14 | 70456664 | T | G | MG | 0·532 | -0·021 | 0·010 | 3·02E-02 | SBP | 0·561 | 0·186 | 0·030 | 8·59E-10 | 734458 | | | | | | |
| rs6519133 | 22 | 39096602 | C | T | MG | 0·398 | -0·022 | 0·010 | 3·04E-02 | GGT | 0·395 | 0·007 | 0·001 | 6·70E-40 | 437194 | | | | | | |
| rs80282103 | 10 | 899071 | T | A | MG | 0·084 | 0·039 | 0·018 | 3·09E-02 | eGFR | 0·087 | -0·008 | 0·001 | 2·91E-62 | 1201760 | | | | | | |
| rs2731684 | 5 | 171861204 | A | C | MG | 0·398 | 0·022 | 0·010 | 3·14E-02 | GGT | 0·378 | -0·003 | 0·001 | 4·50E-09 | 437194 | | | | | | |
| rs1215469 | 13 | 80707408 | C | A | MG | 0·769 | 0·025 | 0·012 | 3·27E-02 | SBP | 0·770 | 0·212 | 0·037 | 6·16E-09 | 709331 | | | | | | |
| rs7963577 | 12 | 82174115 | C | T | MG | 0·235 | 0·024 | 0·011 | 3·44E-02 | eGFR | 0·215 | -0·002 | 0·000 | 2·02E-11 | 1163680 | | | | | | |
| rs33996239 | 1 | 203109801 | T | C | MG | 0·053 | -0·050 | 0·024 | 3·46E-02 | SBP | 0·060 | -0·366 | 0·066 | 3·39E-08 | 677241 | | | | | | |
| rs7966357 | 12 | 51209838 | G | C | MG | 0·309 | -0·023 | 0·011 | 3·52E-02 | eGFR | 0·330 | -0·002 | 0·000 | 1·74E-11 | 1201800 | | | | | | |
| rs1792 | 13 | 99088006 | A | G | MG | 0·246 | -0·025 | 0·012 | 3·55E-02 | GGT | 0·225 | 0·004 | 0·001 | 3·80E-09 | 437194 | | | | | | |
| rs6963840 | 7 | 78144371 | T | C | MG | 0·149 | 0·028 | 0·013 | 3·61E-02 | BMI | 0·211 | 0·015 | 0·002 | 3·40E-10 | 637209 | | | | | | |

| | | | | | | | | | | | | | | | | | | | | | |
|---|---|---|---|---|---|---|---|---|---|---|---|---|---|---|---|---|---|---|---|---|---|
| rs72830437 | 10 | 111960190 | A | G | MG | 0·066 | -0·040 | 0·019 | 3·65E-02 | eGFR | 0·070 | -0·003 | 0·001 | 2·99E-08 | 1201660 | | | | | | |
| rs676387 | 17 | 40706273 | A | C | MG | 0·259 | 0·023 | 0·011 | 3·66E-02 | GGT | 0·285 | 0·003 | 0·001 | 2·00E-08 | 437194 | | | | | | |
| rs7235205 | 18 | 63373762 | G | A | MG | 0·298 | 0·022 | 0·011 | 3·68E-02 | BMI | 0·266 | 0·014 | 0·002 | 4·40E-14 | 789016 | | | | | | |
| rs4812536 | 20 | 40258709 | G | A | MG | 0·333 | 0·021 | 0·010 | 3·76E-02 | SBP | 0·342 | -0·271 | 0·032 | 4·09E-17 | 713714 | | | | | | |
| rs2276153 | 11 | 58407740 | G | C | MG | 0·248 | -0·024 | 0·011 | 3·80E-02 | SBP | 0·243 | -0·330 | 0·035 | 6·56E-21 | 734236 | | | | | | |
| rs17457340 | 20 | 10640042 | C | T | MG | 0·072 | 0·040 | 0·019 | 3·86E-02 | GGT | 0·076 | -0·006 | 0·001 | 1·30E-09 | 437194 | | | | | | |
| rs6453319 | 5 | 76847499 | C | A | MG | 0·567 | -0·021 | 0·010 | 3·90E-02 | eGFR | 0·574 | -0·001 | 0·000 | 3·85E-08 | 973658 | | | | | | |
| rs7541882 | 1 | 64121569 | C | A | MG | 0·172 | 0·026 | 0·013 | 4·01E-02 | GGT | 0·190 | 0·004 | 0·001 | 1·80E-09 | 437194 | | | | | | |
| rs28578714 | 22 | 50727921 | C | T | MG | 0·392 | -0·024 | 0·012 | 4·10E-02 | SBP | 0·394 | -0·207 | 0·033 | 2·53E-10 | 666583 | | | | | | |
| rs11641374 | 16 | 1347717 | A | C | MG | 0·602 | -0·025 | 0·012 | 4·14E-02 | SBP | 0·600 | -0·194 | 0·031 | 3·26E-10 | 726309 | | | | | | |
| rs13107261 | 4 | 63768826 | A | G | MG | 0·365 | -0·021 | 0·010 | 4·16E-02 | SBP | 0·369 | -0·178 | 0·031 | 1·57E-08 | 723080 | | | | | | |
| rs1465900 | 11 | 76473138 | C | A | MG | 0·231 | -0·023 | 0·011 | 4·21E-02 | BMI | 0·219 | -0·013 | 0·002 | 4·80E-10 | 779748 | | | | | | |
| rs7600699 | 2 | 156018263 | G | C | MG | 0·165 | -0·027 | 0·013 | 4·23E-02 | BMI | 0·158 | 0·015 | 0·002 | 6·40E-10 | 691202 | | | | | | |
| rs72722767 | 8 | 129205103 | A | G | MG | 0·294 | 0·021 | 0·010 | 4·24E-02 | SBP | 0·298 | -0·180 | 0·033 | 4·64E-08 | 732239 | | | | | | |
| rs9436446 | 1 | 50590638 | A | G | MG | 0·210 | 0·025 | 0·012 | 4·24E-02 | BMI | 0·219 | 0·017 | 0·002 | 4·00E-17 | 692423 | | | | | | |
| rs10189186 | 2 | 53025757 | G | A | MG | 0·445 | 0·020 | 0·010 | 4·25E-02 | SBP | 0·470 | -0·189 | 0·030 | 3·91E-10 | 727116 | | | | | | |
| rs1813937 | 11 | 50468801 | T | C | MG | 0·782 | -0·025 | 0·012 | 4·25E-02 | eGFR | 0·750 | 0·002 | 0·000 | 1·75E-09 | 1173960 | | | | | | |
| rs10962549 | 9 | 16719445 | T | C | MG | 0·159 | 0·027 | 0·014 | 4·29E-02 | BMI | 0·168 | 0·020 | 0·002 | 2·50E-17 | 692271 | | | | | | |
| rs56213443 | 3 | 160309043 | A | C | MG | 0·545 | 0·019 | 0·010 | 4·30E-02 | SBP | 0·536 | -0·174 | 0·031 | 1·39E-08 | 717702 | | | | | | |
| rs7120873 | 11 | 49459474 | T | C | MG | 0·119 | 0·030 | 0·015 | 4·36E-02 | BMI | 0·111 | 0·017 | 0·003 | 3·40E-10 | 691850 | | | | | | |
| rs4912637 | 5 | 141720261 | A | G | MG | 0·333 | -0·021 | 0·011 | 4·36E-02 | BMI | 0·346 | -0·010 | 0·002 | 1·50E-08 | 691647 | | | | | | |
| rs263041 | 3 | 183522231 | G | A | MG | 0·643 | -0·020 | 0·010 | 4·37E-02 | BMI | 0·633 | -0·012 | 0·002 | 2·20E-12 | 692268 | | | | | | |
| rs11607862 | 11 | 35244574 | T | C | MG | 0·281 | -0·022 | 0·011 | 4·40E-02 | GGT | 0·288 | 0·003 | 0·001 | 9·80E-09 | 437194 | | | | | | |
| rs13432055 | 2 | 56603985 | C | T | MG | 0·265 | 0·023 | 0·011 | 4·46E-02 | BMI | 0·286 | 0·012 | 0·002 | 8·90E-11 | 790223 | | | | | | |
| rs2913920 | 5 | 141726983 | T | C | MG | 0·745 | -0·023 | 0·011 | 4·50E-02 | SBP | 0·765 | 0·242 | 0·036 | 1·62E-11 | 720146 | | | | | | |
| rs4430672 | 14 | 63094407 | C | T | MG | 0·806 | -0·025 | 0·013 | 4·56E-02 | BMI | 0·800 | -0·013 | 0·002 | 3·90E-09 | 691400 | | | | | | |
| rs62187537 | 20 | 1333060 | T | C | MG | 0·063 | -0·040 | 0·020 | 4·62E-02 | eGFR | 0·067 | 0·004 | 0·001 | 2·07E-11 | 1039570 | | | | | | |
| rs2607775 | 3 | 14220095 | G | C | MG | 0·480 | -0·020 | 0·010 | 4·67E-02 | eGFR | 0·479 | -0·002 | 0·000 | 3·82E-09 | 1201900 | | | | | | |
| rs17115145 | 14 | 30122409 | T | C | MG | 0·386 | 0·020 | 0·010 | 4·67E-02 | SBP | 0·397 | 0·178 | 0·031 | 7·39E-09 | 737491 | | | | | | |
| rs11114051 | 12 | 109167803 | C | T | MG | 0·302 | 0·021 | 0·011 | 4·79E-02 | GGT | 0·304 | -0·007 | 0·001 | 2·00E-34 | 437194 | | | | | | |
| rs10015716 | 4 | 95548550 | A | G | MG | 0·646 | 0·020 | 0·010 | 4·81E-02 | eGFR | 0·642 | 0·002 | 0·000 | 7·01E-09 | 1158670 | | | | | | |
| rs17405819 | 8 | 76806584 | C | T | MG | 0·302 | -0·021 | 0·011 | 4·83E-02 | BMI | 0·301 | -0·022 | 0·002 | 4·30E-33 | 795493 | | | | | | |
| rs28735420 | 17 | 12139964 | G | T | MG | 0·059 | 0·047 | 0·024 | 4·83E-02 | eGFR | 0·071 | -0·003 | 0·001 | 1·91E-10 | 1152640 | | | | | | |
| rs2608882 | 17 | 73934130 | A | G | MG | 0·221 | -0·023 | 0·012 | 4·87E-02 | SBP | 0·224 | -0·252 | 0·036 | 3·43E-12 | 733270 | | | | | | |
| rs268263 | 2 | 164954174 | A | T | MG | 0·749 | 0·022 | 0·011 | 4·87E-02 | SBP | 0·750 | 0·594 | 0·035 | 1·77E-63 | 713147 | | | | | | |
| rs10184839 | 2 | 181946115 | T | A | MG | 0·718 | 0·021 | 0·011 | 4·89E-02 | SBP | 0·709 | 0·185 | 0·033 | 2·40E-08 | 732550 | | | | | | |
| rs10501410 | 11 | 72088806 | A | G | MG | 0·082 | -0·035 | 0·018 | 4·91E-02 | SBP | 0·069 | 0·412 | 0·061 | 1·10E-11 | 717203 | | | | | | |
| rs10757831 | 9 | 29282192 | T | C | MG | 0·184 | -0·024 | 0·012 | 4·91E-02 | BMI | 0·188 | 0·012 | 0·002 | 2·60E-08 | 691189 | | | | | | |
| rs6662924 | 1 | 100894419 | A | C | MG | 0·179 | 0·024 | 0·012 | 4·92E-02 | Glucose | 0·198 | 0·014 | 0·002 | 3·34E-10 | 165515 | | | | | | |
| rs10144318 | 14 | 53189731 | C | A | MG | 0·524 | -0·019 | 0·010 | 4·94E-02 | BMI | 0·511 | -0·009 | 0·002 | 1·40E-08 | 794528 | | | | | | |
| rs907183 | 8 | 8729761 | C | G | MG | 0·449 | -0·020 | 0·010 | 4·98E-02 | SBP | 0·463 | 0·319 | 0·031 | 2·15E-25 | 724589 | | | | | | |
